# Non-duality in brain and experience of advanced meditators—key role for intrinsic neural timescales

**DOI:** 10.1038/s42003-026-10473-9

**Published:** 2026-06-12

**Authors:** Malipeddi Saketh, Arun Sasidharan, Bianca Ventura, Rahul Venugopal, Clemens Christian Bauer, Prejaas K. B. Tewarie, Ravindra P. Nagendra, Seema Mehrotra, John P. John, Balachundhar Subramaniam, Steven Laureys, Bindu M. Kutty, Georg Northoff

**Affiliations:** 1 Centre for Consciousness Studies, Department of Neurophysiology, Old Administrative Block, National Institute of Mental Health and Neuro Sciences (NIMHANS), Bengaluru, India; 2 School of Psychology, University of Ottawa, Ottawa, ON Canada; 3 McGovern Institute for Brain Research, Massachusetts Institute of Technology, Cambridge, MA USA; 4 Joint International Research Unit on Consciousness, CERVO Brain Research Centre, University of Laval, Québec City, QC Canada; 5 Sir Peter Mansfield Imaging Centre, School of Physics, University of Nottingham, Nottingham, UK; 6 Department of Clinical Psychology, National Institute of Mental Health and Neuro Sciences (NIMHANS), Bengaluru, India; 7 Multi-modal Brain Image Analysis Laboratory, Department of Psychiatry, National Institute of Mental Health and Neuro Sciences (NIMHANS), Bengaluru, India; 8 Sadhguru Center for a Conscious Planet, Department of Anesthesiology, Critical Care and Pain Medicine, Beth Israel Deaconess Medical Center, Brookline, MA USA; 9 Joint International Research Unit on Neuroplasticity, University of Liège, Liège, Belgium; 10 Centre for Cognition and Brain Disorders, Hangzhou Normal University, Hangzhou, China; 11 Present Address: The Royal’s Institute of Mental Health Research & University of Ottawa, Brain and Mind Research Institute, Centre for Neural Dynamics, University of Ottawa, Ottawa, ON Canada

**Keywords:** Consciousness, Neurophysiology

## Abstract

Distinguishing between self (internal) and environment (external) is fundamental to human experience, yet mind-body traditions describe non-dual states where this boundary dissolves. However, the neural basis of non-duality remains underexplored. We investigate this in advanced and novice meditators from the Isha Yoga tradition and meditation-naïve controls using psychological scales and EEG-based intrinsic neural timescales (INT). Subjects perform breath-watching meditation (internal attention) and a cognitive task (external attention), allowing us to operationalize non-duality as reduced internal-external distinction. Key findings include: (a) advanced meditators report stronger non-dual experiences during breath-watching than novices and controls; (b) across participants, INTs are longer during internal than external attention; (c) advanced meditators show similar INT durations between internal and external attention; and (d) this reduced INT difference correlates with stronger reported non-dual experiences. These results suggest that similar duration of intrinsic neural timescales during internal and external attention may serve as a neural signature of non-duality.

## Abstract (web summary)

Advanced meditators show similar duration of intrinsic neural timescales during internal and external attention, suggesting a neural signature of non-dual awareness.

## Introduction

Duality is a fundamental aspect of human experience, characterized by the distinction between internal and external phenomena. The internal phenomena are associated with self and self-related processing, while the external realm pertains to the world^1–4^. Ordinary states of consciousness are structured around this internal-external distinction, resulting in a dual organization of mental life. This is different in non-ordinary states of consciousness, like meditation. These show that human consciousness is not confined to this dualistic framework; it also includes states that transcend such divisions as manifest in non-dual awareness^5–14^. Deeply rooted in many contemplative, philosophical and spiritual traditions as diverse as Advaita Vedanta, Kashmiri Shaivism, Mahayana, and Vajrayana Buddhist schools^8,12,15–17^, the experience of non-dual awareness is characterized by the dissolution of the internal and external duality^6,12,14,18–24^. An emerging body of literature shows that advanced meditators report experiencing non-dual awareness^5,6,10,11,18,20,21,24–30^, often accompanied by experiential qualities such as a sense of presence, clarity, emptiness, bliss, and a timeless feeling, among others^30^. For example, one participant in a study on mindfulness-induced selflessness described the experience as follows: “It was emptiness, as if the self fell out of the picture. There was an experience, but it had no address; it was not attached to a center or subject…”^18^. These phenomenological reports suggest that non-dual awareness involves a fundamental alteration in the structure of experience, particularly in terms of the dissolution of the usual dual self–world boundaries. However, the neural mechanisms underlying such non-dual states remain poorly understood. What are the neural correlates of such non-dual awareness? Addressing this yet open question is the goal of our paper.

What do we mean by the concepts of duality and non-dual awareness? We use the framework provided by Josipovic, 2021^30^: “duality refers here to structuring of experience and knowing in terms of subject–object dichotomy. It also refers, more broadly, to the fragmentation of experience into conceptually reified polarities such as self–other, us–them, good–bad, internal–external and mind–body. Nonduality then primarily refers to experiencing and knowing without such dualities.” An emerging body of literature shows that advanced meditators do indeed report experiencing such non-dual states^5,6,10,11,18,20,21,24–28^; this has been described by the concepts of non-duality and non-dual awareness, which we here use interchangeably^5,9,30^.

Spiritual traditions describe two broad types of deep meditative experiences during non-dual awareness^8,31,32^: 1) stages of full absorption with minimal phenomenal content^8,25,27,33^, and 2) stages of full absorption with minimal phenomenal content which, additionally, are accompanied by an inherent non-conceptual awareness^34^, i.e., an awareness that knows itself to be aware without conceptual representation^9^ (as distinguished from the more conceptual meta-awareness per se^30,35^). Here, we briefly outline these two perspectives in the literature regarding how these experiences are related to and often interpreted as non-dual awareness (for more information, please see refs. ^8,36^).

**Fig. 1:**
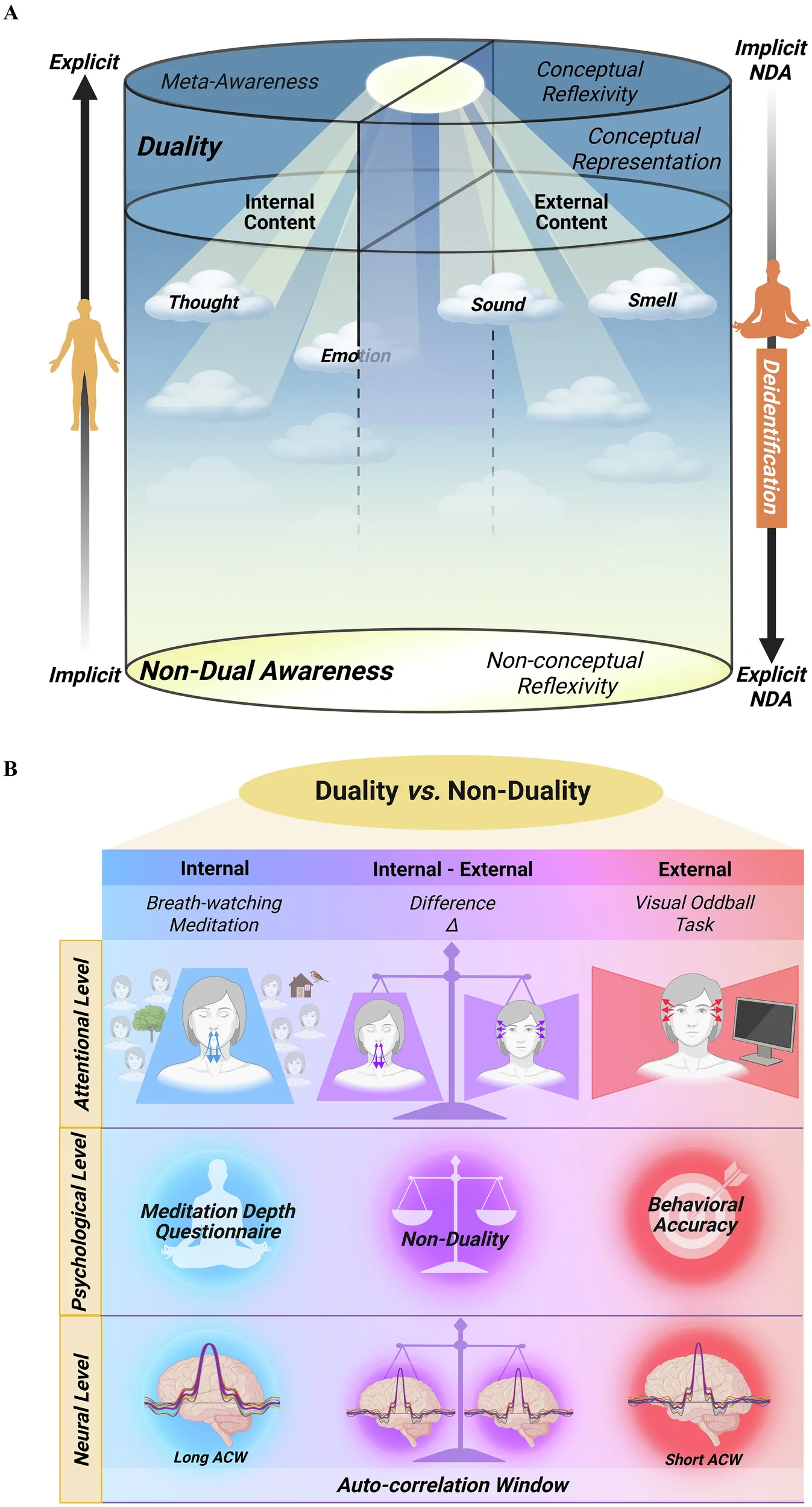
Schematic illustration of nondual awareness and study hypotheses. **A** The duality layer represents the ordinary structure of experience, divided between internal (e.g., thought, emotion) and external (e.g., sound, smell) contents, here illustrated as clouds that obscure the underlying ground of awareness. This layer is governed by conceptual representation. Beneath this layer lies non-dual awareness, portrayed as a mirror-like base, which is the fundamental, non-conceptual reflexivity that reflects all experience without dividing it into internal–external dualities. The implicit–explicit gradient on the right indicates that non-dual awareness (NDA) is always implicitly present, even during wakefulness or sleep, but becomes explicitly manifest only when the dualistic clouds of conceptualization and content distinction dissolve through deidentification. Meta-awareness is depicted as the light at the top, symbolizing conceptual reflexivity, i.e., the capacity to become aware of one’s own mental contents, yet still within an internal-external dual and representational mode of knowing. **B** The study conceptualizes duality as the difference between internal and external, and non-duality as the reduction or dissolution of this internal-external distinction. Duality and non-duality are examined across three hierarchical levels in our study. At the attentional level, internal focus is probed through breath-watching meditation and external focus through a visual oddball task. The reduction in their difference (Δ) indexes the degree of non-duality. At the psychological level, the meditation depth questionnaire (MEDEQ) and accuracy quantify the degree of internal vs. external attention, while the non-duality cluster of the MEDEQ reflects the experiential dimension of non-dual awareness^38^. At the neural level, the auto-correlation window (ACW) measures the duration of intrinsic neural timescales: a longer ACW is associated with internally oriented states, while a shorter ACW occurs in externally oriented states. A reduced difference between internal and external ACW indicates a neural transition toward non-dual awareness. Created in BioRender. Saketh, M. (2026) https://BioRender.com/udw4d4m.

According to the first perspective, both experiences, i.e., minimal phenomenal content and non-conceptual awareness, do not need to be distinguished but can instead be subsumed under the umbrella term of minimal phenomenal experience (MPE)^8,24,25,33,37^. In contrast, the second perspective posits that these two experiences are distinct and should not be conflated; only the second type should be referred to as non-dual awareness^5,6,30,33,36^. We here follow this second definition of non-dual awareness as distinct from, and only in part overlapping with, MPEs^8,25,33,36^ (Fig. 1A). This viewpoint places non-dual awareness along an implicit–explicit gradient: the implicit pole means that non-dual awareness is always already present, even during sleep or normal wakefulness, but remains hidden, tacit and thus opaque within our actual experience. Although the explicit pole of the implicit-explicit gradient refers to the explicit occurrence of non-dual awareness within the actual experience of our consciousness^30^.

Following this conceptual framework, we define and operationalize non-duality based by three factors: 1) the non-duality cluster of the Meditation Depth Questionnaire (MEDEQ)^38^, which we use to assess the experience of non-duality, as one of the several dimensions captured by this scale^39–43^ (see methods); 2) the Isha Yoga tradition^39,44–47^ (see supplementary note for more information on the tradition and practices), where non-dual awareness is conceived as the most basic and fundamental background layer of our consciousness^6,11,32^, as distinguished from a more cognitive view featured by meta-awareness and meta-representation as forms of higher-order cognition^30,35^ ((Figs. 1A, 3)) by the degree of difference in intrinsic neural timescales duration (estimated by ACW) between internal (breath-watching meditation) and external (visual oddball task) attention (see below for details), such that the lack of internal-external difference on the neuronal level is supposed to relate to the lack of internal-external difference in the experience of non-duality (as measured by the MEDEQ).

What about the neuronal correlates of non-dual awareness? Preliminary evidence suggests that the central precuneus, a core region of the default mode network (DMN), and its interactions with prefrontal regions may play a key role in mediating non-dual awareness^5,6^. Research also shows neural synchronization of internal and external processing during advanced meditation with topographic re-organization of activity in the DMN (related to internal processing^3,48–50^) and the central executive network (CEN) (related to external processing^51–53^) in relation to the experience of non-duality^11^. While research has primarily focused on the spatial organization of brain networks associated with non-dual awareness, how this state manifests in the temporal dynamics of neural activity remains to be explored.

Recent evidence suggests that the brain’s neural activity is characterized by intrinsic neural timescales (INTs)^54–61^. INTs allow for the processing of stimuli characterized by their different temporal signatures, i.e., different sets of timescales. Further, research shows that these INTs provide temporal receptive windows (analogous to spatial receptive fields in the visual cortex^62–65^) which allow segregating and integrating both internal and external inputs of varying durations^57,66–73^. These INTs are measured by the auto-correlation function (ACF), which is a function “*that correlates a signal with copies of itself that are temporally shifted with a series of lags*^55,56,59,70^.” It is typical in research to report the autocorrelation window (ACW), defined as “*the length of time at the moment when the ACF decays to 50% of its maximum value (ACW-50)*^70,74–76^.”

INTs have been involved in both internal and external tasks^1,54–56,75,77,78^. Internal tasks like mind wandering and self-reference have been associated with longer durations or timescales^55,56,75,78^, while external tasks with visual or auditory stimuli exhibit shorter duration or timescales^55,79^. INTs have been shown to play a role in perception, behavior, cognition, consciousness, and a sense of self^54–56,59,74,75,77,78^. Further, INTs have been shown to be sensitive to distinct attentional processes. For instance, meditative practices with broader attentional engagement, such as Shoonya, exhibit longer timescales, whereas practices with narrower attentional engagement, such as mantra meditation, show shorter timescales^80^. This leaves open the role of INTs in non-dual awareness, which, as indicated above, can be observed across different meditative practices^5,6,10,11,18,20,21,24–28,81^. Addressing this is the primary goal of our study.

Our study has three key aims: (1) characterizing non-duality at the subjective-experiential, e.g., psychological level using standard psychological scales, (2) identifying its neural markers and specifically its timescales by measuring EEG-based ACW during both internal (breath-watching meditation) and external (visual oddball) tasks, and (3) investigating the relationship between subjective experience (scales) and intrinsic neural timescales (ACW) of non-duality. Besides the main data set, we also include a replication data set (see Supplementary Fig. 4) where the main results are replicated.

Our first specific aim was to investigate non-duality at the psychological level among study participants. Based on previous literature^5,6,10,11,18,20,21,24–28^, we hypothesized that advanced meditators would exhibit higher levels of the subjectively experienced state of non-duality and a fundamental tendency to not align themselves with either internal or external realms, i.e., non-attachment^82–84^. Moreover, we hypothesized that non-attachment, as a trait feature, would correlate with state-level non-duality, reflecting the experienced state during meditation.

Our second aim was to examine non-duality at the neural level in advanced meditators, novice meditators and controls during breath-watching (internal attention) and visual oddball tasks (external attention). We hypothesized that advanced meditators would experience non-dual awareness and thus exhibit similar durations in their timescales during both the internally directed breath-watching meditation and the externally directed visual oddball task. Because non-dual awareness is non-preferential to content and thus ultimately devoid of the difference of internal (breath watch) and external (visual oddball) phenomenal contents^8,30^, we expect no neuronal differentiation in the brain’s INT between internal and external attention conditions in advanced meditators.

Our third specific aim was to address whether these neural measures of non-duality, i.e., internal-external ACW difference, relate to, or correlate with subjective measures of non-duality, i.e., the factor non-duality of the MEDEQ. We hypothesized that lesser differences in ACW between internal-attention breath-watching and external-attention cognitive task would relate to higher degrees in the experience of non-duality at the subjective experiential, e.g., psychological level (Fig. 1 B).

In summary, our study introduces a psychological and neural perspective on the experience of internal-external non-duality in advanced meditators. First, we show that advanced meditators exhibit greater experience of non-duality than control subjects during breath-watching meditation. Next, we demonstrate neural differences in ACW duration between internal attention (breath-watching meditation) and external attention (a cognitive task) in novice meditators and controls, indicating neuro-temporal duality. In contrast, advanced meditators no longer show these differences; instead, they exhibit greater similarity in ACW duration between internal and external tasks, suggesting neuro-temporal non-duality. Strongly supported by these and our various correlation and regression findings, we propose that the increased similarity in the brain’s timescales across internal and external tasks, i.e., their non-duality on the neural level, serves as a neural marker of non-duality on the psychological level in advanced meditators. Thus, the duration of the timescales may be shared by both neural and mental/experiential levels, e.g., brain and mind as their “common currency^1,54,58^.”

## Results

### Psychological features of non-duality

#### Meditation depth during breath watching

**Fig. 2:**
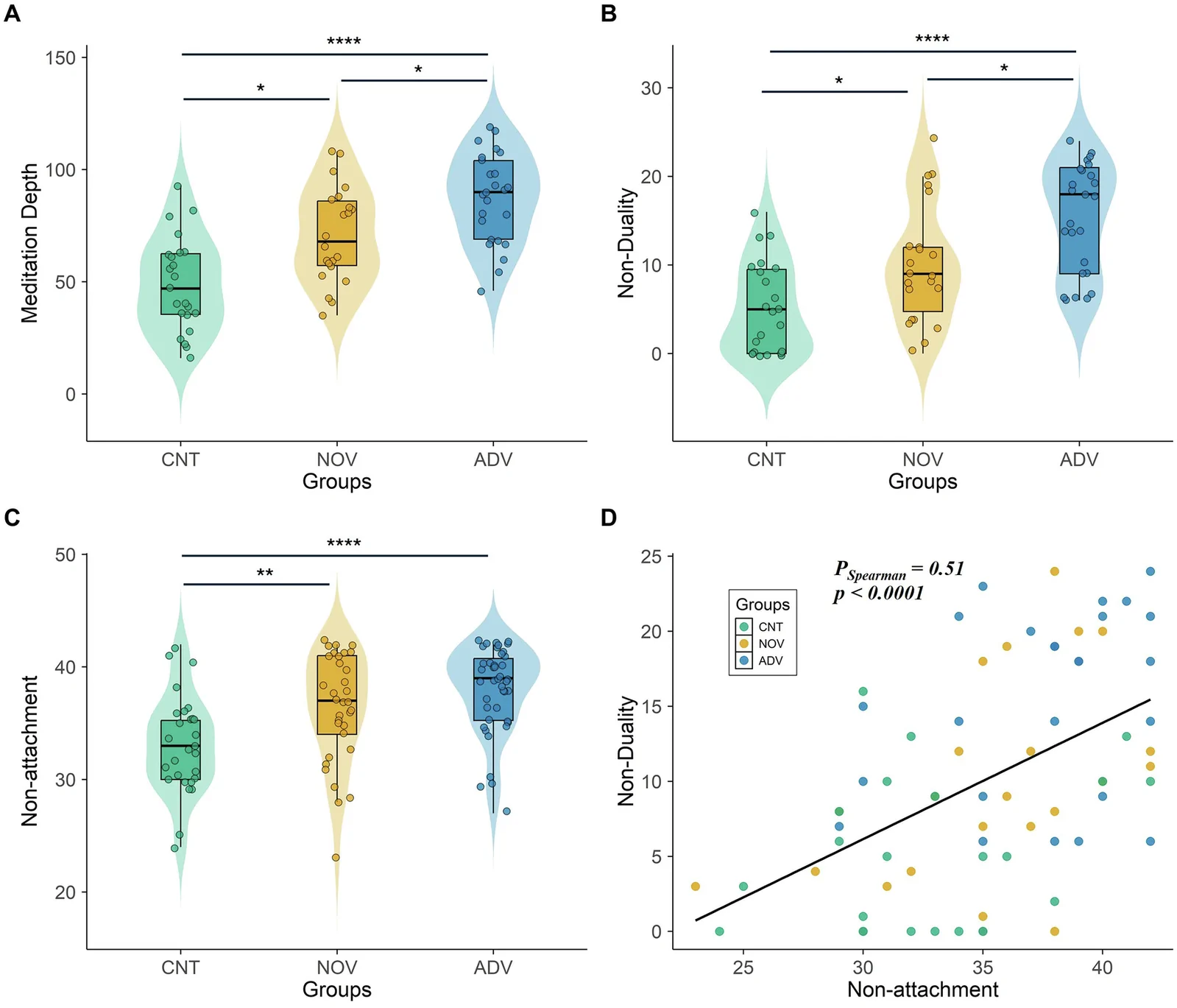
Psychological features of meditation depth and non-duality. **A** Box–violin plots depicting group differences in the meditation depth questionnaire (MEDEQ) among control (CNT), novice (NOV), and advanced (ADV) meditators. Advanced meditators (blue) showed the highest MEDEQ scores, followed by novice meditators (orange) and control participants (teal) (*χ*^2^_Kruskal–Wallis_(2) = 25.42, *p* < 0.0001, *ϵ*^2^_ordinal_ = 0.37, CI_95%_[0.25, 1.00], *n* = 70; CNT = 23, NOV = 22, ADV = 25). **B** Box-violin plots depicting group differences in non-duality, demonstrating increased non-dual experiences in advanced meditators (*χ*^2^_Kruskal–Wallis_(2) = 22.94, *p* < 0.0001, *ϵ*^2^_ordinal_ = 0.33, CI_95%_[0.21, 1.00], *n* = 70; CNT = 23, NOV = 22, ADV = 25). **C** Box-violin plots showing non-attachment differences across groups, with higher scores in meditators (*χ*^2^_Kruskal–Wallis_(2) = 18.42, *p* < 0.0001, *ϵ*^2^_ordinal_ = 0.18, CI_95%_[0.10, 1.00], *n* = 103; CNT = 28, NOV = 33, ADV = 42). **D** Scatterplot illustrating the positive relationship between non-duality and non-attachment across groups (*Ρ*_Spearman_ = 0.51, *p* < 0.0001, *n* = 70). The boxplot center line indicates the median, the boxes represent the interquartile range (IQR), and the whiskers extend to 1.5 × IQR. Significance levels: *p < 0.05, **p < 0.01, ****p < 0.0001.

To assess participant’ engagement and depth of absorption during breath-watching meditation, we analyzed group differences in MEDEQ global scores. As illustrated in Fig. 2A, the results show a significant difference in meditation depth (*χ*^2^_Kruskal–Wallis_(2) = 25.42, *p* < 0.0001, *ϵ*^2^_ordinal_ = 0.37) between the three groups. Advanced meditators exhibited the highest levels of MEDEQ, followed by novices, with controls reporting the lowest levels. The effect sizes were large. Post-hoc comparisons showed significant differences between advanced meditators and controls (*p*_Holm-adj_ < 0.0001), between advanced and novice meditators (*p*_Holm-adj_ = 0.03), and between novices and controls (*p*_Holm-adj_ = 0.01). As expected, we found a significant positive correlation between lifetime hours of meditation and MEDEQ (*Ρ*_*Spearman*_ = 0.60, *p* < 0.0001) (Supplementary Fig. 1A).

Together, these findings support and validate that the advanced meditators were indeed experienced practitioners, based on their lifetime meditation hours and their MEDEQ scores during breath-watching meditation. Correspondingly, lifetime hours and meditation depth correlated with each other. Hence, our advanced meditators are truly advanced in both objective (lifetime hours) and subjective (MEDEQ) terms, distinguishing them from novices, who, in turn, were different from the controls. This further validates our measure of meditation depth with regarding to the breath-watching meditation.

#### State features—non-duality during meditation in advanced meditators

Non-duality is a state of experience where subjects no longer differentiate between their internal reality (e.g., thoughts and the self) and external reality (e.g., events and objects in the environment). By assessing the degree of non-duality during breath-watching meditation using the non-duality cluster of the MEDEQ administered immediately after the session, we compared the three groups in terms of the degree of non-dual experience during meditation. We supposed the experience of non-duality to be higher in the advanced meditators than in the other two groups. This hypothesis was confirmed: as illustrated in Fig. 2B, the results show a significant difference in the experience of non-duality during breath-watching (*χ*^2^_Kruskal–Wallis_(2) = 22.94, *p* < 0.0001, *ϵ*^2^_ordinal_ = 0.33) between the three groups. Advanced meditators exhibited the highest levels of non-duality, followed by novices, with controls reporting the lowest levels. The effect sizes were large. Post-hoc comparisons for non-duality showed significant differences between advanced meditators and controls (*p*_Holm-adj_ < 0.0001), between advanced and novice meditators (*p*_Holm-adj_ = 0.04), and between novices and controls (*p*_Holm-adj_ = 0.04). Further, we found a positive correlation between non-duality and lifetime hours of meditation (*Ρ*_*Spearman*_ = 0.57, *p* < 0.0001) (Supplementary Fig. 1B).

Together, our results show that advanced meditators experience high degrees of non-duality, significantly greater than those of novices and controls. Moreover, the experience of non-duality correlated with the lifetime hours, further supporting the validity of our non-duality measure. Accordingly, non-duality emerges as a key feature of breath-watching meditation, particularly in advanced meditators and, to a lesser extent, in novices and controls.

#### Trait features—non-attachment and its relation to non-duality

We measured the experience of non-duality directly in relation to the meditative state during breath-watching, which was also associated with lifetime meditation hours. This raises the question of whether non-duality is not only a state-dependent feature of meditation but also a more enduring trait of advanced practitioners. One key feature of the non-dual state is the individual’s ability to refrain from identifying with, aligning with, or attaching to any form of content, including internal thoughts or feelings as well as external environmental stimuli. This ability can be measured with the non-attachment scale (NAS-7), which quantifies the individual’s capacity to not attach or identify with any particular content of experience. Results (Fig. 2C) show a significant difference between the groups in non-attachment (*χ*^2^_Kruskal–Wallis_(2) = 18.42, *p* < 0.0001, *ϵ*^2^_ordinal_ = 0.18). The effect sizes were large. Advanced meditators reported the highest scores for non-attachment. Post-hoc comparisons for non-attachment showed significant differences between advanced meditators and controls (*p*_Holm-adj_ < 0.0001) and between novices and controls (*p*_Holm-adj_ < 0.01). We also found a significant positive correlation between non-attachment and lifetime hours of meditation (*Ρ*_*Spearman*_ = 0.44, *p* < 0.0001) (Supplementary Fig. 1C). Finally, as expected, we found a significant positive correlation between NAS-7 and non-duality (*Ρ*_*Spearman*_ = 0.51, *p* < 0.0001) (Fig. 2D), indicating that higher levels of non-attachment were associated with greater degrees of non-duality. Accordingly, our results show that the subjects’ capacity for non-attachment relates to their non-duality during meditation.

Together, our findings demonstrate that advanced meditators also exhibit greater trait capacity for non-attachment to both internal and external mental content. Furthermore, the strong correlation between non-attachment and non-duality supports the idea that non-attachment is a key characteristic of non-duality, with higher levels of non-attachment (as a trait) associated with greater degrees of non-duality (as a state). Hence, our data strongly support the central role of non-duality and non-attachment as core features of the breath-watching experience in advanced meditators, distinguishing them from both novices and controls.

### Neuronal features of non-duality

#### Neuro-temporal duality–longer ACW during internal (breath watch) than external (visual oddball) attention across all subjects

**Fig. 3:**
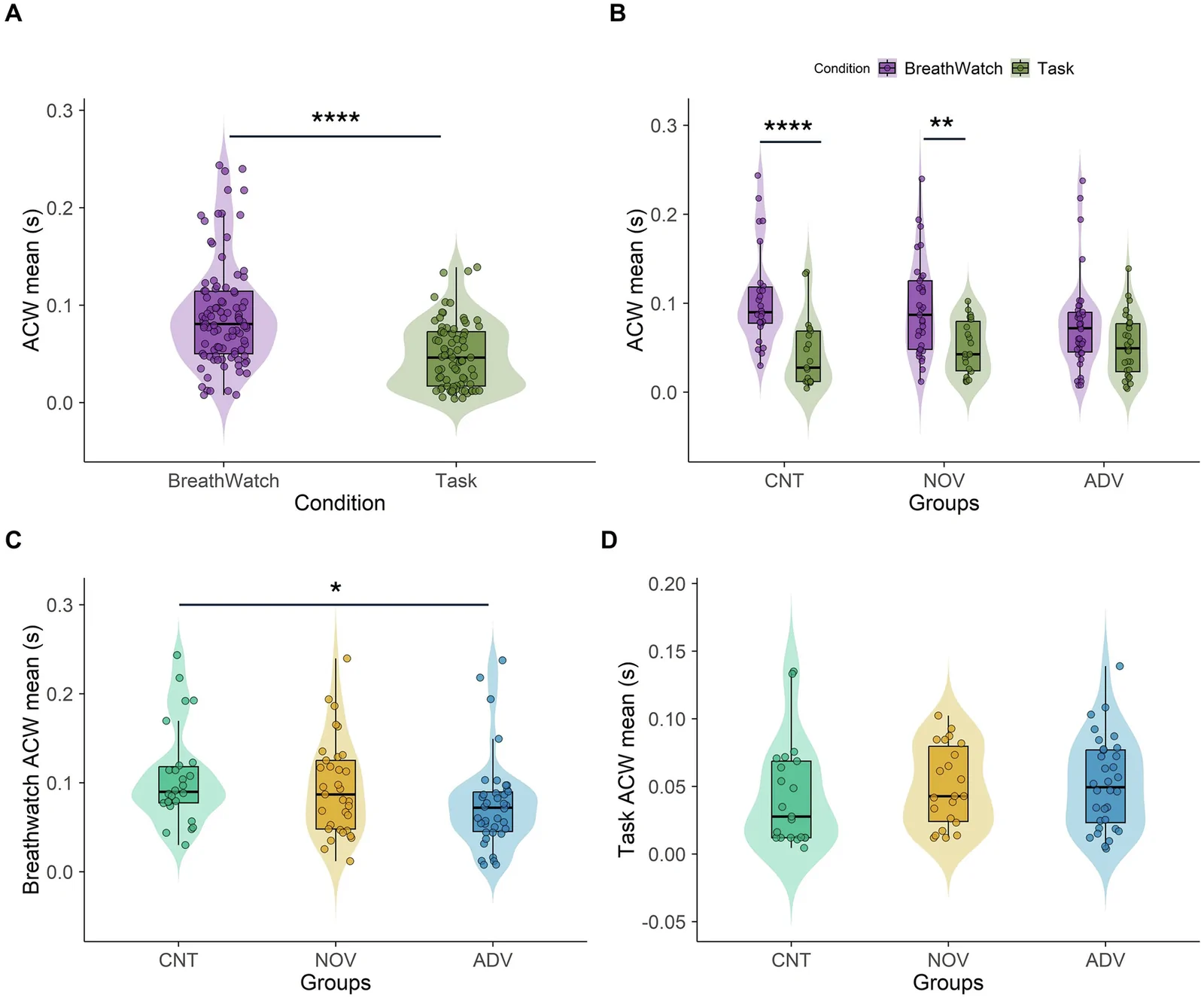
Duration of auto-correlation window (ACW) during breath-watching meditation and cognitive task. **A** Difference in auto-correlation window (ACW) mean between internal-attention breath-watching meditation and external-attention cognitive task (all groups combined). ACW becomes shorter and narrower during the external task, while it becomes longer and wider during the internal-attention breath-watching (*t*(163.52) = 6.09, *p* < 0.0001, *g*_Hedges_ = 0.91, CI_95%_ [0.60, 1.21], *n* = 172; BreathWatch = 97, Task = 75)). **B** Group-wise ACW mean for both conditions, where CNT and NOV groups exhibit significantly higher ACW mean in the BreathWatch condition compared to the Task condition (CNT: *t*(70) = 4.804, *p* = 0.0001, *n* = 20; NOV: *t*(70) = 3.782, *p* = 0.0042, *n* = 22), whereas ADV do not show any significant difference between these two conditions (*t*(70) = 1.770, *p* = 0.4918, *n* = 31). **C** Comparison of ACW mean during BreathWatch across groups (CNT, NOV, ADV), showing a significantly lower ACW in the advanced meditators (*χ*^2^_Kruskal–Wallis_ (2) = 6.59, *p* = 0.04, *ϵ*^2^_ordinal_ = 0.07, CI_95%_ [0.00981, 1.00], *n* = 97; CNT = 26, NOV = 33, ADV = 38)). **D** No significant differences in ACW mean between groups during the Task condition (*χ*^2^_Kruskal–Wallis_ (2) = 2.23, *p* = 0.33, *ϵ*^2^_ordinal_ = 0.03, CI_95%_ [0.00475, 1.00], *n* = 75; CNT = 21, NOV = 22, ADV = 32). The boxplot center line indicates the median, the boxes represent the interquartile range (IQR), and the whiskers extend to 1.5 × IQR. Significance levels: *p < 0.05, **p < 0.01, ****p < 0.0001. CNT controls, NOV novice meditators, ADV advanced meditators.

Non-duality refers to the absence of a distinction between the internal self and the external environment, thereby overcoming their duality in our common experience^6,8^. This concept can be operationalized at the neural processing level by comparing the difference in EEG-based ACW duration between an internal-attention breath-watching meditation and an external-attention cognitive task. We hypothesized that the difference in ACW duration between internal and external attention tasks would indicate the level of dual (‘neuro-temporal duality’) or non-dual (‘neuro-temporal non-duality’) processing at the neural level. To test this, we first combined all the groups. We examined the differences in ACW mean between breath-watching and cognitive task conditions using an independent sample t-test, thus assessing the overall neural difference between internal (breath-watching meditation) and external (visual oddball task) processing (Fig. 3A). Our results show a statistically significant difference, with a very large effect size, between the internal-oriented breath-watching and the external-oriented cognitive task (*t*(163.52) = 6.09, *p* < 0.0001, *g*_*Hedges*_ = 0.91). In other words, the ACW mean becomes shorter and thus narrower (in its temporal cross-correlation between distant time points) during an externally oriented condition (visual oddball cognitive task). In contrast, it is longer and thus wider during an internally-oriented condition (breath-watching meditation).

Together, these results confirm our hypothesis that the duality of the internal (breath-watching) and the external (cognitive task) on the psychological level is also manifest on the neural level in terms of different processing durations, i.e., timescales, as indicated by longer ACW during the internal breath-watching and shorter ACW during the external cognitive task. This further suggests that the difference in the ACW between both breath-watching and the cognitive task can serve as a neural proxy for duality vs. non-duality. Consequently, one may want to speak of ‘neuro-temporal duality’ featuring the different processing durations—i.e., timescales—of internally (breath-watching) and externally (visual oddball) directed attention.

#### Neuro-temporal non-duality I—no difference between internal and external ACW in advanced meditators

Next, we assessed how meditation experience influences this ACW-based neuro-temporal duality in processing external and internal attention conditions. To this end, a mixed ANOVA was conducted to examine the effects of meditation experience (group: advanced, novice, control) and condition (breath-watching, visual oddball) on the dependent variable, ACW means (Supplementary Table 1). The analysis revealed a significant main effect of condition, *F*(1, 70) = 37.84, *p* < 0.0001, *η*²_p_ = 0.351 (large effect size), indicating that the ACW mean differed significantly between the breath-watching and cognitive task conditions. However, the main effect of the group was not significant, *F*(2, 70) = 0.74, *p* = 0.48, *η*²_p_ = 0.021 (small effect size), suggesting that lifetime meditation hours and exposure alone are unlikely to account for differences in internal and external ACW. In contrast, there was a significant interaction effect between group and condition, *F*(2, 70) = 3.76, *p* = 0.028, *η*²_p_ = 0.097 (medium effect size), indicating that the effect of condition varied across groups. This finding emphasizes the relationship between ACW and the actual state (i.e., breath-watching vs. visual oddball cognitive task) rather than a purely trait-based effect of meditation experience (i.e., group independent of condition).

Post-hoc pairwise comparisons (Supplementary Table 2) were conducted to assess differences in ACW mean between breath-watching meditation and the visual oddball cognitive task within each group of meditators (advanced, novice, control) (Fig. 3B). This difference was computed to test for the degree of duality (large ACW differences between breath-watching and visual oddball cognitive task) vs. non-duality (small ACW differences between breath-watching and visual oddball cognitive task) on the neuronal level. In advanced meditators, no significant difference in ACW mean was observed between breath-watching and the visual oddball cognitive task (*p* = 0.4918), indicating that their neural processing duration (i.e., ACW mean) remained similar across internal (breath-watching) and external (visual oddball cognitive task) domains. In contrast, novice meditators showed a significant ACW difference between internal and external tasks (*p* = 0.0042), suggesting distinct neural processing durations for internal and external tasks. Similarly, the control group exhibited a highly significant difference (*p* = 0.0001) (Fig. 3B), suggesting a strong condition effect in this group. All *p*-values were adjusted using Holm’s method for multiple comparisons. Together, these results demonstrate a more non-dual neural expression of ACW in advanced meditators (i.e., no significant difference between internal breath-watching and the external cognitive task). In contrast, novices and controls exhibited a more dual-processing pattern (i.e., a significant ACW difference between the two conditions).

Next, given that the ACW is known to reflect the balance of temporal integration (longer ACW) and segregation (shorter ACW) during input processing^56,78^, as entailed by both breath-watching and the visual oddball cognitive task (see the introduction for details on that), we examined group differences in ACW values for each condition separately. We observed that the ACW during breath-watching was significantly shorter in the advanced meditators than in both controls and novices (*χ*^2^_Kruskal–Wallis_(2) = 6.59, *p* = 0.04, *ϵ*^2^_ordinal_ = 0.07) (Fig. 3C). In contrast, there was no significant difference in the ACW of the externally-oriented visual oddball cognitive task between the three groups (*χ*^2^_Kruskal–Wallis_(2) = 2.23, *p* = 0.33, ϵ^2^_ordinal_ = 0.03) (Fig. 3D). Next, we also investigated behavioral measures of the visual oddball task. Response times did not significantly differ across the three groups (Supplementary Fig. 2A). In contrast, response accuracy showed significant group differences (Supplementary Fig. 2B), with advanced meditators demonstrating the highest accuracy compared to the other groups.

Together, we show that advanced meditators exhibit significantly less or lower ACW difference between internal (breath-watching) and external (oddball) conditions compared to both controls and novices. This effect is primarily driven by a shortening of the ACW during the internal condition (breath-watching). At the same time, it exhibits the same duration among all three groups during the external task. Accordingly, unlike the other two groups, the ACW during the internal condition approximates and becomes similar to the ACW during the external condition in advanced meditators. This indicates that both internal and external contents are processed similarly at the neural level, that is, they are processed with similar duration in their timescales. Therefore, ACW duration in advanced meditators no longer differs between internal and external processing, suggesting a higher degree of non-dual processing at the neural level—what we refer to as ‘*neuro-temporal non-duality*.’ Conversely, controls and novices exhibit greater differentiation in ACW duration between internal and external tasks, indicating more dual processing in the temporal dynamics of their neural activity, thus reflecting a higher degree of neuro-temporal duality.

#### Neuro-temporal non-duality II—Is it related to the brain’s spontaneous activity?

We have so far demonstrated that differences in the psychological state—dual vs. non-dual—are present in an analogous and corresponding way on the neural level of the brain’s processing duration, as measured by the ACW. Controls and novices exhibited different lengths in their ACW during internal and external tasks, reflecting a stronger degree of neuro-temporal duality. Conversely, advanced meditators no longer showed such an ACW difference, reflecting a tendency towards neuro-temporal non-duality. This raises the question of where and how neuro-temporal differences in advanced meditators are coming from during the internal (breath-watching) task.

**Fig. 4:**
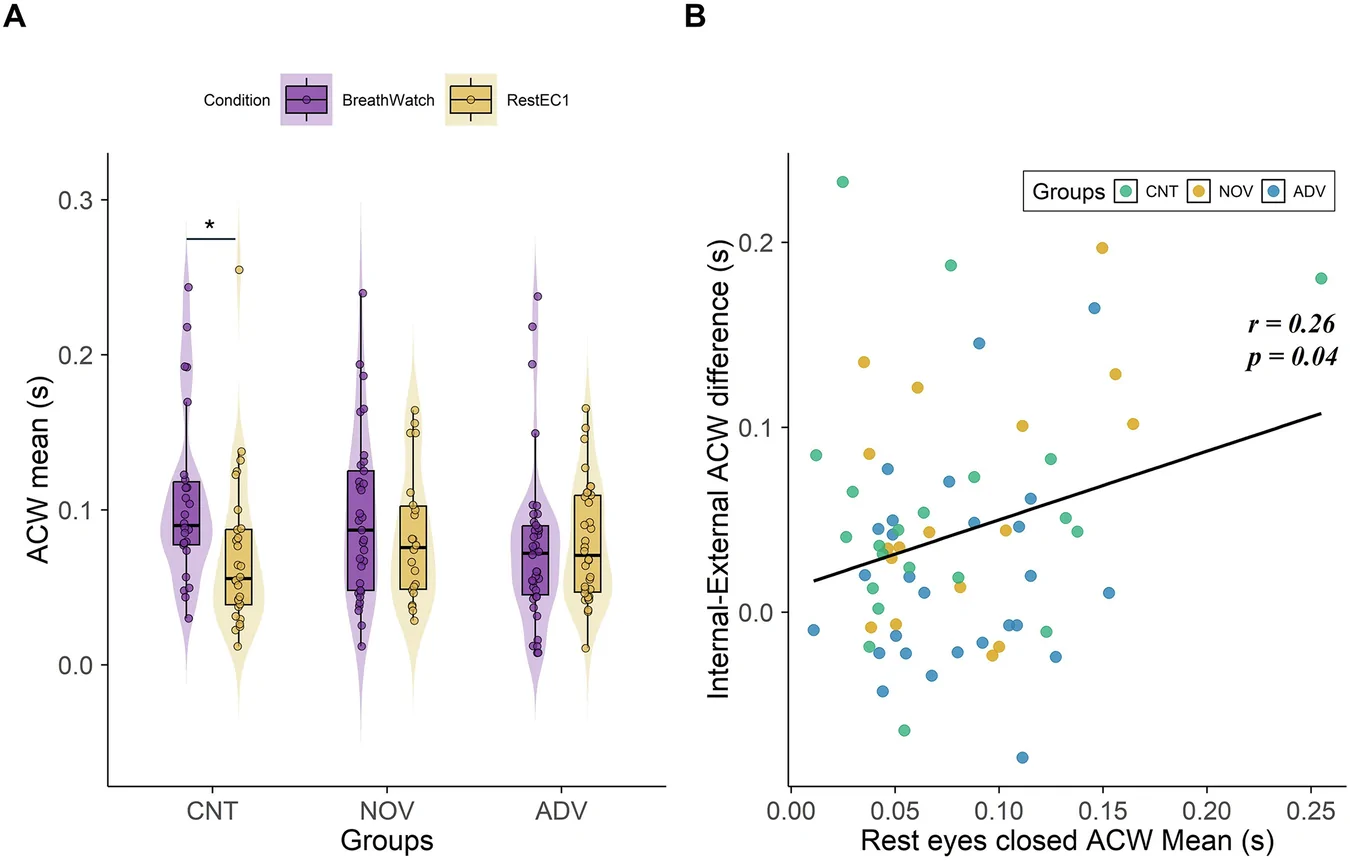
Relationship between the brain’s spontaneous activity and the internal-external ACW difference. **A** The difference in ACW mean between internal-attention breath-watching meditation and eyes closed resting state. There is no significant difference between breath-watching and rest in novice (*t*(76) = 2.639, *p* = 0.1003, *n* = 22) and advanced (*t*(76) = 0.053, *p* = 1.0000, *n* = 31) meditators. In contrast, controls show significant differences (*t*(76) = 3.477, *p* = 0.0105, *n* = 26). **B** Scatter plot illustrating the significant positive correlation between Rest eyes closed ACW mean and internal-external ACW difference (*r* = 0.26, *p* = 0.04, *n* = 65_pairs,_ CNT = 21, NOV = 17, ADV = 27). The boxplot center line indicates the median, the boxes represent the interquartile range (IQR), and the whiskers extend to 1.5 × IQR. Significance levels: *p < 0.05. CNT controls, NOV novice meditators, ADV advanced meditators.

To explore this, we investigated spontaneous neural activity during the resting state, which has been shown in previous papers to predict task-related differences in ACW^56,79^. We first compared the ACW in the resting state among the three groups, finding no significant differences. Next, we compared the differences between the resting state and the internal task (breath-watching) across the three groups. A mixed ANOVA was conducted to examine the effects of meditation experience (Group: advanced, novice, control) and condition (breath-watching, resting state) on the dependent variable, ACW mean (Supplementary Table 3) (Fig. 4A). The analysis revealed a significant main effect of condition, *F*(1, 76) = 13.5393, *p* < 0.001, *η*²_p_ = 0.151 (large effect size), indicating that the ACW mean differed significantly between the breath-watching and resting state conditions. However, the group’s main effect was insignificant, *F*(2, 76) = 0.9908, *p* = 0.3760, *η*²_p_ = 0.025 (small effect size). Conversely, there was a significant interaction between group and condition, *F*(2, 76) = 3.6774, *p* = 0.0298, *η*²_p_ = 0.088 (medium effect size), indicating that the effect of condition varied across groups.

Further, post-hoc pairwise comparisons (Supplementary Table 4) were conducted to assess the ACW differences between breath-watching meditation and resting state within each group of meditators (Fig. 4A). In the advanced meditators, no significant difference in ACW mean was observed between breath-watching and resting state (*p* = 1.0000). Similarly, in the novice meditators, no significant difference was found (*p* = 0.1003). However, in the control group, a significant difference was observed (*p* = 0.0105), suggesting a difference in ACW mean between breath-watching and resting state.

Finally, we found a significant positive correlation of the resting state ACW with the internal (breath-watching)-external (visual oddball) task ACW differences (*r* = 0.26, *p* = 0.04) (Fig. 4B) across all groups. This suggests that the degree of duality (or non-duality) on the neuronal level (difference in ACW between internal and external) relates to the duration of the ACW in the resting state: longer duration in the resting state ACW is associated with larger internal-external ACW differences, entailing a higher degree of dual internal-external processing. In contrast, shorter ACW in the resting state leads to lower internal-external ACW differences, reflecting a more non-dual internal-external processing

We also conducted the same analysis for the resting state-external task (visual oddball) differences among the three groups, which revealed a significant main effect of condition but no significant effects of group or group × condition interactions (Supplementary Table 5, Supplementary Fig. 3). Post-hoc comparisons indicated a marginally significant difference between the resting state and the external task (oddball) for controls (*p* = 0.08) (Supplementary Fig. 3). In contrast, no significant differences were observed for novices or advanced meditators.

Overall, these results suggest that advanced meditators do not show significant ACW differences across the division of all three states, that is, intrinsic (resting state), external (visual oddball task), and internal (breath-watching meditation) states; that is, for internal breath-watching vs. external task (oddball), resting state vs. breath-watching, and resting state vs. external task (oddball). In the advanced meditators, ACW duration remains consistent across all three states, whereas this is not the case in the other two groups. Hence, these findings further support the idea of neuro-temporal non-duality as a core feature of a non-dual internal-external neural processing across all rest and task states in advanced meditators. In contrast, controls exhibit significant differences across all conditions, supporting that their neural processing conforms to neuro-temporal duality.

#### Neuro-temporal non-duality in the replication dataset

In an independent dataset of 43 expert meditators (see Methods), we replicated the neuro-temporal non-duality findings observed in our primary sample. There were no significant differences in ACW duration between breath-watching meditation, rest, and the visual oddball task (F(2, 64.68) = 0.06, *p* = 0.95; Supplementary Fig. 4). This further supports the validity of our results.

#### Relationship between ACW and frequency spectrum

We have so far shown that advanced meditators exhibit significantly less or lower ACW difference between eyes-closed internal (breath-watching) and eyes-open external (oddball) conditions compared to both controls and novices. Are these changes related to differential eye-state alpha effects between the groups?

To address this, we conducted analysis of alpha power across groups and conditions (Supplementary Fig. 5). A mixed-design ANOVA (Supplementary Table 6) revealed no significant Group × Condition interaction in alpha power (F(2,48) = 0.24, *p* = 0.788), indicating that task-related modulation of alpha activity did not differ across groups. If the observed ACW effects were primarily driven by eye-state-related alpha changes, one would expect corresponding group differences in alpha modulation. To further test whether these effects were attributable to alpha power, we conducted a linear mixed-effects model analysis (Supplementary Table 7) including alpha power as a covariate: ACW ~ Group × Condition + Alpha + (1 | Subject). The Group × Condition interaction remained significant after controlling for alpha power (F(2, 87.68) = 3.21, *p* = 0.045), indicating that the observed ACW effects are not fully explained by alpha-band activity. Taken together, these findings suggest that while alpha power is influenced by eye state, the group differences in ACW cannot be reduced to eye-state-related alpha modulation alone and likely reflect additional aspects of neural temporal dynamics.

Additionally, to address the relationship between ACW and the overall frequency spectrum, and to study if our ACW results hold true after controlling for spectral composition, we conducted power spectral analysis across delta, theta, alpha, beta1, beta2, and gamma bands for all groups (advanced, novice, and controls) and conditions (breath-watching and cognitive task) (Supplementary Fig. 6). A mixed-design ANOVA (Supplementary Table 8) with Group (Advanced meditators, Novice meditators, Controls) as a between-subject factor and Condition (Breath-watching, Cognitive Task) and Frequency Band (Delta, Theta, Alpha, Beta1, Beta2, and Gamma) as within-subject factors revealed a trend-level Condition × Band interaction, F(1.37, 98.73) = 3.08, *p* = 0.069, suggesting modest task-related spectral shifts across frequency bands. Importantly, the Group × Condition × Band interaction was not significant, F(2.74, 98.73) = 0.60, *p* = 0.602, indicating no evidence for differential spectral modulation between groups. If the observed ACW differences between our three groups were primarily driven by spectral composition, one would expect corresponding group differences in task-related spectral modulation. However, such differences were not observed.

To further test whether ACW effects were reducible to spectral composition, we conducted a linear mixed-effects model analysis (Supplementary Table 9) including band-limited power (delta, theta, alpha, beta1, beta2, gamma) as covariates:

ACW ~ Group × Condition + Delta + Theta + Alpha + Beta1 + Beta2 + Gamma + (1 | Subject)

The Group × Condition interaction in ACW remained significant after controlling for spectral power, F(2, 92.16) = 3.11, *p* = 0.049, indicating that the observed ACW differences are not fully explained by band-limited spectral power alone. Taken together, these findings suggest that while ACW is related to spectral composition, it captures aspects of neural temporal dynamics that are not fully reflected in conventional spectral measures. This is in line with other studies showing similar findings^79,85^. Importantly, this means that the group differences in ACW between advanced meditators and controls cannot be accounted for by their spectral composition, as this showed no corresponding group differences.

### Linking psychological duality/non-duality to neuro-temporal duality/non-duality

#### Correlation between internal-external ACW difference and non-duality

**Fig. 5:**
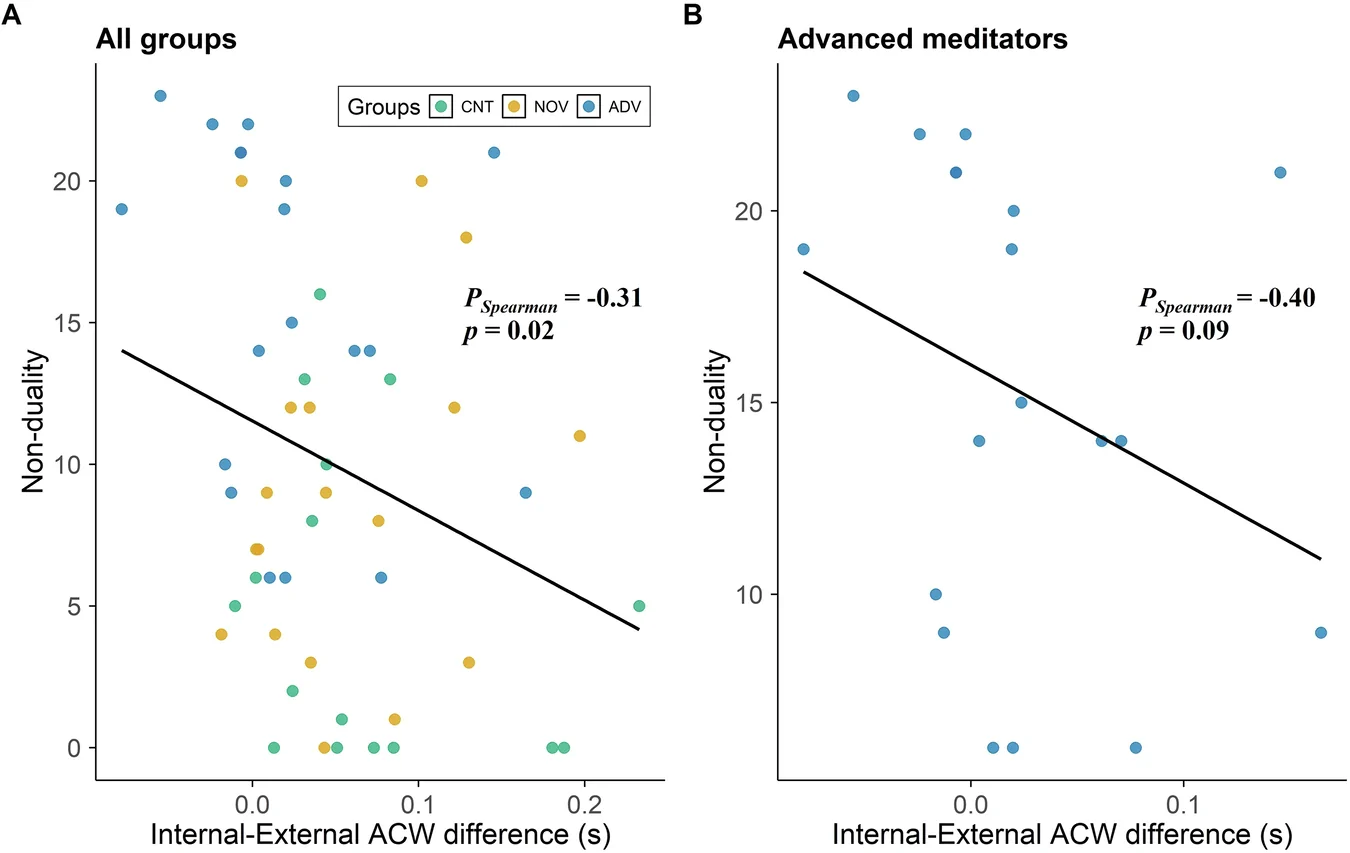
Correlation between internal-external ACW difference and non-duality. **A** Scatter plot illustrating the significant negative correlation between Internal-External ACW difference and non-duality in all groups combined (*S* = 32535.91, *p* = 0.02, *Ρ*_Spearman_ = −0.31, CI_95%_ [−0.54, −0.04], *n*_pairs_ = 53, CNT = 16, NOV = 18, ADV = 19). **B** Scatter plot depicting the negative correlation between the Internal-External ACW difference and non-duality in the advanced meditators (*S* = 1598.02, *p* = 0.09, *Ρ*_Spearman_ = −0.40, CI_95%_ [−0.73, 0.08], *n*_pairs_ = 19). CNT controls, NOV novice meditators, ADV advanced meditators.

How is the similarity of the internal-external ACW on the neural level related to the experience of the duality/non-duality on the psychological level? Our final step links ACW duration during internal breath-watching and external cognitive tasks to the psychological measures of non-duality. To explore this, we investigated the relationship between the internal-external ACW duration difference and the psychological scores of non-duality in all participants combined. Our results showed a significant negative correlation between internal-external ACW difference and non-duality (*Ρ*_Spearman_ = −0.31, *p* = 0.02) (Fig. 5A), indicating that a smaller internal-external ACW difference at the neural level is associated with a higher degree of non-duality at the psychological level (moderate effect size). To further examine this association, we analyzed the advanced meditator group, who are more likely to have experienced non-dual awareness. A similar negative correlation was observed (*Ρ*_Spearman_ = -0.40, *p* = 0.09) (Fig. 5B), showing a trend toward significance with a comparable moderate effect size. Further, advanced meditators were divided into high and low non-duality groups based on a median split of their non-duality scores. Those with higher non-duality reported smaller internal–external ACW differences (*p* = 0.09, rank-biserial effect size = –0.47) (Supplementary Fig. 7). Although the finding reached only trend-level significance, likely due to the limited sample size, the effect size was very large.

These findings suggest that the neural measure of non-duality—specifically, the internal-external ACW duration difference—relates to non-duality on the psychological level. As mentioned in the previous section, the smallest difference (or lack thereof) in ACW between the dual conditions (breath-watching vs. visual oddball task) was observed in the advanced meditators, while the largest difference was observed in the control group, with novices falling in between.

In summary, we show that smaller ACW differences between the dual processing of the internal (breath-watching) and external (visual oddball task) conditions relate to higher non-duality scores on the psychological level. This supports a direct relationship between neuro-temporal duality/non-duality in the brain and the subjective experience of duality/non-duality on the psychological level.

### Correlation between internal-external ACW difference and MEDEQ clusters

**Fig. 6:**
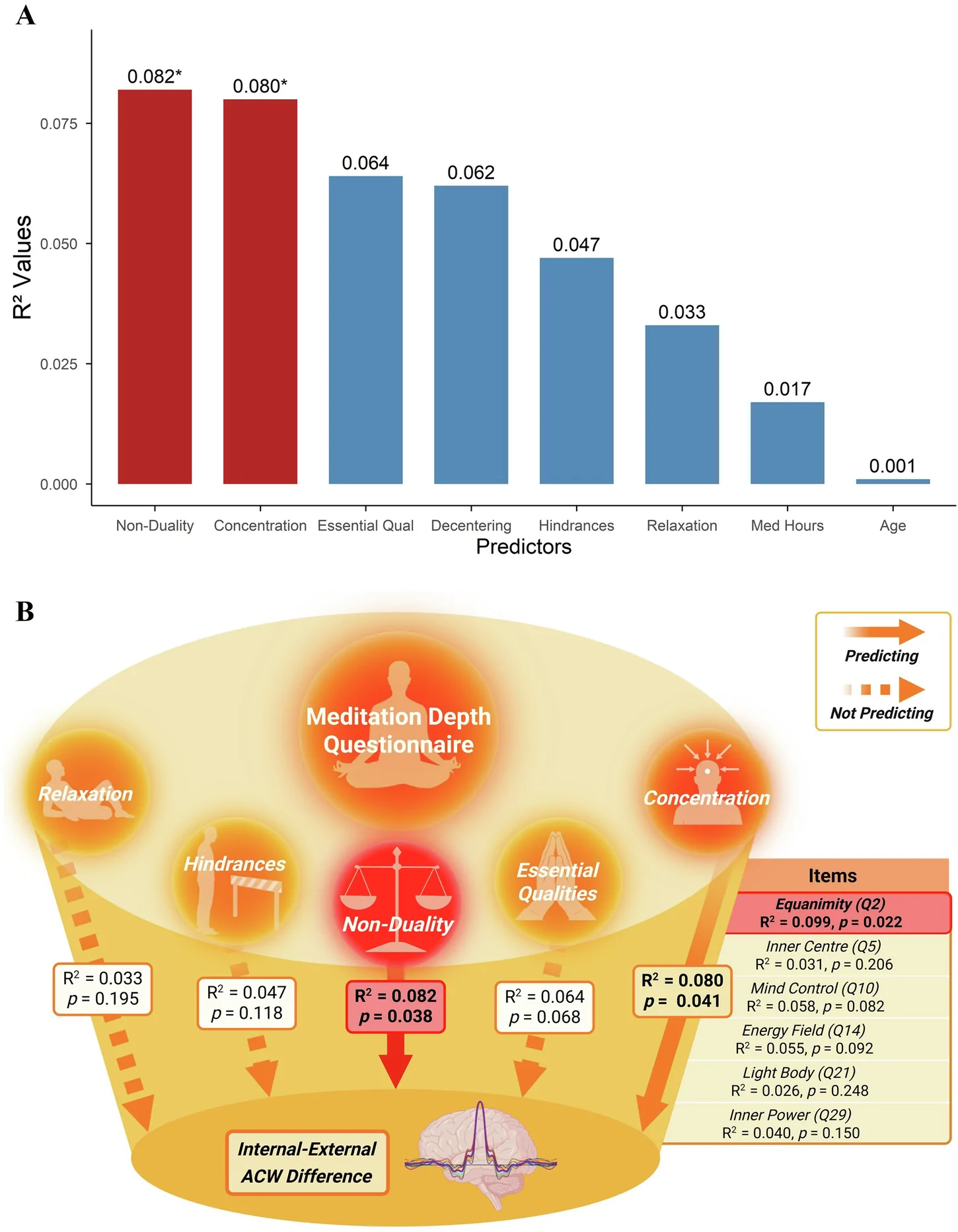
Correlation between the internal-external ACW difference and different meditation depth questionnaire clusters. **A** Independent linear regression analyses showing the variance in the internal–external ACW difference explained by different clusters of the Meditation Depth Questionnaire (hindrances, relaxation, concentration, essential qualities, and non-duality). Decentering (Q5, Q10), meditation lifetime hours, and age were also included as predictors. Non-duality (*R*² = 0.082, *p* = 0.038) emerged as the strongest predictor of the internal–external ACW difference, followed by the concentration cluster (*R*² = 0.080, *p* = 0.041). None of the other clusters was significant. **B** Schematic representation of associations between different clusters of the Meditation Depth Questionnaire and the internal–external ACW difference. Only non-duality and the concentration cluster showed significant effects; within the concentration cluster, equanimity was the only significant item (*p* < 0.05). Created in BioRender. Saketh, M. (2026) https://BioRender.com/tc5nmvq.

Given that the non-duality cluster of MEDEQ correlated with the internal–external ACW difference, we next examined how other MEDEQ clusters were associated with this measure. To address this, we performed different independent linear regression analyses to quantify the variance in the internal–external ACW difference explained by different clusters of the MEDEQ (hindrances, relaxation, concentration, essential qualities, and non-duality). We also included decentering as an additional predictor. Although decentering is not directly assessed by the questionnaire, two items (Question 5: I became aware of a center inside myself; Question 10: I experienced some control over my mind; I could observe my thoughts from a distance) were combined to represent this construct. Our results (Supplementary Table 10, Fig. 6A) showed that non-duality (*R*² = 0.082, *p* = 0.038) was the strongest predictor of the internal–external ACW difference. Notably, non-duality explained more variance than the combined contribution of the other clusters (hindrances + relaxation + concentration + essential qualities; *R*² = 0.074, *p* = 0.048). Decentering did not reach significance (*R*² = 0.062, *p* = 0.073), nor did meditation lifetime hours (*R*² = 0.017, *p* = 0.351) or age (*R*² = 0.0006, *p* = 0.862).

We next examined which other clusters, apart from non-duality, predicted the internal–external ACW difference (Supplementary Table 10 and Fig. 6A). Relaxation (*R*² = 0.033, *p* = 0.195), hindrances (*R*² = 0.047, *p* = 0.118), and essential qualities (*R*² = 0.064, *p* = 0.068) were not significant predictors. Only the concentration cluster showed a significant association (*R*² = 0.080, *p* = 0.041). To identify the specific contributors, we further analyzed individual items within this cluster and found that only equanimity (Q2) significantly predicted the internal–external ACW difference (Fig. 6B). Together, these findings indicate that the internal–external ACW difference is best accounted for by the psychological factor of non-duality and, within the concentration cluster, by equanimity. In contrast, other psychological clusters or dimensions of the MEDEQ did not explain the internal-external ACW difference. This suggests a direct relation of the internal-external ACW difference with specifically non-duality on the psychological level.

### Is non-duality better predicted by the internal-external ACW difference or by internal ACW?

So far, we have shown that the internal-external ACW difference is smallest in the advanced meditator group, primarily driven by a shortening of ACW during the internal-attention breath-watching condition. This raises a key question: does internal ACW alone account for non-duality, or is the difference between internal and external ACW that matters? To address this, we conducted three types of analyses: simple linear regression, hierarchical regression, and mediation analysis.

In the first analysis, we tested three separate linear regression models (Supplementary Table 11). Our findings revealed that only Model 1 (predictor: Internal–External ACW difference) significantly predicted the non-duality cluster of the MEDEQ (*R*² = 0.082, *p* = 0.033). Neither Model 2 (predictor: Internal ACW; *R*² =0.029, *p* = 0.214) nor Model 3 (predictor: External ACW; *R*² = 0.045, *p* = 0.120) reached statistical significance.

In the second analysis, we performed two hierarchical regression models. In the first model (Supplementary Fig. 8A) (Supplementary Table 12), Internal ACW was entered in Step 1 to predict non-duality, but this was not significant (F(1,51) = 1.55, R² = 0.029, *p* = 0.220). When the Internal–External ACW difference was added in Step 2, the explained variance increased, reaching trend significance (F(2,50) = 2.75, *R*² = 0.099, Δ*R*² = 0.070, *p* = 0.074). In the second hierarchical regression model (Supplementary Fig. 8B) (Supplementary Table 13), the order was reversed: Internal–External ACW difference was entered first (Step 1), which yielded a significant result (F(1, 51) = 4.54, *R*² = 0.082, *p* = 0.038). However, when Internal ACW was added in Step 2, the overall model became non-significant (F(2,50) = 2.75, *R*² = 0.099, Δ*R*² = 0.017, *p* = 0.074). Similar results were obtained when External ACW was used instead of Internal ACW (see Supplementary Tables 14 and 15, Supplementary Fig. 9). These results show that it is only the Internal-External ACW difference that is significantly related to non-duality.

Finally, mediation analyses further supported the importance of the Internal–External ACW difference over the Internal ACW in predicting non-duality. As shown in Supplementary Fig. 8C, ACW difference predicted non-duality through full mediation by meditation depth (*p* = 0.048). In contrast, Internal ACW did not predict non-duality (Supplementary Fig. 8D), even with mediation by other meditation depth clusters (*p* = 0.374). Further analyses revealed that the relationship between ACW difference and non-duality was specifically mediated by the concentration cluster (*p* = 0.045; Supplementary Fig. 10), whereas other clusters (Supplementary Fig. 11A–D) did not show significant effects. Consistent with earlier findings (Fig. 6 and Supplementary Fig. 8), equanimity (within the concentration cluster) emerged as the key mediator of the relationship between ACW difference and non-duality (*p* = 0.028) (Supplementary Fig. 8E).

Overall, these different results convergently demonstrate that the difference in ACW between internal and external attention conditions is the critical predictor of non-duality, rather than ACW during internal attention alone.

Taken together, our results demonstrate that the degree of similarity of internal-external tasks in their ACW duration in the three groups is related specifically to the experience of non-duality (rather than other dimensions or clusters of the MEDEQ). These findings further support the central role of the brain’s neural processing duration, i.e., ACW, during the dual vs. non-dual processing of external and internal stimuli, in modulating the psychological experience of non-duality during meditation.

## Discussion

Our research investigated the psychological and neural features of non-duality in advanced meditators, novice meditators, and controls. To probe the difference between internal and external attention, participants performed two tasks: breath-watching meditation (internal attention) and a visual oddball task (external attention). We conceptualized non-duality as a reduction in the distinction between both internal and external attention, examined this on both psychological (subjective experience of non-duality, captured by the non-duality cluster of the MEDEQ) and neural (similar ACW durations for the 2 tasks) levels.

**Fig. 7:**
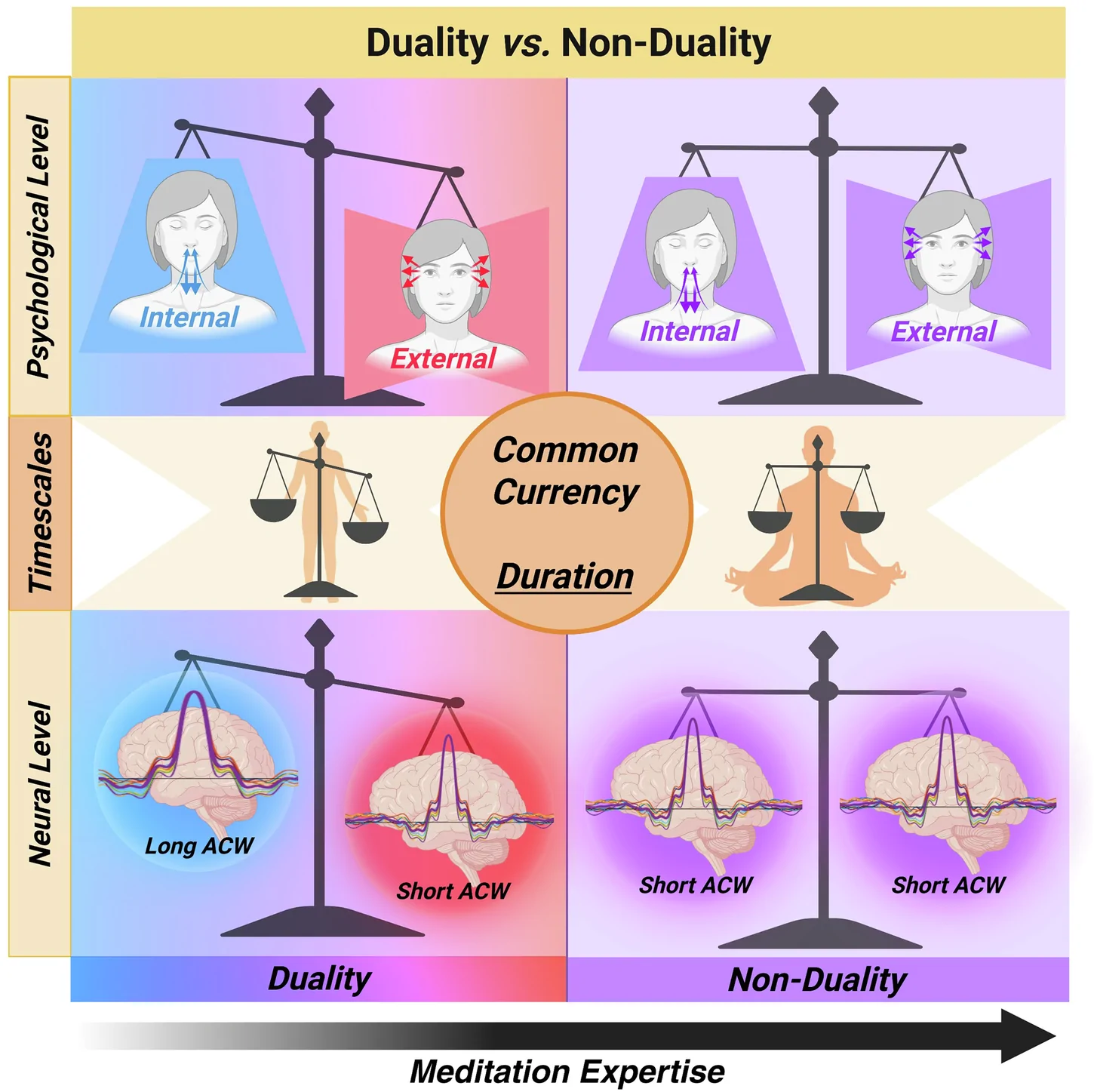
Graphical summary of the findings. Our study links the psychological to neural indices of duality and non-duality in study participants. We conceptualize duality in terms of internal and external processing, which we operationalized through distinct, i.e., internal and external attention tasks: breath-watching meditation (internal attention) and a visual oddball cognitive task (external attention). Consequently, we operationalize non-duality as a reduction (or even resolution) in the distinction between internal and external attention on both psychological (experience of non-duality) and neural (lack of difference in ACW between internal and external attention) levels. On the neural level, we found internal-external differentiation, or different durations of the intrinsic neural timescales, in novice meditators and controls, whereas we did not find such internal-external ACW difference (i.e., similar duration of timescales) in advanced meditators. The comprehensive approach we adopted allowed us to connect psychological assessments with neural indicators, demonstrating how non-duality manifests in advanced meditators. These results suggest that the lack of internal-external duration may be shared by both neural and mental features during non-dual states. Duration is measured at the neural level during internal and external attention: if both show no difference in their neural duration (ACW), they indicate a non-dual state at the psychological level. Though not measured explicitly here at the psychological level, this suggests that duration may also figure prominently in the experience of non-duality; this is, for instance, supported by subjective reports of shorter durations and timespans in meditative states^24,121,122^. Though tentative, this suggests that duration may be key in connecting neural and mental states, serving as their “common currency”^1,54,58^. Created in BioRender. Saketh, M. (2026) https://BioRender.com/jy4b7rm.

In our main sample, we observed the following findings: a) advanced meditators reported a greater sense of non-duality at the psychological level (as distinguished from various other possible cognitive factors, i.e., the other clusters of the MEDEQ) during breath-watching meditation compared to other groups; b) the duality between internal and external attention is also evident at the neural level: longer intrinsic neural timescales, measured by the ACW, were observed during breath-watching meditation (i.e., internal attention) compared to visual oddball task (i.e., external attention) in all subjects, indicating ‘neuro-temporal duality’; c) advanced meditators did not show this neuro-temporal duality, as they exhibited no significant difference in the duration of their timescales (ACW) between internal (breath-watching) and external (visual oddball) tasks. In other words, advanced meditators exhibited what we tentatively refer to as ‘neuro-temporal non-duality’ (see below for details), whereas novice meditators and controls did show a significant difference in ACW between these tasks. Lastly, d) non-duality at the psychological level correlated with the difference in ACW between internal and external tasks: a smaller difference in ACW between these tasks was associated with a greater sense of non-duality at the psychological level (while controlling for various cognitive factors). The main results were also further confirmed in a replication sample (see supplement). Together, our findings highlight the crucial role of the brain’s intrinsic neural timescales during internal-external processing in mediating the experience of non-duality in advanced meditators (Fig. 7).

### Non-duality and its psychological features

Our initial research aim was to determine if there were any psychological differences in non-duality among the three groups as measured during breath-watching meditation. We found greater experience of non-duality during breath-watching in advanced meditators compared to other groups. Phenomenology may shed a more detailed light on this finding. In contrast to mindfulness-based traditions that emphasize decentering, defusion, and meta-awareness^33,86–89^, the Isha Yoga tradition emphasizes the experience of non-dual awareness and provides methods to do so^39,42,44–46,90–92^. For example, one such method is breath-watching practice^39,93^, which is taught at both beginner^90,94,95^ and advanced levels^46,47^. At a beginner level, it is part of the Shambhavi Mahamudra Kriya^94–96^, a 21-minute practice that includes pranayama, chanting, and breath-watching, among others. At an advanced level, it is taught as part of Samyama, an 8-day advanced meditation retreat^39,46,47,97,98^, which requires learning Shambhavi Mahamudra and Shoonya (a non-dual meditation), among others. The word Samyama means the simultaneous practice of dharana (one-pointed focus), dhyana (meditative absorption), and samadhi (complete dissolution or meditative stillness), which are the last three limbs of the Ashtanga Yoga as described in the *Patanjali Yoga sutras*^99,100^. What is unique in this tradition is that Shoonya forms the foundation for Samyama. In other words, one is trained to experience absorption and dissolution states with awareness^30^, described in the scientific literature as singular non-dual awareness states (MPE + non-dual awareness)^8,30^.

Unlike the conventional understanding of breath-watching as a purely focused-attention meditation^33,87,88^, in this tradition, it is not an act of sustained focus but happens on the foundation of awareness. In mainstream models of meditation, practitioners are said to progress from focused attention to open monitoring to non-dual awareness^33,87,101^. However, in this tradition, the progression is reversed: non-dual awareness forms the foundation from the very beginning, even for breath-watching^39,45^. Meditation is not approached as something to be performed through deliberate effort, but as something that naturally unfolds through awareness. In this tradition, breath-watching is practised at the end of all other practices; for example, as the last 7 minutes of the Shambhavi Mahamudra practice^94,95^. Given this structure and phenomenological grounding, it is plausible that even novice meditators perform breath-watching from a background of awareness rather than in a foreground cognitive way through effortful attentional control. However, while novice meditators may experience non-dual awareness accompanied by maximal phenomenal content, advanced meditators are likely to experience non-dual awareness with substantially reduced or ideally without any phenomenal content.

Given this context, breath-watching involves maintaining internal attention on the breath (with a background of awareness) while ignoring distractions, such as mind-wandering or sensory stimuli^45,87,94,102,103^. In the early stages of this practice, such as in controls, the mind frequently wanders from the breath, requiring significant effort and awareness to refocus. However, this tendency to mind-wander diminishes with increased practice, and attention becomes more focused on the breath^88,89,101,104,105^. Reduced mind wandering and increased attention focus on the breath have been consistently reported in advanced meditators from different traditions^44,102,104–113^. Increased duration of attention on the breath and decreased duration of attention on internal mental contents could thus promote narrower temporal windows of attention in advanced meditators (i.e., shorter ACW during breath-watching meditation). With sustained practice, breath-watching likely fosters greater attentional segregation on the breath, allowing it to be perceived more distinctly from concurrent sensory or cognitive contents. Phenomenologically, this manifests as a steady and detailed awareness of the breath in which other thoughts or sensations lose salience. Given that shortening of the ACW reflects greater neural segregation, this mechanism may represent a common currency linking the phenomenological (attentional segregation of the breath from concurring stimuli) and neural (shortening of intrinsic timescales during breath-watching meditation, i.e., neural segregation) levels. With increasing expertise in breath-watching, as seen in our advanced meditators, there is a concurrent deidentification from internal mental contents, including one’s thoughts, with a reduction in mind wandering; this is supported by the psychological results in the MEDEQ. This process fosters a greater experience of non-dual awareness. Further supporting this, deidentification from one’s thoughts and contents has been shown to be the basis and thus enabling or prerequisite factor for the experience of non-duality^6,24,45^.

Remarkably, a growing body of literature shows psychological and neural changes that occur with increasing expertise in meditation^109,110,114–116^. In particular, it has been reported that advanced practitioners can easily focus on their breath without significant effort or conscious control. For instance, a functional magnetic resonance imaging (fMRI) study investigating the neural correlates of focused attention meditation in expert Tibetan Buddhist and age-matched novice meditators revealed an interesting pattern where expert meditators engaged lesser attentional networks than novices^117^. This suggests that as expertise increases, the need for conscious control diminishes, supporting the notion that extensive attentional training leads to a state where attention flows effortlessly toward the object of focus regardless of its internal or external source, enabling a non-dual state of awareness. This experience of the state of non-duality during breath-watching can be facilitated by the advanced meditator’s increased tendency to detach themselves and their mind from any specific contents, that is, increased non-attachment. This is exactly what we observed in our advanced mediators: higher degrees of non-duality, equanimity and non-attachment (questionnaires). It is important to mention that non-duality and equanimity are closely related to each other in the Indian spiritual traditions^31,99^. That further supports the primarily non-cognitive nature of non-dual experience: rather than recruiting various cognitive functions, non-duality seems to require the subjects’ deidentification from their respective contents to achieve a non-dual state. This is supported by our data showing the difference in non-dual experience compared to cognitive features like defusion, decentering, etc.

### Non-duality and its neural features

After identifying a greater experience of non-duality at the psychological level in advanced meditators, our next question was whether this would also be reflected at the neural level. First, we demonstrated that the psychological duality between internal (breath-watching) and external attention (a cognitive task) also manifests at the neural level. Specifically, the internal attention breath-watching task exhibited longer timescale durations (ACW) than the external attention cognitive task across all subjects.

Intrinsic neural timescales have been shown to provide temporal receptive windows that segregate (temporal segregation) and integrate (temporal integration) inputs of varying durations^1,57,66–73^. Paying attention to an external stimulus—such as during the cognitive task—is relatively easier and limited to the duration of the external stimulus, which leads to efficient temporal segregation, resulting in a narrower ACW^55,56,58,79^. In contrast, it is much harder to pay attention internally as the duration of our inner contents (e.g., thoughts, breathing) is longer^8,75,78,80^. Hence, while focusing on the breath, attention often shifts toward other mental contents, leading to lower temporal segregation and greater temporal integration of the breath with the other mental contents; this leads to a widening of the ACW, as we observed in our data. Similarly, self-related thoughts tend to exhibit longer duration (than external contents) and are thus associated with wider ACW than non-self-related thoughts^75^.

This neuro-temporal duality of the ACW between internal and external tasks was only observed in novice meditators and controls. In contrast, advanced meditators did not exhibit such an internal-external difference in their ACW, indicating neuro-temporal non-duality. Unlike in the internal attention task (breath-watching), no differences between the groups were observed during the external attention task. This indicates that all participants from all groups had similar timescales for external processing, as they were all exposed to the same stimuli with the same timing. Accordingly, the absence of an ACW difference between internal and external tasks in advanced meditators stems from a reduction in ACW during the internal breath-watching task. This suggests that advanced meditators use shorter neural timescales when practicing breath-watching—this highlights a key role for temporal segregation over temporal integration in focused-attention meditation, reflecting a key function of timescales^55,56,78^ in focused-attention meditation. However, as we discuss below, this reduction of ACW in the internal task by itself is not related to non-duality, but the difference between the internal and external attention matters.

Neural timescales shorten and lengthen with the temporal segregation and integration of stimuli, respectively^55–57,59^. Focused breath-watching requires the temporal segregation of the breath from distracting stimuli, such as thoughts. This process entails reduced internal attention to mental contents/thoughts and, subsequently, deidentification. In turn, deidentification facilitates the experience of no distinction between internal and external, resulting in non-duality in the advanced meditators^8,30^. The absence of significant ACW differences between the resting state and breath-watching, as well as between the resting state and the external task in advanced meditators, further supports their experience of a non-dual state. Our results suggest that non-duality may already be present during the resting state itself: these subjects no longer make a difference between rest, internal and external states in their ACW. This could possibly reflect their deep non-dual internal-external processing on the neural level, i.e., neuro-temporal non-duality, a neural trait feature indicating profound temporal organization of their brain’s neural dynamics (see also Cooper et al.^11^ for such reorganization model of meditation). Psychologically, this may be manifest in enhanced openness to experience, diminished ego-centered traits, and a trait-like meditative composure even during resting state in expert meditators. This is supported by higher scores in the non-attachment scale in this group.

### From internal-external ACW similarity to non-duality—neuropsychological relationships

The final line of evidence supporting the key role of neural duration for non-duality comes from the significant relationships we observed between the psychological and neural measures of non-duality. Greater experience of non-duality (as measured by the non-duality cluster of the MEDEQ) was associated with smaller differences in ACW between the internal attention breath-watching meditation and the external attention cognitive task. Importantly, other factors related to meditation depth (i.e., the other clusters of the MEDEQ) were not associated with it. Overall, our findings imply that individuals, through meditative training, can experience various events from a non-dual state of awareness rather than habitually categorizing everything into dualities such as self vs. other, good vs. bad, and internal vs. external. This finding aligns with other studies and reports, indicating a reduction in dualistic distinctions on psychological and neural levels in advanced meditators^5,6,9–11,18–21,24,25,28,118,119^. From the perspective of predictive processing, our findings may reflect a reduction in the hierarchical separation between predictive models governing internal and external streams of processing. Duality, in this view, may arise from distinct precision-weighting across internal and external hierarchies, which maintains separate predictions for internal and external sources of information. With meditative training, and particularly in advanced practitioners, this separation may diminish as the difference in the precision assigned to internally and externally related priors decreases^33,120^.

Our results suggest that the reduced difference between internal and external duration may be shared by both neural and mental levels during non-dual states. Specifically, duration is measured at the neural level during internal and external attention. If the neural durations for internal and external attention do not differ (that is, their ACW values converge), this may indicate a non-dual state at the psychological level. Though not measured explicitly here at the psychological level, this suggests that duration may also figure prominently in the experience of non-duality; this is, for instance, supported by subjective reports of shorter durations and timespans in meditative states^24,121,122^. Although tentative, this suggests that the duration of internal and external processing may be key in connecting neural and mental states, serving as their “common currency”^1,54,58^.

### Non-duality and its implications

Non-duality, observed cross-culturally, is considered the highest state of being and is part of the phenomenal repertoire of humanity^12,13,45,99,123–126^. Non-dual awareness has also been referred to as consciousness-as-such, pure consciousness, pure awareness, open awareness, choice-less awareness, turiya, satchitananada, buddha nature, etc^6,9,12^. Eastern traditions ascribe qualities such as bliss, timelessness, expansiveness, clarity, etc., with experience of non-dual awareness^9,24,45,99,123^. The experience of non-duality could have significant implications for individual happiness and well-being^12^. In this context, our findings indicate that non-duality is also part of the neural repertoire of humanity, with meditation serving as a valuable tool for cultivating this state. Our study underscores the value of integrating meditative practices into mental health and well-being programs by demonstrating that advanced meditators show non-duality at both psychological and neural levels. Meditation, a trainable skill, helps to cultivate resilience against everyday stressors and maintain a non-reactive, balanced state of mind^24,44,87,101,102,127–132^. As the prevalence of mental health issues increases globally^133–137^, qualities such as non-duality offer a pathway to achieving a deeper sense of peace and stability.

## Limitations

While ACW values suggest a non-dual state in advanced meditators during external tasks, the absence of a psychological measure of duration itself, such as duration of perception, in this state, is a limitation of the current study. Further, future research should incorporate paradigms that elicit stress and emotions to determine whether the non-dual state in advanced meditators is affected by such events. Finally, non-duality is a complex state with several features^5–8^ which, in addition to the lack of internal-external distinction as investigated here, may warrant future studies. Additionally, one may want to investigate the relationship of non-duality as studied here in relation to some of the traditional mindfulness techniques.

## Conclusion

Our study introduces a psychological and neural perspective on the experience of internal-external non-duality in advanced meditators. First, we show that advanced meditators exhibit greater experience of non-duality than control subjects during breath-watching meditation. Next, we demonstrate neural differences in ACW duration between internal attention (breath-watching meditation) and external attention (a cognitive task) in novice meditators and controls, indicating neuro-temporal duality. In contrast, advanced meditators no longer show these differences; instead, they exhibit greater similarity in ACW duration between internal and external tasks, suggesting neuro-temporal non-duality. Strongly supported by these and our various correlation and regression findings, we propose that the increased similarity in the brain’s timescales across internal and external tasks, i.e., their non-duality on the neural level, serves as a neural marker of non-duality on the psychological level in advanced meditators. Thus, the duration of the timescales may be shared by both neural and mental/experiential levels, e.g., brain and mind as their “ common currency.”

## Methods

### Participants

We re-analyzed already-published data^39^. 103 healthy adults were recruited and categorized into three groups: advanced meditators (ADV), novice meditators (NOV), and meditation-naive controls (CNT). Meditation-naive controls had no prior meditation experience and were recruited from the local student community. Meditators were recruited from the local Yoga Centres. Advanced meditators were those who had completed Samyama, an intensive meditation retreat conducted by the Isha Foundation^45,47,98^. Novice meditators were those who had completed Shambhavi Mahamudra^45,94^ but had not yet completed Samyama. Advanced meditators (*n* = 42, 18 females) had a mean (SD) age of 35.57 (6.81) years and a mean (SD) lifetime Isha Yoga practice duration of 5508 (2897) hours. Novice meditators (*n* = 33, 14 females) had a mean (SD) age of 31.66 (7.64) years with a mean (SD) lifetime practice duration of 1637 (1127) hours in Isha Yoga. Meditation-naive controls (*n* = 28, 16 females) had a mean (SD) age of 31.14 (6.38) years and no prior exposure to meditation or yoga schools; they were matched for age, education, and socioeconomic background. This study received approval from the NIMHANS Human Research Ethics Committee (NIMH/DO/ETHICS SUB-COMMITTEE MEETING/2018), and participants provided written informed consent before participation. No monetary compensation was provided. All ethical regulations relevant to human research participants were followed.

### Procedure

EEG studies were carried out at NIMHANS, Bengaluru, India. The EEG procedure consisted of four distinct sessions: alternate nostril breathing pranayama (6 minutes), breath-watching (15 minutes), visual oddball cognitive task (15 minutes), and Shoonya meditation (15 minutes) (refer to ref. ^39^). Both meditators and meditation-naive controls underwent these four sessions. Each session included a 4-minute rest period at the beginning and end. Participants were instructed to sit still and relax during these periods. Meditation depth during breath-watching was assessed right after that session.

#### Internal attention (breath-watching meditation)

Breath-watching is an eyes-closed, focused-attention meditation performed with a background of awareness. Controls were given the following instructions: pay attention to the breath, notice it when the mind wanders, and bring it back to the breath. Meditators practiced it as per the Isha Yoga tradition (see supplementary material for more details).

#### External attention (visual oddball task)

We utilized a paradigm called ANGEL (Assessing Neurocognition via Gamified Experimental Logic) in our study^138^. The task is performed with eyes open. This paradigm is based on the visual oddball paradigm, but it includes various gaming elements to increase engagement and allow for the investigation of decision-making in various contexts. ANGEL involves the simultaneous presentation of multiple audio and visual stimuli, challenging participants to make decisions amidst various distractions. Participants received performance feedback at the end of every two blocks to keep them motivated, and instructions and practice sessions were provided prior to the main session.

### Subjective questionnaires

- Non-attachment Scale (NAS-7): A 7-item version of the original nonattachment scale, known as the NAS-7^139^, was utilized to assess nonattachment as a trait feature of the subjects. Participants rated their agreement with seven statements, such as “I can let go of regrets and feelings of dissatisfaction about the past,” using a 7-point Likert scale ranging from 1 (Strongly Disagree) to 7 (Strongly Agree). The internal consistency was good (McDonald’s *ω* = 0.87, Cronbach’s *α* = 0.77).
- Meditation Depth Questionnaire (MEDEQ): MEDEQ, a 30-item questionnaire, evaluates the depth of meditation practice^38^. Participants rate their level of agreement with meditation-related statements on a scale from “0 = not at all” to “4 = very much.” The total score ranges from 0 to 120, with higher scores indicating deeper meditation. The questionnaire comprises five clusters: hindrances, relaxation, concentration, essential qualities, and non-duality. MEDEQ was acquired in 70 participants (controls—23, novice meditators—22, advanced meditators—25). The internal consistency for MEDEQ was excellent (McDonald’s omega = 0.99, Cronbach’s *α* = 0.96).
- The non-duality cluster of MEDEQ: Items 6 (I felt myself at one with everything), 7 (There was no subject and no object anymore), 16 (Thoughts had come completely to rest), 23 (My mind/consciousness expanded to an infinite space), 25 (There was no differentiation, comparison or judgment anymore. Everything could be as it was) and 26 (My mind, the field of consciousness and awareness was empty from thoughts, emotions and sensations) are part of the non-duality cluster of the meditation depth questionnaire^38^. This is a standard instrument for assessing the experience of non-duality, and it has been applied in many studies^39–43,122,140–148^. The final score for non-duality is obtained by adding the scores for all these items. A higher score indicates greater non-duality. The internal consistency was good (McDonald’s *ω* = 0.82, Cronbach’s *α* = 0.87).

### EEG acquisition and processing

The data was collected from 128-channel Geodesic EEG System 300 (Philips Neuro, USA). EEG recordings took place in a dimly lit, sound-attenuated chamber with a controlled ambient temperature (25 °C) and humidity (40–60%). Electrode impedance was kept below 50 kohm as per vendor recommendations. Stimulus presentation was managed using E-prime 2.0 software (Psychology Software Tools, Inc., Sharpsburg, PA, USA). Participants were seated 90 cm from a 34 cm × 27 cm LCD monitor displaying instructions. Data were amplified with the NetAmps300 amplifier, digitized at 24-bit resolution, and sampled at 1 kHz (DC amplifier). Electrode positioning followed geodesic spacing guidelines, with Cz as the online reference electrode and AFz as the ground electrode.

The following steps were used in processing the EEG data:

1. Raw EEG data were imported into EEGLAB v2021.0^149^ using the Philips ‘mff’ import plugin and pre-processed with in-house MATLAB scripts.
2. The data was resampled to 250 Hz and re-referenced to the average reference.
3. High-pass filtering ( > 0.5 Hz) and low-pass filtering ( < 80 Hz) were applied, along with a notch filter at 50 Hz.
4. Bad channels and segments were automatically rejected using the artifact subspace reconstruction (ASR) plugin with a five-standard-deviation threshold. On average, 11.19% (min: 1.33%, max: 45.16%) of data were removed for advanced meditators, 12.61% (min: 1.3%, max: 66.7%) for novice meditators, and 11.21% (min: 0.38%, max: 57.17%) for controls.
5. Scalp muscle, ocular, and ECG artifacts were identified and removed using the ICALABEL plugin (90% threshold) after running independent component analysis (ICA) with the ‘infomax’ method. On average, 3.14 components (min: 0, max: 21) were removed for advanced meditators, 2.41 components (min: 0, max: 21) for novice meditators, and 3.99 components (min: 0, max: 25) for controls.
6. Channel interpolation was performed using neighboring electrodes with a spherical spline approach.

### Data cleaning

EEG and psychological data were collected from 103 participants overall (CNT = 28, NOV = 33, ADV = 42). Of these, 102 participants completed the breath-watching task (CNT = 28, NOV = 33, ADV = 41)^39^, and 78 participants completed the visual oddball cognitive task (CNT = 22, NOV = 24, ADV = 32). Not all participants performed the visual oddball task due to time constraints. Among these participants, we had clean ACW data for 97 participants (CNT = 26, NOV = 33, ADV = 38) during breath-watching meditation and for 75 participants (CNT = 21, NOV = 22, ADV = 32) during the visual oddball task. No participants were excluded; differences in sample size reflect task completion and data availability. The meditation depth questionnaire was introduced^38^ after data collection had commenced and was therefore administered to a subset of 70 participants (CNT = 23, NOV = 22, ADV = 25). Finally, 53 participants (CNT = 16, NOV = 18, ADV = 19) had complete and clean datasets comprising both breath-watching and visual oddball EEG recordings, along with responses to the meditation depth questionnaire. This subset was used for correlation analyses examining the relationship between EEG measures and the non-duality cluster of the meditation depth questionnaire.

### Autocorrelation window

An ACF was calculated in MATLAB (version 2023b) using a custom code^150^. This function was applied to 10-second EEG epochs at each electrode with a 1-second sliding across relevant session data. Within each 10-second epoch, ACF was computed on 1-second windows (with 50% overlap) and averaged to get a smooth estimate of ACF. On this, ACW-50 was computed as the first lag where the ACF decays to 50% of its maximal value^57^. Finally, the ACW-50 mean (from 20% trimmed mean) values were calculated from the 10-second epochs for each of the electrodes for each participant.

**Fig. 8:**
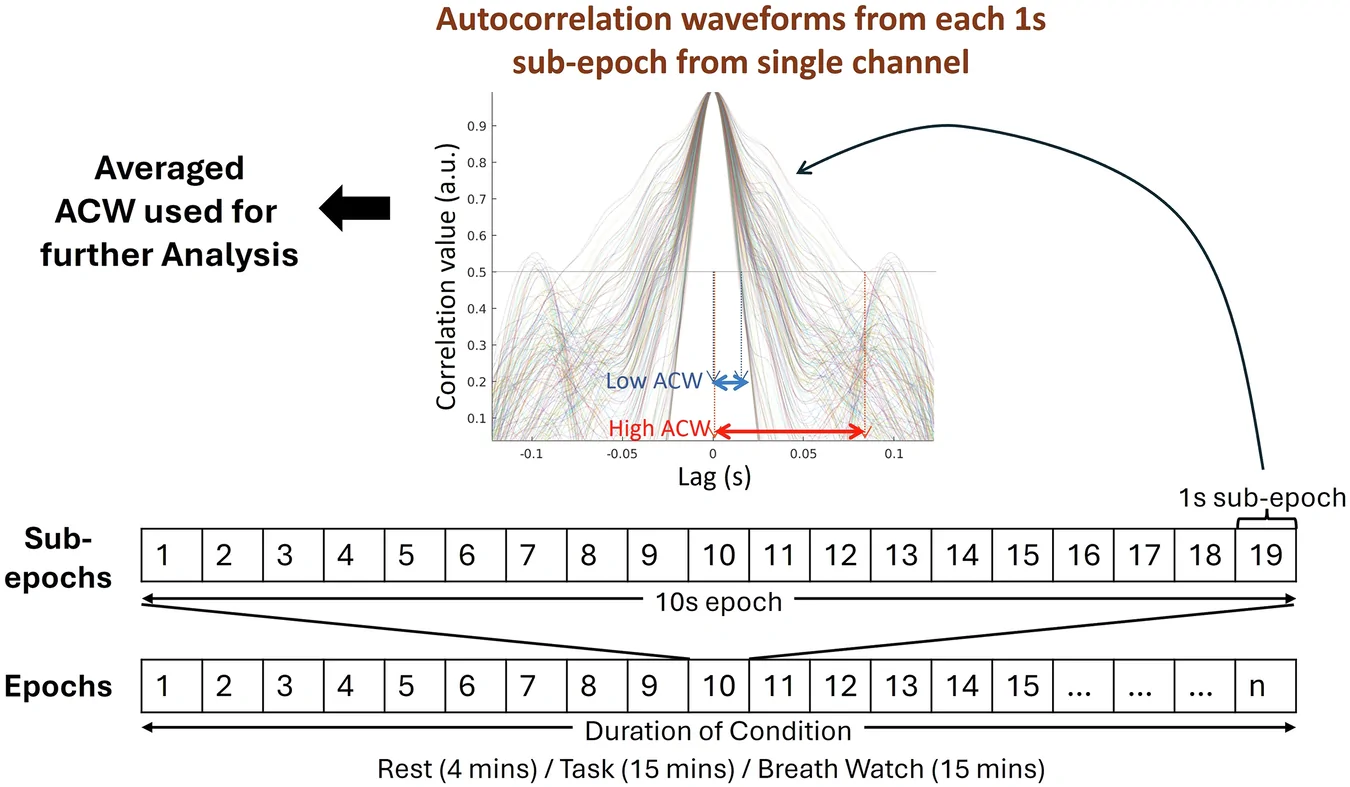
Computation of autocorrelation window (ACW). Schematic illustration of intrinsic neural timescale computation using autocorrelation window (ACW) analysis. Continuous EEG data from each condition are segmented into epochs and 1 s sub-epochs, from which autocorrelation waveforms are computed for individual channels. ACW values from all sub-epochs are then averaged and used for further analysis, with longer ACWs reflecting prolonged temporal integration.

We then conducted a whole-scalp topographic analysis of ACW differences between breath-watching and the cognitive task (Supplementary Fig. 12). This analysis revealed that condition-related effects were spatially localized to frontal regions, with no comparable effects observed in posterior regions. To systematically evaluate regional effects, we defined anatomically informed electrode clusters (frontal, temporal, parietal, and occipital). We then tested condition-related differences in ACW within each cluster. Consistent with the topographic maps, only the frontal cluster showed significant condition-related effects, while temporal, parietal, and occipital clusters did not. The ACW mean values from the frontal cluster (channels: ‘AF8’,‘AF4’,‘Fp2’,‘AFz’,‘Fp1’,‘AF3’,‘AF7’) were considered for the final analysis (Fig. 8).

### Statistics

The statistical analyses were conducted using RStudio Version 1.4.1106, employing several R packages, including ggplot2^151^, ggsci^152^, ggstatsplot^153^, dplyr^154^, effectsize^155^, performance^156^, and car^157^ for necessary analyses. Parametric tests (Student *t* test, Mixed ANOVA) or non-parametric tests (Kruskal–Wallis) were used per the normality assumptions. Effect sizes were reported with 95% confidence intervals, and significance was set at *p* < 0.05. Pearson’s *r* was used to estimate correlation when both the variables were continuous. Further, Spearman’s rho method was used to estimate the correlation when one of the variables was discrete. Visualization was done using the ggplot library, and each dot was colored as per group. A line of fit was drawn with a 95% CI shadow. We performed simple and hierarchical linear regression analyses in R using the lm() function from the stats package. Model comparison and additional diagnostics were performed using packages such as car^157^ and performance^156^. Additionally, we performed linear mixed effects analyses in R using lmerTest package^158^.

We conducted a mediation analysis using the lavaan package in R^159^ to test the hypothesis of how different variables (e.g., MEDEQ) mediate the relationship between the predictor (e.g., internal ACW) and the outcome (e.g., the non-duality cluster of MEDEQ). A structural equation model was specified to estimate the direct path (c’) and the indirect path (a*b). To determine the significance of the indirect effect, we employed bootstrapping with 5000 resamples and generated 95% percentile confidence intervals. Partial mediation was inferred if both the direct (c’) and indirect (ab) effects were significant, whereas full mediation was inferred if the indirect effect was significant, but the direct effect was not.

### Replication dataset

We analyzed data from 43 expert meditators from our previous study on Vipassana meditation^160^. The data were acquired with the same EGI 128-channel EEG system employed in the current study. The dataset included senior Vipassana practitioners (*n* = 22; 11 females; mean age = 54.2 ± 12.6 years; meditation experience = 13.0 ± 4.4 years; cumulative meditation hours = 10,364 ± 5,229) and teachers (*n* = 21; 10 females; mean age = 51.8 ± 12.2 years; meditation experience = 16.3 ± 5.3 years; cumulative meditation hours = 15,349 ± 9,307). No meditation-naive control group was included. As part of the experimental protocol, participants performed Anapana (breath-watching meditation) and completed a visual oddball task (ANGEL^138^), among others. Non-duality was not studied. ACW analysis was conducted using the exact same procedure as in the current study.

### Ethics and inclusion statement

Data collection and analysis were conducted at the Center for Consciousness Studies, Department of Neurophysiology, NIMHANS, Bengaluru, India, and involved local researchers. Roles and responsibilities were defined among collaborators prior to the commencement of the study. All researchers involved in data collection and analysis received appropriate training in EEG acquisition, data handling, and participant safety. The study was approved by the Institutional Ethics Committee of NIMHANS (NIMH/DO/ETHICS SUB-COMMITTEE MEETING/2018). All participants provided written informed consent prior to participation, in accordance with institutional guidelines and the Declaration of Helsinki. Participation was voluntary, and participants were free to withdraw at any time. The study procedures did not involve significant risk to participants. All data were anonymized prior to analysis to ensure participant confidentiality. EEG recordings were conducted following standard safety protocols, and all equipment was used in accordance with established laboratory guidelines.

### Reporting summary

Further information on research design is available in the Nature Portfolio Reporting Summary linked to this article.

## Supplementary information

Transparent Peer Review file

Supplementary Information

Description of Additional Supplementary Files

Supplementary Data 1

Reporting Summary

## Data Availability

The human EEG data are not publicly available due to ethical requirements and privacy concerns. The numerical data that can be used to generate figures are within the Supplementary Information (Supplementary Data). Any other data is available from the corresponding author upon reasonable request.

## Code availability

An ACF was calculated in MATLAB (version 2023b) using an open-source code^150^.
