# Supplementary material for: Non-duality in brain and experience of advanced meditators—key role for intrinsic neural timescales
Source: Commun Biol. 2026 Jun 12;9:1261. doi: 10.1038/s42003-026-10473-9 (PMC13616908; doi:10.1038/s42003-026-10473-9)
Supplement: Supplementary file 1 [file 42003_2026_10473_MOESM1_ESM.pdf]

nature portfolio

Peer Review File

Non-duality in brain and experience of advanced meditators – Key role for Intrinsic Neural Timescales

Corresponding Author: Dr Malipeddi Saketh

This file contains all reviewer reports in order by version, followed by all author rebuttals in order by version.

Version 0:

Reviewer comments:

Reviewer #1

(Remarks to the Author)
This manuscript investigates differences in autocorrelation window (ACW) of the EEG between experienced meditators, novice meditators and controls during breath-focused meditation and a visual oddball task. The authors report that while all groups show similar ACW values during the oddball task, novices and controls show increased ACW during the meditation task whereas experienced meditators maintain the same ACW across both conditions. The authors interpret this interaction pattern as evidence for non-duality in advanced practitioners. While the research question is interesting and the topic timely, I have substantial methodological concerns that prevent me from recommending publication in its current form. The study suffers from inadequate methodological documentation, unjustified analytical choices, and failure to control for or rule out alternative explanations that are more parsimonious than the proposed non-duality interpretation.

Major Concerns:

1. Missing Critical Methodological Information: Eyes Open vs. Closed
The manuscript does not specify whether participants had their eyes open or closed during the breath-focused meditation condition. This is a critical omission that fundamentally affects interpretation of the results.
The visual oddball task necessarily requires eyes open while breath-focused meditation in the Isha Yoga tradition is typically practiced with eyes closed. Eyes-open vs. eyes-closed conditions produce dramatically different EEG spectral profiles, particularly in the Alpha band. Due to their rhythmic and sustained nature, Alpha oscillations create strong autocorrelation and would substantially lengthen ACW measurements. If eyes were closed during meditation and open during the oddball task, the beginners' pattern (ACW decrease from meditation to task) could be entirely explained by alpha suppression when opening eyes, which is a completely normal physiological response unrelated to the task. The finding that experienced meditators maintain constant ACW across conditions - could then reflect one of two well-documented phenomena:1) Experienced meditators are known to maintain alpha rhythms even with eyes open (documented in multiple studies). 2) Some meditation reduce alpha even during eyes-closed practice given that alpha is inversely related to attention. Neither interpretation requires invoking non-duality. The interaction could simply reflect how meditation training modulates the basic physiological response to eyes opening/closing.
The authors have to explicitly state the eyes condition for both tasks. If the conditions differed, an analysis controlling for this confound has to be provided. At minimum, alpha power analysis for all conditions and groups have to be provided to demonstrate that the ACW pattern is not simply an eyes-state effect. If eyes state did differ between conditions, the entire interpretation requires fundamental revision.

2. Unjustified and Problematic Electrode Selection
The use of a few frontal electrodes for ACW calculation is not justified and no a priori hypothesis or citation is provided for why frontal regions specifically should reflect non-duality or the construct being measured. As it stands, this appears to be an arbitrary post-hoc choice. The interaction pattern reported (beginners showing task-related ACW changes, experts not) could be entirely dependent on which electrodes were analyzed. Without full topographic analysis, selective reporting cannot be ruled out. Were other regions analyzed but only frontal sites reported because they showed the desired pattern? It is entirely possible that posterior regions show a different pattern, which would fundamentally change the interpretation.

3. Failure to Address the Relationship Between ACW and Frequency Spectrum
The ACW measure is fundamentally related to the frequency composition of the signal, yet the manuscript does not address

this relationship or demonstrate that ACW captures information beyond what standard spectral analysis would reveal. It is well known that ACW is heavily influenced by spectral composition - signals with more low-frequency content show longer autocorrelation. The oddball task typically increases beta/gamma activity (attention, discrimination, response preparation) and suppresses alpha Therefore, the beginners' ACW decrease during the task could simply reflect normal task-induced high-frequency activity that shortens ACW. The experts' maintained ACW could reflect either: (a) maintained alpha/theta despite task demands, or (b) failure to show normal task-related spectral changes. Power spectral density analysis for both groups in both conditions has to be provided and a demonstration that the interaction pattern remains after controlling for spectral composition.

Reviewer #2

(Remarks to the Author)
Comments to the Authors

In this manuscript, the authors have proposed developing a neuro-temporal marker of non-duality linked to meditation experiences.

The topic of the manuscript is highly relevant to the existing meditation literature, as several previous studies have consistently recommended exploring more objective markers linked to the underlying mechanisms of meditative practices. Non-duality is indeed a critical component of such practices.

General Comments:
For the most part, the manuscript is very well written. The authors have provided very clear and descriptive definitions of non-duality and all the assessment measures. The rationale for the hypotheses and the logical flow are explained quite rigorously.

However, I have some specific concerns and suggestions below. If addressed, I would be glad to recommend this manuscript for acceptance.

Specific Concerns:

Introduction:
In the statement, “An emerging body of literature shows that advanced meditators report experiencing such non-dual awareness. What are the neural correlates of such non-dual awareness?” the transition between the two statements is abrupt. Please provide examples and explanations of a few non-dual experiences from a couple of the studies that the authors have cited here. Then justify why the authors want to investigate the neural correlates of “such non-dual awareness.”

Methods & Results:

My main concern is related to the participant numbers allocated in the Materials & Methods section, specifically for the Advanced group (n = 42). However, the actual number of participants analyzed for the EEG results is not clear from their statement in the Data Cleaning section: “EEG data were collected from 103 participants. The meditation depth questionnaire (see below) was published after data collection had begun and was therefore administered to 70 participants.”

Please provide an explanation for dropping participants rather than directing the reader to see below.

Please report n for each group in the Results section to better understand the power and effect sizes. If participants were dropped or excluded, please explain the loss of participants during the study in the Methods section.

It seems the authors successfully demonstrated their hypothesis of non-duality in advanced meditators, indexed by a lack of difference in ACW between breath-watching and the oddball task. However, a major concern here is that the number of participants in the Advanced group was 42, overall 103, from whom EEG was recorded; however, in Figure 2, results are reported from fewer than actual participants recruited. On what basis were they excluded or dropped? I would not be surprised if losing 4–5 participants from a group could change the directionality of the results in a consequential way.

Version 1:

Reviewer comments:

Reviewer #1

(Remarks to the Author)
I thank the authors for this important additional analysis of the influence of spectral changes on the group differences of ACW, confirming that spectral differences were at least partly influencing the observed effects. Also the topographic analysis gives valuable additional information. Given these information the paper can now be accepted

Reviewer #2

(Remarks to the Author)
The authors have addressed all the concerns I had. I am glad to endorse this manuscript in its current form.

Open Access This Peer Review File is licensed under a Creative Commons Attribution 4.0 International License, which permits use, sharing, adaptation, distribution and reproduction in any medium or format, as long as you give appropriate credit to the original author(s) and the source, provide a link to the Creative Commons license, and indicate if changes were made.
In cases where reviewers are anonymous, credit should be given to 'Anonymous Referee' and the source.
The images or other third party material in this Peer Review File are included in the article's Creative Commons license, unless indicated otherwise in a credit line to the material. If material is not included in the article's Creative Commons license and your intended use is not permitted by statutory regulation or exceeds the permitted use, you will need to obtain permission directly from the copyright holder.
To view a copy of this license, visit https://creativecommons.org/licenses/by/4.0/

Response to Reviewers

Manuscript Title:

Non-duality in brain and experience of advanced meditators – Key role for Intrinsic Neural Timescales

Reviewer #1 (Remarks to the Author):

This manuscript investigates differences in autocorrelation window (ACW) of the EEG between experienced meditators, novice meditators and controls during breath-focused meditation and a visual oddball task. The authors report that while all groups show similar ACW values during the oddball task, novices and controls show increased ACW during the meditation task whereas experienced meditators maintain the same ACW across both conditions. The authors interpret this interaction pattern as evidence for non-duality in advanced practitioners. While the research question is interesting and the topic timely, I have substantial methodological concerns that prevent me from recommending publication in its current form. The study suffers from inadequate methodological documentation, unjustified analytical choices, and failure to control for or rule out alternative explanations that are more parsimonious than the proposed non-duality interpretation.

Major Concerns:

Reviewer's Comment 1: Missing Critical Methodological Information: Eyes Open vs. Closed

The manuscript does not specify whether participants had their eyes open or closed during the breath-focused meditation condition. This is a critical omission that fundamentally affects interpretation of the results.

The visual oddball task necessarily requires eyes open while breath-focused meditation in the Isha Yoga tradition is typically practiced with eyes closed. Eyes-open vs. eyes-closed conditions produce dramatically different EEG spectral profiles, particularly in the Alpha band. Due to their rhythmic and sustained nature, Alpha oscillations create strong autocorrelation and would substantially lengthen ACW measurements. If eyes were closed during meditation and open during the oddball task, the beginners' pattern (ACW decrease from meditation to task) could be entirely explained by alpha suppression when opening eyes, which is a completely normal physiological response unrelated to the task. The finding that experienced meditators maintain constant ACW across conditions - could then reflect one of two well-documented

phenomena:1) Experienced meditators are known to maintain alpha rhythms even with eyes open (documented in multiple studies). 2) Some meditation reduce alpha even during eyes-closed practice given that alpha is inversely related to attention. Neither interpretation requires invoking non-duality. The interaction could simply reflect how meditation training modulates the basic physiological response to eyes opening/closing.

The authors have to explicitly state the eyes condition for both tasks. If the conditions differed, an analysis controlling for this confound has to be provided. At minimum, alpha power analysis for all conditions and groups have to be provided to demonstrate that the ACW pattern is not simply an eyes-state effect. If eyes state did differ between conditions, the entire interpretation requires fundamental revision.

Author's Response: We thank the reviewer for highlighting the important issue of eyes-open versus eyes-closed states and their potential influence on alpha activity and ACW. We now explicitly clarify that the breath-watching condition was performed with eyes closed, whereas the cognitive (oddball) task was performed with eyes open.

We incorporated this in the revised manuscript (Page 35, Line 892): "*Breath-watching is an eyes-closed focused-attention meditation performed with a background of awareness*" and **(Page 35, Line 898):** "*The task is performed with eyes open*."

To address the concern that differences in ACW may be driven by eye-state-related changes in alpha power, we conducted analysis of alpha power across groups and conditions (Figure 1 below, Supplementary Figure 5). A mixed-design ANOVA (Table 1 below, Supplement Table 6) revealed no significant Group × Condition interaction in alpha power ($F(2,48) = 0.24$, $p = 0.788$), indicating that task-related modulation of alpha activity did not differ across groups. If the observed ACW effects were primarily driven by eye-state-related alpha changes, one would expect corresponding group differences in alpha modulation. However, such differences were not observed. In contrast, we observed condition-related changes in ACW that differed across groups, with reductions from breath-watching to task in controls and novices, but relatively stable values in the advanced group.

To further test whether these effects were attributable to alpha power, we conducted a linear mixed-effects model analysis (Table 2 below, Supplement Table 7) including alpha power as a covariate:

$$ACW \sim Group \times Condition + Alpha + (1 \mid Subject)$$

The Group × Condition interaction remained significant after controlling for alpha power ($F(2, 87.68) = 3.21$, $p = 0.045$), indicating that the observed ACW effects are not fully explained by alpha-band activity. Taken together, these findings suggest that while alpha power is influenced by eye state, the group differences in ACW cannot be reduced to eye-state-related alpha modulation alone and likely reflect additional aspects of neural temporal dynamics.

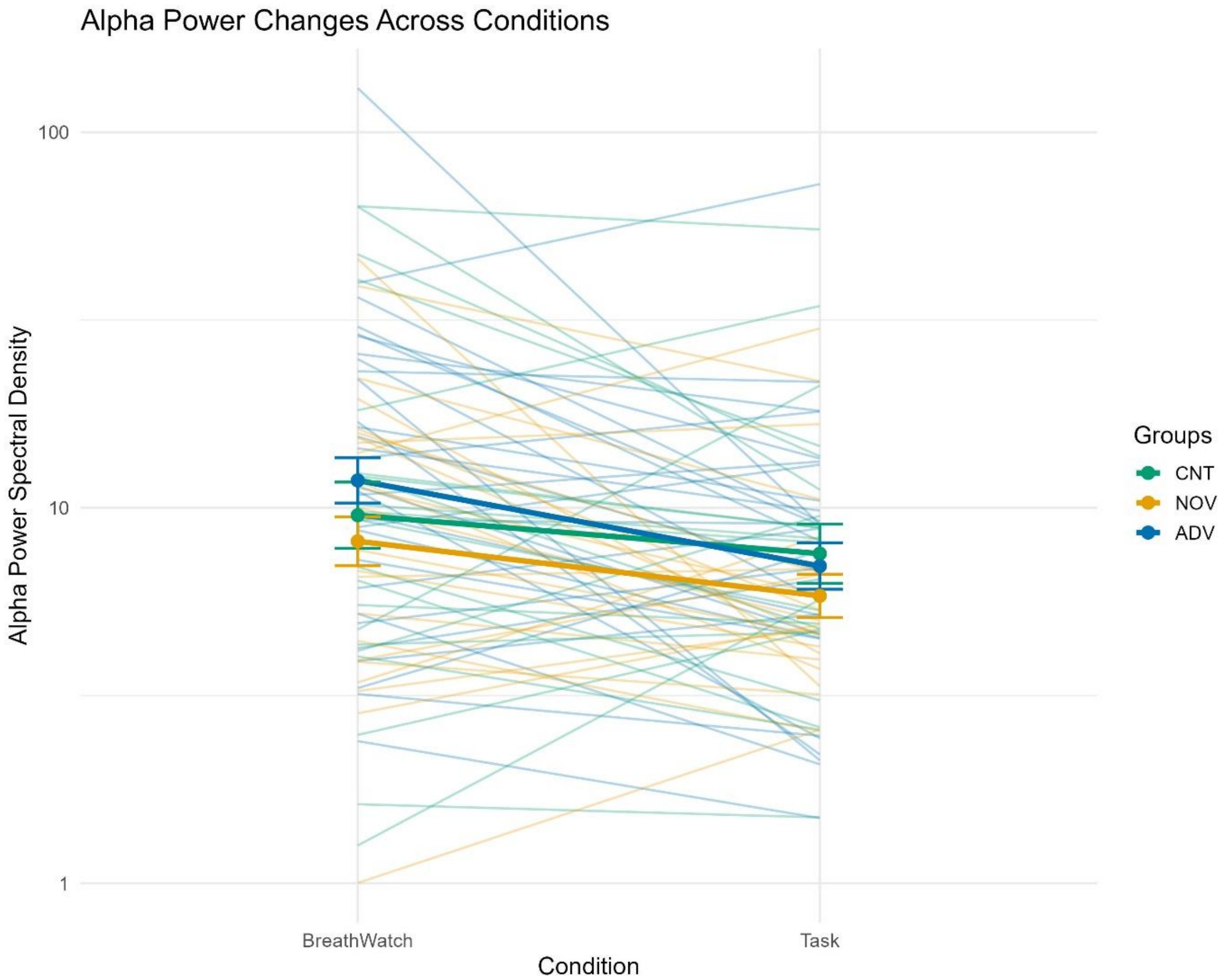

Figure 1: *Alpha power across breath-watching (eyes closed) and cognitive task (eyes open) conditions in advanced meditators (ADV), controls (CNT), and novices (NOV). Thin lines represent individual participants, and thicker lines with points indicate group means. Error bars denote standard error of the mean. Alpha power is shown on a logarithmic scale.*

Table 1: Mixed ANOVA of alpha power across group and condition

	**Effect**	**df (num, den)**	**MSE**	**F**	**Effect size (ges)**	**p value**
1	Group	2,72	325.37	0.88	0.016	0.418
2	Condition	1,72	169.96	8.22	0.038	0.005**

3	**Group × Condition**	2,72	169.96	0.32	0.003	**0.725**

Results of a mixed-design ANOVA testing the effects of Group, Condition, and their interaction on the dependent variable. Effect sizes are reported as generalized eta squared (ges). Significance levels: **p < .01. df: degrees of freedom; MSE: mean squared error.

Table 2: Linear mixed-effects model analysis of ACW with group, condition, and alpha power as a covariate

**Effect**	**df (num, den)**	**F**	**p-value**
Group	2, 94.79	0.70	0.497
Condition	1, 92.25	47.09	< 0.001***
Alpha (covariate)	1, 163.78	2.05	0.154
**Group × Condition**	2, 87.68	3.21	**0.045***

Type III sums of squares were used. Degrees of freedom were estimated using Satterthwaite's method. Significance codes: *** p < .001, * p < .05; non-significant effects are not marked.

We incorporated this information in the revised manuscript (Page 18-20, Lines 445 – 461):

"*Relationship between ACW and frequency spectrum*

We have so far shown that advanced meditators exhibit significantly less or lower ACW difference between eyes-closed internal (breath-watching) and eyes-open external (oddball) conditions compared to both controls and novices. Are these changes related to differential eyes-state alpha effects between the groups?

To address this, we conducted analysis of alpha power across groups and conditions (Supplementary Figure 5). A mixed-design ANOVA (Supplement Table 6) revealed no significant Group × Condition interaction in alpha power ($F(2,48) = 0.24$, $p = 0.788$), indicating that task-related modulation of alpha activity did not differ across groups. If the observed ACW effects were primarily driven by eye-state-related alpha changes, one would expect corresponding group differences in alpha modulation. To further test whether these effects were attributable to alpha power, we conducted a linear mixed-effects model analysis

(Supplement Table 7) including alpha power as a covariate: ACW ~ Group × Condition + Alpha + (1 | Subject). The Group × Condition interaction remained significant after controlling for alpha power (F(2, 87.68) = 3.21, p = 0.045), indicating that the observed ACW effects are not fully explained by alpha-band activity. Taken together, these findings suggest that while alpha power is influenced by eye state, the group differences in ACW cannot be reduced to eye-state-related alpha modulation alone and likely reflect additional aspects of neural temporal dynamics."

Reviewer's Comment 2: Unjustified and Problematic Electrode Selection

The use of a few frontal electrodes for ACW calculation is not justified and no a priori hypothesis or citation is provided for why frontal regions specifically should reflect non-duality or the construct being measured. As it stands, this appears to be an arbitrary post-hoc choice. The interaction pattern reported (beginners showing task-related ACW changes, experts not) could be entirely dependent on which electrodes were analyzed. Without full topographic analysis, selective reporting cannot be ruled out. Were other regions analyzed but only frontal sites reported because they showed the desired pattern? It is entirely possible that posterior regions show a different pattern, which would fundamentally change the interpretation.

Author's Response: We thank the reviewer for raising this important concern regarding electrode selection. We agree that restricting analysis to a subset of electrodes without adequate justification could introduce bias. To address this, we conducted a whole-scalp topographic analysis of ACW differences between breath-watching and the cognitive task (Figure 2 below, Supplementary Figure 12). This analysis revealed that condition-related effects were spatially localized to frontal regions, with no comparable effects observed in posterior regions. To systematically evaluate regional effects, we defined anatomically informed electrode clusters (frontal, temporal, parietal, and occipital). We then tested condition-related differences in ACW within each cluster. Consistent with the topographic maps, only the frontal cluster (AF8, AF4, Fp2, AFz, Fp1, AF3, AF7) showed significant condition-related effects, while temporal, parietal, and occipital clusters did not. Importantly, this analysis indicates that the reported effects are not dependent on arbitrary electrode selection, but instead reflect a spatially specific pattern evident at the whole-scalp level. We have now clarified this procedure and included the topographic analysis in the revised manuscript to ensure transparency and to rule out the possibility of selective reporting.

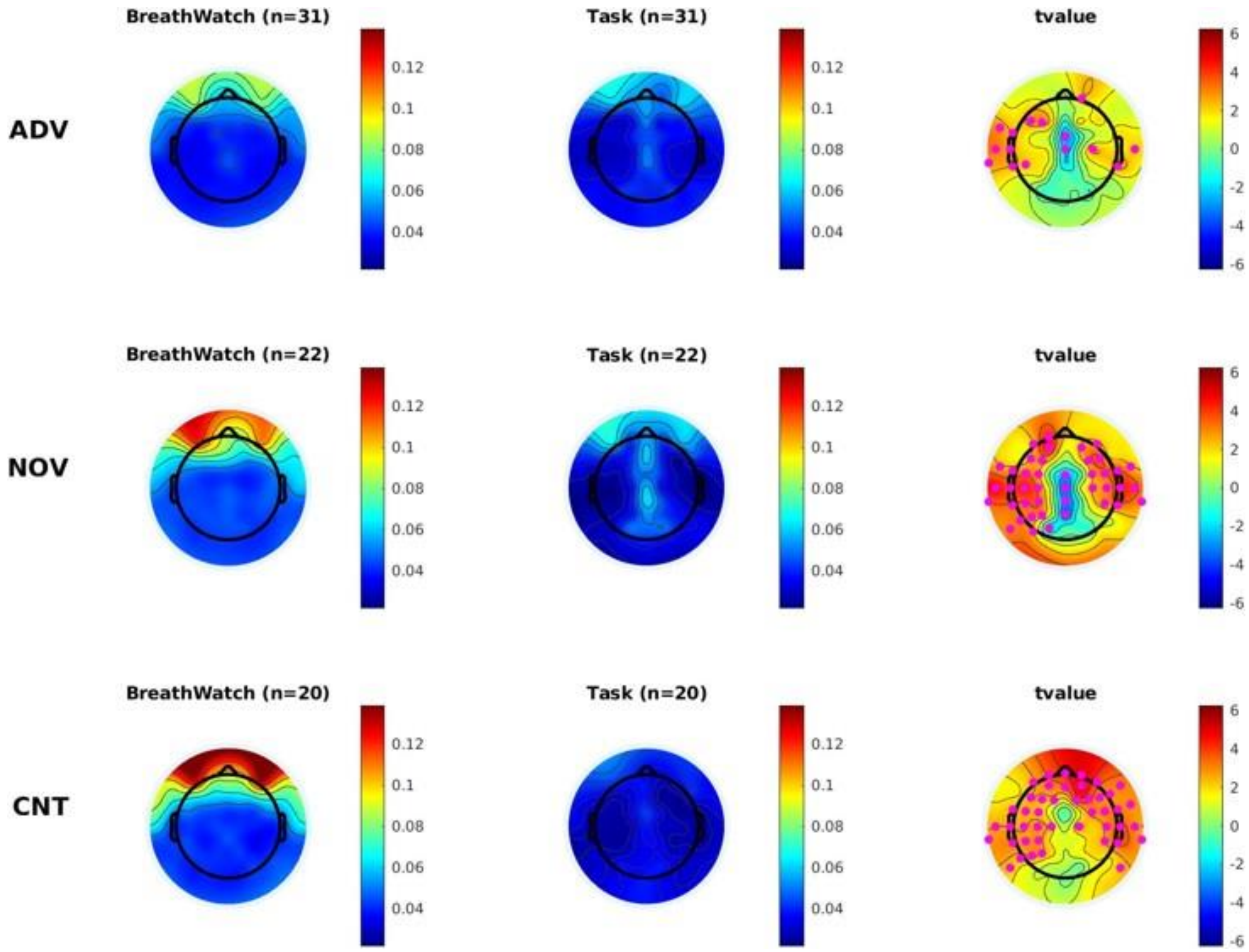

Figure 2: Topographic maps of ACW during breath-watching and cognitive task conditions in advanced meditators (ADV), novices (NOV), and controls (CNT). ACW was computed for 10s epochs with 1s window and 50% overlap. The first and second columns show condition-specific maps, and the third column shows t-values for the contrast (breath-watching − task). Robust paired t-tests with threshold-free cluster enhancement (TFCE) were used. Statistically significant electrodes are indicated in pink.

We have incorporated this information in the revised manuscript (Page 38, Lines 985-992:

"*We then conducted a whole-scalp topographic analysis of ACW differences between breath-watching and the cognitive task (Supplementary Figure 12). This analysis revealed that condition-related effects were spatially localized to frontal regions, with no comparable effects observed in posterior regions. To systematically evaluate regional effects, we defined anatomically informed electrode clusters (frontal, temporal, parietal, and occipital). We then tested condition-related differences in ACW within each cluster. Consistent with the topographic maps, only the frontal cluster showed significant condition-related effects, while temporal, parietal, and occipital clusters did not.*"

Reviewer's Comment 3: Failure to Address the Relationship Between ACW and Frequency Spectrum

The ACW measure is fundamentally related to the frequency composition of the signal, yet the manuscript does not address this relationship or demonstrate that ACW captures information beyond what standard spectral analysis would reveal. It is well known that ACW is heavily influenced by spectral composition - signals with more low-frequency content show longer autocorrelation. The oddball task typically increases beta/gamma activity (attention, discrimination, response preparation) and suppresses alpha. Therefore, the beginners' ACW decrease during the task could simply reflect normal task-induced high-frequency activity that shortens ACW. The experts' maintained ACW could reflect either: (a) maintained alpha/theta despite task demands, or (b) failure to show normal task-related spectral changes. Power spectral density analysis for both groups in both conditions has to be provided and a demonstration that the interaction pattern remains after controlling for spectral composition.

Author's Response: We thank the reviewer for highlighting the important relationship between ACW and the frequency spectrum. We agree that ACW is influenced by the spectral composition of neural signals, particularly low-frequency power.

To address this, we conducted power spectral analysis across delta, theta, alpha, beta1, beta2, and gamma bands for all groups (advanced, novice, and controls) and conditions (breath-watching and cognitive task) (Figure 3 below, Supplementary Figure 6). A mixed-design ANOVA (Table 3 below, Supplement Table 8) with Group (Advanced meditators, Novice meditators, Controls) as a between-subject factor and Condition (Breath-watching, Cognitive Task) and Frequency Band (Delta, Theta, Alpha, Beta1, Beta2, and Gamma) as within-subject factors revealed a trend-level Condition × Band interaction, $F(1.37, 98.73) = 3.08$, $p = .069$, suggesting modest task-related spectral shifts across frequency bands. Importantly, the Group × Condition × Band interaction was not significant, $F(2.74, 98.73) = 0.60$, $p = .602$, indicating no evidence for differential spectral modulation between groups. If the observed ACW differences were primarily driven by spectral composition, one would expect corresponding group differences in task-related spectral modulation. However, such differences were not observed.

To further test whether ACW effects were reducible to spectral composition, we conducted a linear mixed-effects model analysis (Table 4 below, Supplement Table 9) including band-limited power (delta, theta, alpha, beta1, beta2, gamma) as covariates:

$$ACW \sim Group \times Condition + Delta + Theta + Alpha + Beta1 + Beta2 + Gamma + (1 \mid Subject)$$

The Group × Condition interaction in ACW remained significant after controlling for spectral power, $F(2, 92.16) = 3.11$, $p = .049$, indicating that the observed ACW differences are not fully explained by band-limited spectral power alone. Taken together, these findings suggest that while ACW is related to spectral composition, it captures aspects of neural temporal dynamics that are not fully reflected in conventional spectral measures. Importantly, the interaction pattern remains after controlling for spectral composition.

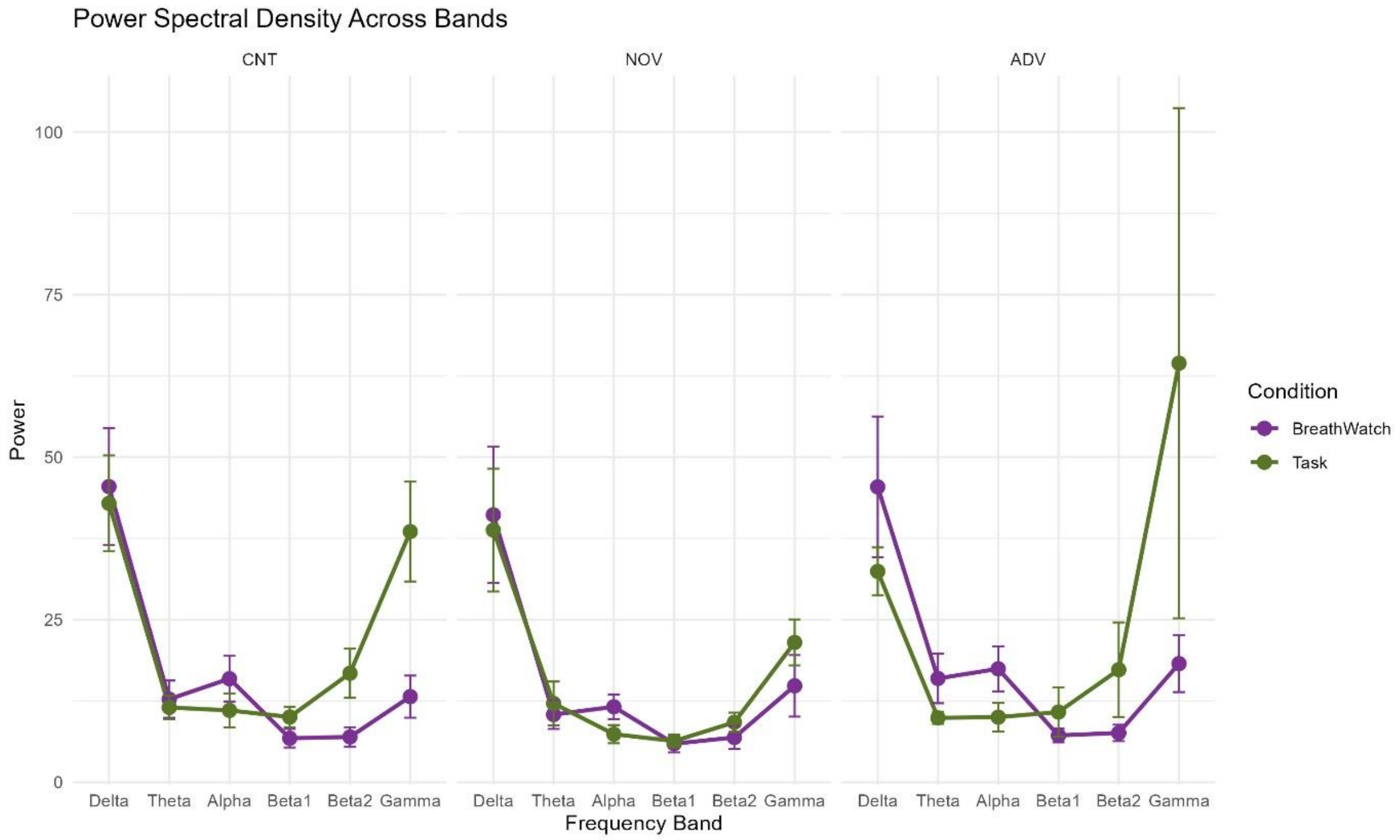

Figure 3: Power spectral density across frequency bands for breath-watching and task conditions in controls (CNT), novices (NOV), and advanced meditators (ADV). Points represent group means, and error bars denote standard error of the mean. Colours indicate condition (breath-watching and task).

Table 3: Mixed ANOVA of power spectral density across group, condition, and frequency band

	**Effect**	**df (num, den)**	**MSE**	**F**	**Effect size (ges)**	**p-value**
1	Group	2, 72	5445.15	0.70	0.004	0.499
2	Condition	1, 72	4445.31	0.64	0.001	0.428
3	Group × Condition	2, 72	4445.31	0.09	< 0.001	0.915
4	Frequency Band	1.38, 99.38	8158.62	11.40	0.057	< 0.001***

5	Group × Band	2.76, 99.38	8158.62	0.52	0.005	0.657
6	Condition × Band	1.37, 98.73	6243.47	3.08	0.012	0.069^{+}
7	**Group × Condition × Band**	2.74, 98.73	6243.47	0.60	0.005	**0.602**

Effect sizes are reported as generalized eta squared (ges). Degrees of freedom were corrected using the Greenhouse–Geisser method where appropriate. Significance levels: *** p < .001, + p < .10. MSE: mean squared error.

Table 4: Linear mixed-effects model analysis of ACW across group and condition with spectral power covariates

**Effect**	**df (num, den)**	**F**	**p-value**
Group	2, 95.08	1.41	0.250
Condition	1, 106.66	44.19	< 0.001***
Delta	1, 157.18	50.94	< 0.001***
Theta	1, 154.49	2.16	0.144
Alpha	1, 159.06	8.41	0.004**
Beta1	1, 139.05	6.30	0.013*
Beta2	1, 160.00	7.03	0.009**
Gamma	1, 154.46	24.38	< 0.001***
**Group × Condition**	2, 92.16	3.11	**0.049***

Type III sums of squares were used, and degrees of freedom were estimated using Satterthwaite's method. Significance levels: *** p < .001, ** p < .01, * p < .05; non-significant effects are not marked.

We have incorporated this information in the revised manuscript (Page 19-20, Lines 462 – 487):

"Additionally, to address the relationship between ACW and the overall frequency spectrum, and to study if our ACW results hold true after controlling for spectral composition, we conducted power spectral analysis across delta, theta, alpha, beta1, beta2, and gamma bands

for all groups (advanced, novice, and controls) and conditions (breath-watching and cognitive task) (Supplementary Figure 6). A mixed-design ANOVA (Supplement Table 8) with Group (Advanced meditators, Novice meditators, Controls) as a between-subject factor and Condition (Breath-watching, Cognitive Task) and Frequency Band (Delta, Theta, Alpha, Beta1, Beta2, and Gamma) as within-subject factors revealed a trend-level Condition × Band interaction, $F(1.37, 98.73) = 3.08$, $p = .069$, suggesting modest task-related spectral shifts across frequency bands. Importantly, the Group × Condition × Band interaction was not significant, $F(2.74, 98.73) = 0.60$, $p = .602$, indicating no evidence for differential spectral modulation between groups. If the observed ACW differences between our three groups were primarily driven by spectral composition, one would expect corresponding group differences in task-related spectral modulation. However, such differences were not observed.

To further test whether ACW effects were reducible to spectral composition, we conducted a linear mixed-effects model analysis (Supplement Table 9) including band-limited power (delta, theta, alpha, beta1, beta2, gamma) as covariates:

$$ACW \sim Group \times Condition + Delta + Theta + Alpha + Beta1 + Beta2 + Gamma + (1 \mid Subject)$$

The Group × Condition interaction in ACW remained significant after controlling for spectral power, $F(2, 92.16) = 3.11$, $p = .049$, indicating that the observed ACW differences are not fully explained by band-limited spectral power alone. Taken together, these findings suggest that while ACW is related to spectral composition, it captures aspects of neural temporal dynamics that are not fully reflected in conventional spectral measures. This is in line with other studies showing similar findings [78,84]. Importantly, this means that the group differences in ACW between advanced meditators and controls cannot be accounted for their spectral composition as this showed no corresponding group differences. "

Reviewer #2 (Remarks to the Author):

Comments to the Authors

In this manuscript, the authors have proposed developing a neuro-temporal marker of non-duality linked to meditation experiences.

The topic of the manuscript is highly relevant to the existing meditation literature, as several previous studies have consistently recommended exploring more objective markers linked to the underlying mechanisms of meditative practices. Non-duality is indeed a critical component of such practices.

Reviewer's General Comments:

For the most part, the manuscript is very well written. The authors have provided very clear and descriptive definitions of non-duality and all the assessment measures. The rationale for the hypotheses and the logical flow are explained quite rigorously.

However, I have some specific concerns and suggestions below. If addressed, I would be glad to recommend this manuscript for acceptance.

Authors response: We thank the reviewer for the good feedback provided.

Reviewer's Specific Concerns:

Introduction:

In the statement, "An emerging body of literature shows that advanced meditators report experiencing such non-dual awareness. What are the neural correlates of such non-dual awareness?" the transition between the two statements is abrupt. Please provide examples and explanations of a few non-dual experiences from a couple of the studies that the authors have cited here. Then justify why the authors want to investigate the neural correlates of "such non-dual awareness."

Author's response: We thank the reviewer for this helpful suggestion. We agree that the transition between these statements was abrupt and that additional clarification of the phenomenology of non-dual awareness would strengthen the motivation for our study. In the revised manuscript, we now include brief descriptions of non-dual experiences reported in prior studies.

We incorporated this information in the revised manuscript (Page 2, Lines 54-63): "*An emerging body of literature shows that advanced meditators report experiencing non-dual awareness* [5,6,10,11,18,20,21,24–30]*, often accompanied by experiential qualities such as a sense of presence, clarity, emptiness, bliss, and a timeless feeling, among others*[30]*. For example, one participant in a study on mindfulness-induced selflessness described the experience as follows: "It was emptiness, as if the self fell out of the picture. There was an experience but it had no address, it was not attached to a center or subject ..."*[18]*. These phenomenological reports suggest that non-dual awareness involves a fundamental alteration in the structure of experience, particularly in terms of the dissolution of the usual dual self–world boundaries. However, the neural mechanisms underlying such non-dual states remain poorly understood.*"

Reviewer's comments - Methods & Results:

My main concern is related to the participant numbers allocated in the Materials & Methods section, specifically for the Advanced group (n = 42). However, the actual number of participants analyzed for the EEG results is not clear from their statement in the Data Cleaning section: "EEG data were collected from 103 participants. The meditation depth questionnaire (see below) was published after data collection had begun and was therefore administered to 70 participants."

Please provide an explanation for dropping participants rather than directing the reader to see below.

Author's response: We thank the reviewer for highlighting this important point. We agree that the description of participant numbers across analyses was not sufficiently clear in the original manuscript.

We would like to clarify that no participants were excluded arbitrarily from the study. The variation in sample sizes reflects differences in task completion and data availability across measures. Specifically, not all participants completed both the breath-watching and visual oddball tasks due to time constraints, and the meditation depth questionnaire was introduced after data collection had commenced, resulting in its administration to a subset of participants. Additionally, correlation analyses were conducted on participants for whom complete datasets (EEG data from both tasks and questionnaire responses) were available.

To address this concern, we have revised the Methods section to explicitly report participant numbers for each task and analysis, including group-wise breakdowns, to improve transparency and clarity.

We incorporated this information in the revised manuscript (Page 37, Lines 959 - 974): "*EEG and psychological data were collected from 103 participants overall (CNT = 28, NOV = 33, ADV = 42). Of these, 102 participants completed the breath-watching task (CNT = 28, NOV = 33, ADV = 41)* [38]*, and 78 participants completed the visual oddball cognitive task (CNT = 22, NOV = 24, ADV = 32). Not all participants performed the visual oddball task due to time constraints. Among these participants, we had clean auto-correlation window (ACW) data for 97 participants (CNT = 26, NOV = 33, ADV = 38) during the breath-watching meditation and for 75 participants (CNT = 21, NOV = 22, ADV = 32) during the visual oddball task. No participants were excluded; differences in sample size reflect task completion and data availability. The meditation depth questionnaire was introduced* [37] *after data collection had commenced and was therefore administered to a subset of 70 participants (CNT = 23, NOV = 22, ADV = 25). Finally, 53 participants (CNT = 16, NOV = 18, ADV = 19) had complete and clean datasets comprising both breath-watching and visual oddball EEG recordings, along with responses to the meditation depth questionnaire. This subset was used for correlation analyses examining the relationship between EEG measures and the non-duality cluster of the meditation depth questionnaire.*"

Please report n for each group in the Results section to better understand the power and effect sizes. If participants were dropped or excluded, please explain the loss of participants during the study in the Methods section.

Author's response: We thank the reviewer for this important point. We reported n for each group in the figure captions in the results section for transparency (Figure 2: Page 10, Lines 216-17, 219, and 221; Figure 3: Page 15, Lines 366, 368, 370, 372, and 374; Figure 4: Page 18, Lines 433-37; Figure 5: Page 21, Line 523-24). No participants were excluded; differences in sample size reflect task completion and data availability. We updated the methods section to include these details (Page 37, Lines 959 – 974).

It seems the authors successfully demonstrated their hypothesis of non-duality in advanced meditators, indexed by a lack of difference in ACW between breath-watching and the oddball task. However, a major concern here is that the number of participants in the Advanced group was 42, overall 103, from whom EEG was recorded; however, in Figure 2, results are reported

from fewer than actual participants recruited. On what basis were they excluded or dropped? I would not be surprised if losing 4–5 participants from a group could change the directionality of the results in a consequential way.

Author's response: We thank the reviewer for pointing this out. We would like to clarify that no participants were excluded or dropped from the study. Differences in sample size reflect task completion and data availability. We updated the methods section to include these details (Page 37, Lines 959 – 974).
