# Supplementary material for: Non-duality in brain and experience of advanced meditators—key role for intrinsic neural timescales
Source: Commun Biol. 2026 Jun 12;9:1261. doi: 10.1038/s42003-026-10473-9 (PMC13616908; doi:10.1038/s42003-026-10473-9)
Supplement: Supplementary file 2 [file 42003_2026_10473_MOESM2_ESM.pdf]

Non-duality in brain and experience of advanced meditators – Key role for Intrinsic Neural Timescales

Malipeddi Saketh[1]*, Arun Sasidharan[1], Bianca Ventura[2,3], Rahul Venugopal[1], Clemens Christian Bauer[4], Prejaas K.B. Tewarie[5,6], Ravindra P. Nagendra[1], Seema Mehrotra[7], John P John[8], Balachundhar Subramaniam[9], Steven Laureys[5,10], Bindu M Kutty[1#], Georg Northoff[2,11#]*

[1]Centre for Consciousness Studies, Department of Neurophysiology, Old Administrative Block, National Institute of Mental Health and Neuro Sciences (NIMHANS), Bengaluru, 560029, Karnataka, India

[2]The Royal's Institute of Mental Health Research & University of Ottawa, Brain and Mind Research Institute, Centre for Neural Dynamics, Faculty of Medicine, University of Ottawa, 145 Carling Avenue, Rm. 6435, Ottawa, ON, K1Z 7K4, Canada

[3]School of Psychology, University of Ottawa, 136 Jean-Jacques Lussier, Ottawa, ON K1N 6N5, Canada

[4]McGovern Institute for Brain Research, Massachusetts Institute of Technology, Cambridge, MA, 02139, USA

[5]Joint International Research Unit on Consciousness, CERVO Brain Research Centre, University of Laval, Québec City, G1J 2G3, Canada

[6]Sir Peter Mansfield Imaging Centre, School of Physics, University of Nottingham, Nottingham, NG7 2RD, United Kingdom

[7]Department of Clinical Psychology, National Institute of Mental Health and Neuro Sciences (NIMHANS), Bengaluru, 560029, Karnataka, India

[8]Multi-modal Brain Image Analysis Laboratory, Department of Psychiatry, National Institute of Mental Health and Neuro Sciences (NIMHANS), Bengaluru, 560029, Karnataka, India

[9]Sadhguru Center for a Conscious Planet, Department of Anesthesiology, Critical Care and Pain Medicine, Beth Israel Deaconess Medical Center, Suite 621, One Brookline Place, Brookline, 02445, USA

[10]Joint International Research Unit on Neuroplasticity, University of Liège, Liège, 4000, Belgium

[11]Centre for Cognition and Brain Disorders, Hangzhou Normal University, Tianmu Road 305, Hangzhou, Zhejiang Province, 310013, China

*Corresponding authors

[#] These authors jointly supervised this work

Corresponding authors:

Malipeddi Saketh, Email address: sakethreddy21@gmail.com

Georg Northoff, Email address: gnorthoff@theroyal.ca

Supplementary Note

Isha Yoga tradition

Isha Yoga is a mind-body practice tradition originating from India [1–5]. It provides methods to experience deeper states of consciousness and overcome human suffering. In contrast to mindfulness-based traditions that emphasize decentering, defusion, and meta-awareness [6–10], the Isha Yoga tradition emphasizes the experience of non-dual awareness and provides methods to do so [1–4,11–14]. For example, one such method is breath-watching practice [2,15], which is taught at both beginner [12,16,17] and advanced levels [4,18]. At a beginner level, it is part of the Shambhavi Mahamudra Kriya (offered as Inner Engineering) [16,17,19], a 21-minute practice that includes pranayama, chanting, and breath-watching, among others. One becomes eligible for Shoonya after learning Shambhavi. Shoonya is practice of "conscious non-doing" [2,11,20] that cultivates de-identification with body and mind, leading to non-dual states of awareness [2]. At an advanced level, breath-watching is taught as part of the Samyama meditation retreat [4,18,21,22], which requires learning Shambhavi, Shoonya meditation, and Hatha Yoga [23,24]. In this study, participants who had completed Samyama retreat were considered as advanced meditators (5508 mean hours), and those who completed either Shambhavi or Shoonya were considered novice meditators (1637 mean hours). Participants with no meditation history were considered as meditation-naïve controls.

Samyama is an 8-day advanced meditation retreat that includes initiation into breath-watching, among other practices [2,4,18,21,22,25]. The word Samyama means the simultaneous practice of dharana (one-pointed focus), dhyana (meditative absorption), and samadhi (complete dissolution or meditative stillness), which are the last three limbs of the Ashtanga Yoga as described in the *Patanjali Yoga sutras* [26,27]. What is unique in this tradition is that Shoonya forms the foundation for Samyama. In other words, one is trained to experience absorption and dissolution states with awareness [28], described in the scientific literature as singular non-dual awareness states (MPE + non-dual awareness) [28,29]. This is described in the tradition as " *a state where you become fully aware that you are not the body, you are not the mind, you are not the world around*." [22] Further, non-dual awareness is seen as the deepest core or the background layer of human consciousness, "*the source of creation.*" [19,30]

As mentioned earlier, breath-watching is taught at both the beginner (as part of Shambhavi Mahamudra) and the advanced levels (as part of Samyama). Unlike the conventional understanding of breath-watching as a focused-attention meditation [6,8,9], in this tradition it is

not an act of sustained focus but happens on the foundation of awareness. In mainstream models of meditation, practitioners are said to progress from focused attention to open monitoring to non-dual awareness [6,8,31]. However, in this tradition, the progression is reversed—non-dual awareness forms the foundation from the very beginning, even for breath-watching [2,3]. Meditation is not approached as something to be performed through deliberate effort, but as something that naturally unfolds through awareness. In this tradition, breath-watching is practiced at the end of all other practices; for example, as the last 7 minutes of the Shambhavi Mahamudra practice [16,17]. Given this structure and phenomenological grounding, it is plausible that even novice meditators perform breath-watching from a background of awareness rather than through effortful attentional control. However, while novice meditators may experience non-dual awareness accompanied by maximal phenomenal content, advanced meditators are likely to experience non-dual awareness with substantially reduced phenomenal content.

We used breath-watching in this study as the instructions are simple and understandable, even for meditation-naïve controls. In contrast, controls found it difficult to understand the instructions for Shoonya practice, with several reporting that they engaged in open monitoring instead, leading to a substantial variability in the practice of Shoonya in our dataset. Finally, using breath-watching as an internal attention condition allowed us to conceptualize duality as the difference in internal and external attention, and non-duality as the lack of differentiation between the two.

This perspective of non-duality as the background layer in the Isha Yoga tradition aligns with the implicit-explicit gradient [28,32] and the topographic re-organization model of meditation [33], where non-dual awareness, which is always present as a background layer in an implicit manner, becomes fully explicit in proficient meditation practitioners. Additionally, these perspectives are in line with the broader wisdom from the Indian Knowledge Systems. According to the Mandukya Upanishad [34], there are four states of consciousness: Jagrathi (or wakefulness), Swapna (or dream sleep), Sushupthi (or deep, dreamless sleep), and Turiya (non-dual awareness or pure consciousness) [27,34–36]. Sage Patanjali in his Yoga sutras describes the Turiya state as "*Tada Drashtuh Svarupe Avasthanam*" (Yoga Sutra 1.3) – (in the turiya state), Seer or Self is established in its original state – and "*Vritti sarupyam itaratra*" (Yoga Sutra 1.4) – in all other states, Seer or Self is identified with the mind [26]. In addition, this state is described to be a natural state within a human being, forming a substratum over which other states (waking, dreaming, sleep) happen [26,27,36], much like the implicit-explicit gradient of non-dual awareness that is orthogonal to global states and phenomenal content [28]. Yoga also says specific

mental patterns – called Kleshas or hindrances – obscure ones natural state of awareness [26] (implicit zone of the gradient). In this context, Yogic practices like breath-watching meditation are seen to be methods that reduce hindrances so that one returns to their original state of consciousness. Overall, the phenomenological grounding of the breath-watching practice suggests that participants experience non-dual awareness as an ever-present background state rather than as purely absorptive states devoid of awareness.

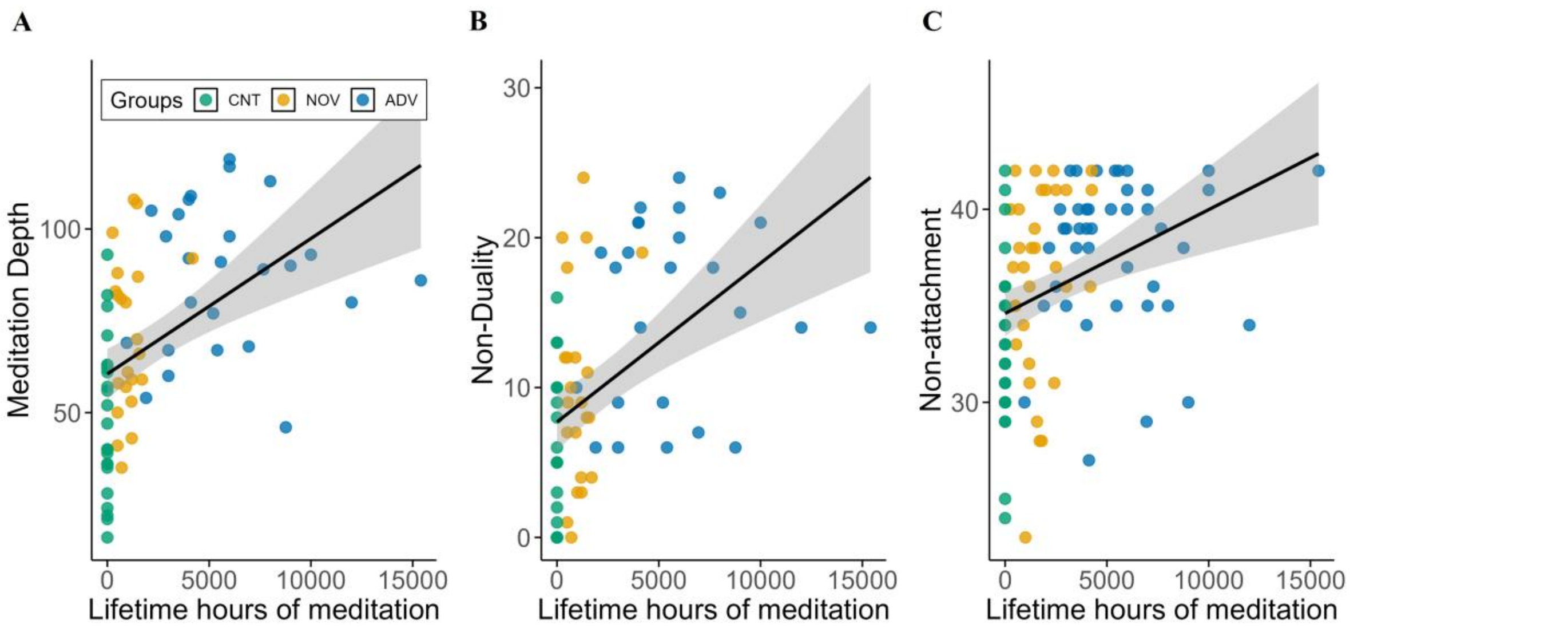

Supplementary Figure 1. Scatterplot illustrating the positive correlation between lifetime hours of meditation and **A.** meditation depth ($P_{Spearman} = 0.60$, $p < 0.0001$, $n = 70$), **B.** non-duality ($P_{Spearman} = 0.57$, $p < 0.0001$, $n = 70$), and **C.** non-attachment ($P_{Spearman} = 0.44$, $p < 0.0001$, $n = 103$). Shaded region represents the standard error of the mean.

Supplementary Table 1: Breath-watching meditation vs Cognitive Task - Results of the Mixed ANOVA Examining the Effects of Group and Condition on ACW Mean

**Effect**	**Sum_Sq**	**df_num**	**Error SS**	**df_den**	**F value**	**Effect size (η^2_p)**	**p-value**
Intercept	0.68911	1	0.18898	70	255.249		p < 2.2e-16 ****
Groups	0.004	2	0.18898	70	0.7411	0.021	p = 0.4803 ^{ns}
Condition	0.06274	1	0.11607	70	37.8391	0.351	p = 4.2e-08 ****
Interaction (Groups × Condition)	0.01247	2	0.11607	70	3.7607	0.097	p = 0.0281 *

This table presents the results of a mixed ANOVA assessing the main effects of Group (Controls, Novice Meditators, Advanced Meditators) and Condition (Breath-Watching Meditation, Cognitive Task) and their interaction on ACW mean. The table includes the sum of squares (Sum_Sq), numerator degrees of freedom (df_num), error sum of squares (Error_SS), denominator degrees of freedom (df_den), F-values (F_value), effect size (partial eta-squared), and corresponding p-values (p_value). Significance levels are denoted as: ns = Not significant ($p \geq 0.05$), * = $p < 0.05$, ** = $p < 0.01$, *** = $p < 0.001$, **** = $p < 0.0001$.

Supplementary Table 2: Post-hoc pair-wise comparisons for breath-watching meditation vs cognitive task Mixed ANOVA results

Contrast	Estimate	Std. Error	df	t-ratio	p-value
ADV BreathWatch - CNT BreathWatch	-0.03233	0.01620	70	-1.997	0.3545
ADV BreathWatch - NOV BreathWatch	-0.02470	0.01570	70	-1.569	0.6210
**ADV BreathWatch - ADV Task**	**0.01830**	**0.01030**	**70**	**1.770**	**0.4918**
ADV BreathWatch - CNT Task	0.02954	0.01270	70	2.325	0.1980
ADV BreathWatch - NOV Task	0.02173	0.01250	70	1.740	0.5108
CNT BreathWatch - NOV BreathWatch	0.00763	0.01740	70	0.438	0.9979
CNT BreathWatch - ADV Task	0.05063	0.01400	70	3.606	0.0073
**CNT BreathWatch - CNT Task**	**0.06186**	**0.01290**	**70**	**4.804**	**0.0001**
CNT BreathWatch - NOV Task	0.05406	0.01460	70	3.708	0.0053
NOV BreathWatch - ADV Task	0.04300	0.01350	70	3.182	0.0255
NOV BreathWatch - CNT Task	0.05423	0.01430	70	3.802	0.0040
**NOV BreathWatch - NOV Task**	**0.04643**	**0.01230**	**70**	**3.782**	**0.0042**
ADV Task - CNT Task	0.01123	0.00982	70	1.144	0.8611
ADV Task - NOV Task	0.00343	0.00954	70	0.359	0.9992
CNT Task - NOV Task	-0.00780	0.01060	70	-0.738	0.9765

Supplementary Table 3: Breath-watching meditation vs Resting state - Results of the Mixed ANOVA Examining the Effects of Group and Condition on ACW Mean

**Effect (Breath-watch vs Rest)**	**Sum_Sq**	**df_num**	**Error_SS**	**df_den**	**F_value**	**Effect size (η^2_p)**	**p_value**
Intercept	1.21358	1	0.274508	76	335.9909		< 2.2e-16 ****
Groups	0.00716	2	0.274508	76	0.9908	0.025	0.3760335 [ns]
Condition	0.01742	1	0.097757	76	13.5393	0.151	0.0004341 ***
Interaction (Groups × Condition)	0.00946	2	0.097757	76	3.6774	0.088	0.0298918 *

This table presents the results of a mixed ANOVA assessing the main effects of Group (Controls, Novice Meditators, Advanced Meditators) and Condition (Breath-Watching Meditation, Resting state) and their interaction on ACW mean. The table includes the sum of squares (Sum_Sq), numerator degrees of freedom (df_num), error sum of squares (Error_SS), denominator degrees of freedom (df_den), F-values (F_value), effect size (partial eta-squared), and corresponding p-values (p_value). Significance levels are denoted as: ns = Not significant ($p \geq 0.05$), * = $p < 0.05$, ** = $p < 0.01$, *** = $p < 0.001$, **** = $p < 0.0001$.

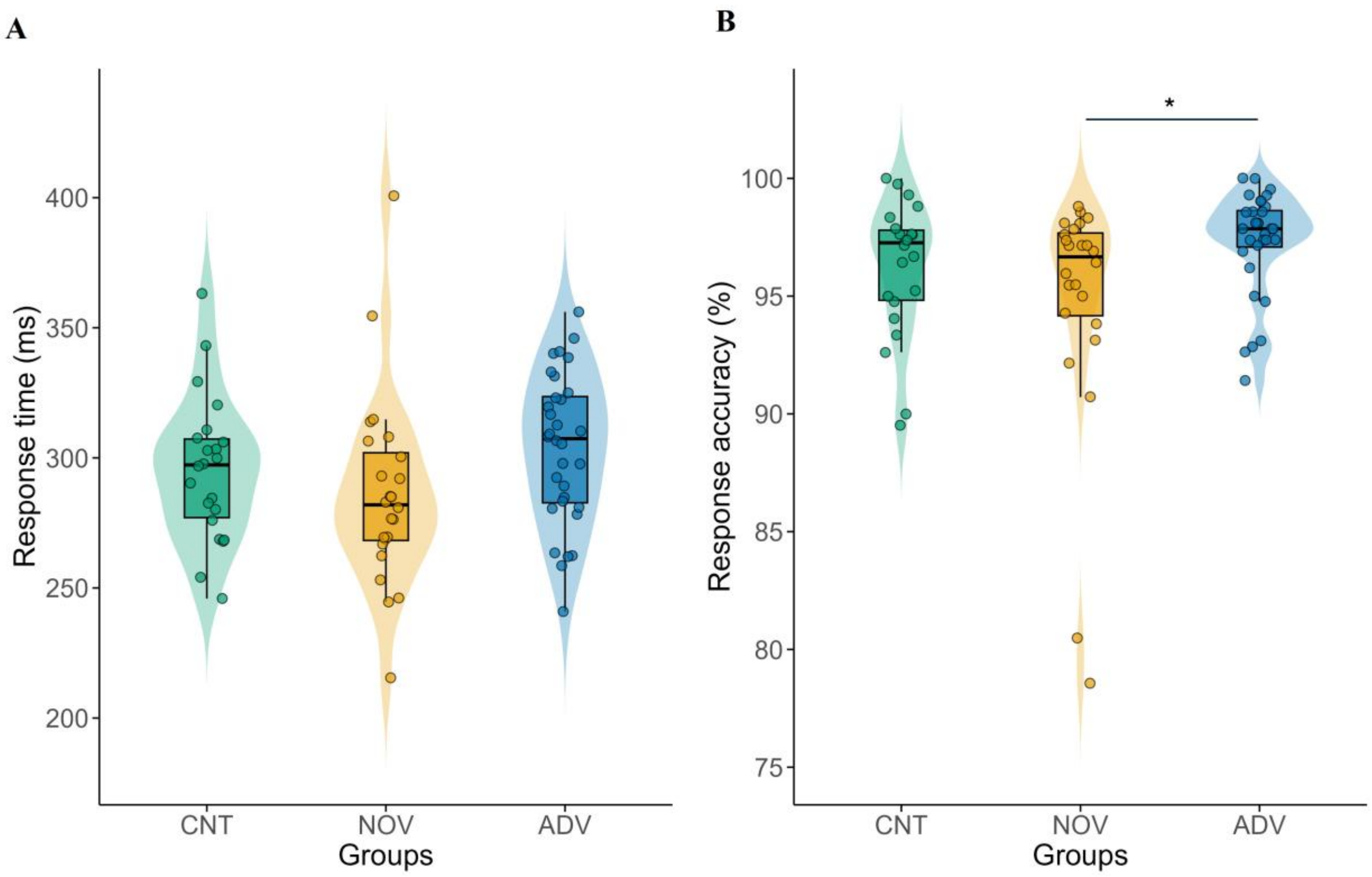

Supplementary Figure 2: A. No Significant differences in response time (in milliseconds) across different groups ($\chi^2_{\text{Kruskal-Wallis}}(2) = 5.89$, $p = 0.05$, $\epsilon^2_{\text{ordinal}} = 0.08$, $\text{CI}_{95\%}[0.03, 1.00]$), $n = 78$; CNT = 22, NOV = 24; ADV = 32). **B** Significant differences in Response accuracy (percentage) across the same groups ($\chi^2_{\text{Kruskal-Wallis}}(2) = 7.36$, $p = 0.03$, $\epsilon^2_{\text{ordinal}} = 0.10$, $\text{CI}_{95\%}[0.02, 1.00]$, $n = 78$; CNT = 22, NOV = 24; ADV = 32). Advanced meditators show highest response accuracy. The boxplot centre line indicates the median, the boxes represent the interquartile range (IQR), and the whiskers extend to 1.5 × IQR. Significance levels: *$p < 0.05$. CNT: Controls, NOV: Novice meditators, ADV: Advanced meditators.

Supplementary Table 4: Post-hoc pair wise comparisons for rest eyes closed vs breath-watching meditation Mixed ANOVA results

Contrast	Estimate	SE	df	t-ratio	p-value
ADV RestEC1 − CNT RestEC1	0.00803	0.01170	76	0.686	0.9830
ADV RestEC1 − NOV RestEC1	-0.00246	0.01230	76	-0.200	1.0000
**ADV RestEC1 − ADV BreathWatch**	**-0.00049**	**0.00911**	**76**	**-0.053**	**1.0000**
ADV RestEC1 − CNT BreathWatch	-0.02656	0.01330	76	-1.999	0.3523
ADV RestEC1 − NOV BreathWatch	-0.03100	0.01400	76	-2.208	0.2464
CNT RestEC1 − NOV RestEC1	-0.01048	0.01270	76	-0.823	0.9624

Contrast	Estimate	SE	df	t-ratio	p-value
CNT RestEC1 − ADV BreathWatch	-0.00851	0.01300	76	-0.653	0.9864
**CNT RestEC1 − CNT BreathWatch**	**-0.03458**	**0.00995**	**76**	**-3.477**	**0.0105**
CNT RestEC1 − NOV BreathWatch	-0.03902	0.01450	76	-2.699	0.0873
NOV RestEC1 − ADV BreathWatch	0.00197	0.01350	76	0.145	1.0000
NOV RestEC1 − CNT BreathWatch	-0.02410	0.01420	76	-1.696	0.5386
**NOV RestEC1 − NOV BreathWatch**	**-0.02854**	**0.01080**	**76**	**-2.639**	**0.1003**
ADV BreathWatch − CNT BreathWatch	-0.02607	0.01450	76	-1.801	0.4715
ADV BreathWatch − NOV BreathWatch	-0.03051	0.01520	76	-2.010	0.3463
CNT BreathWatch − NOV BreathWatch	-0.00444	0.01580	76	-0.282	0.9998

Supplementary Table 5: Results of the Mixed ANOVA Examining the Effects of Group and Condition (Rest, Visual Oddball External Task) on ACW Mean

Effect	Sum_Sq	df_num	Error_SS	df_den	F_value	p_value
Intercept	0.51907	1	0.125981	61	251.3328	< 2.2e-16 ****
Groups	0.00278	2	0.125981	61	0.6739	0.5135 ^{ns}
Condition	0.02399	1	0.076166	61	19.2139	0.00004689 ****
Interaction (Groups × Condition)	0.00044	2	0.076166	61	0.1753	0.8396 ^{ns}

*This table presents the results of a mixed ANOVA assessing the main effects of Group (Controls, Novice Meditators, Advanced Meditators) and Condition (Rest, Visual oddball cognitive task) and their interaction on ACW mean. The table includes the sum of squares (Sum_Sq), numerator degrees of freedom (df_num), error sum of squares (Error_SS), denominator degrees of freedom (df_den), F-values (F_value), and corresponding p-values (p_value). Significance levels are denoted as: ns = Not significant ($p \geq 0.05$), **** = $p < 0.0001$.*

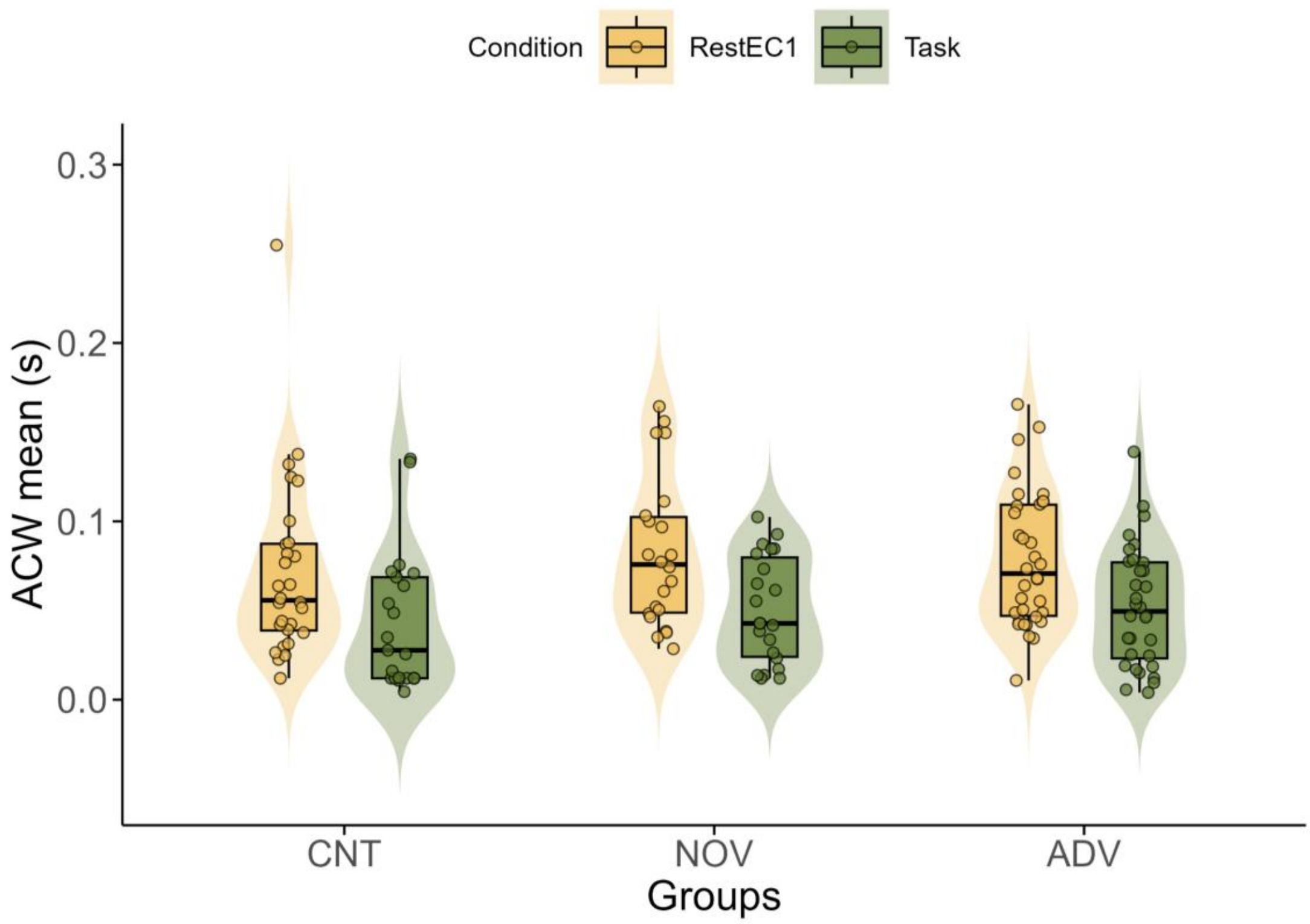

Supplementary Figure 3: The difference in ACW mean between eyes closed resting state and visual oddball cognitive task. The boxplot centre line indicates the median, the boxes represent the interquartile range (IQR), and the whiskers extend to 1.5 × IQR. Statistics are given in Supplement Table 5. CNT: Controls, NOV: Novice meditators, ADV: Advanced meditators.

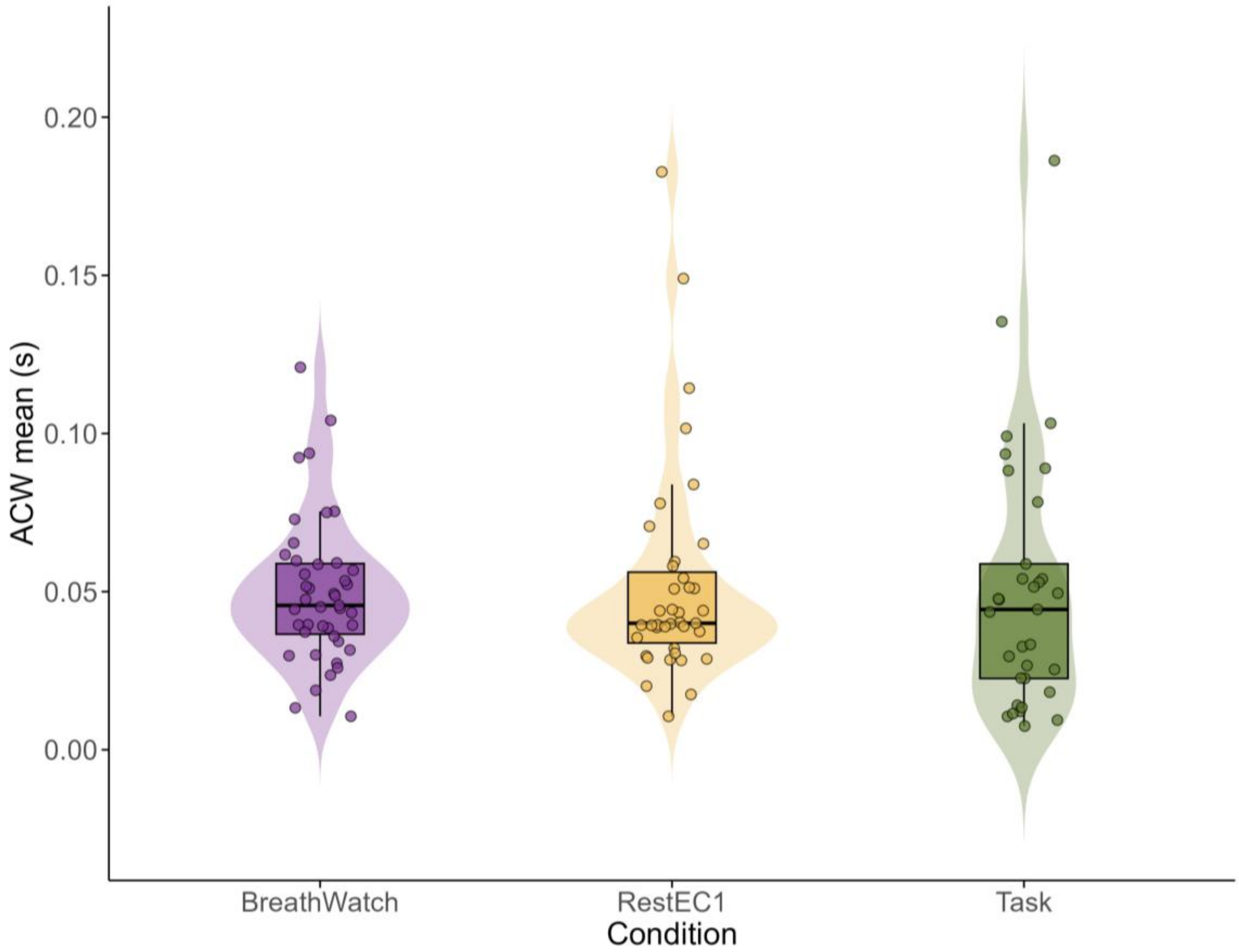

Supplementary Figure 4: No significant difference in ACW mean duration between internal-attention breath-watching meditation, rest eyes closed and external-attention visual oddball cognitive task in an independent sample of expert meditators from the Vipassana tradition ($F(2, 64.68) = 0.06$, $p = 0.95$, $CI_{95\%}[0.00, 1.00]$, $n = 115$; BreathWatch = 43, RestEC1 = 39, Task = 33)). RestEC1 = Rest Eyes Closed. The boxplot centre line indicates the median, the boxes represent the interquartile range (IQR), and the whiskers extend to 1.5 × IQR.

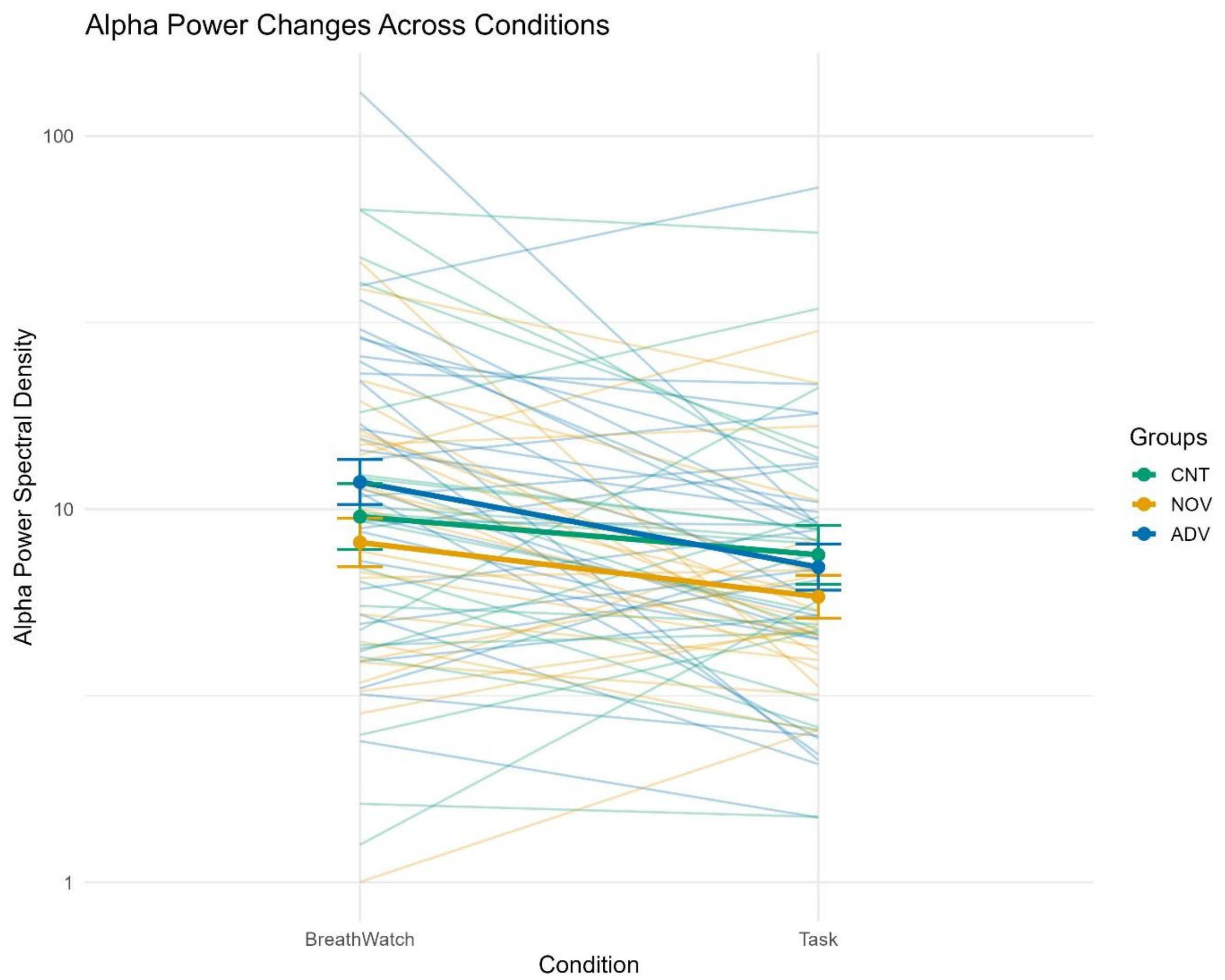

Supplementary Figure 5: Alpha power across breath-watching (eyes closed) and cognitive task (eyes open) conditions in advanced meditators (ADV), controls (CNT), and novices (NOV). Thin lines represent individual participants, and thicker lines with points indicate group means. Error bars denote standard error of the mean. Alpha power is shown on a logarithmic scale.

Supplementary Table 6: Mixed ANOVA of alpha power across group and condition

	**Effect**	**df (num, den)**	**MSE**	**F**	**Effect size (ges)**	**p value**
1	Group	2,72	325.37	0.88	0.016	0.418
2	Condition	1,72	169.96	8.22	0.038	0.005**
3	**Group × Condition**	2,72	169.96	0.32	0.003	**0.725**

Results of a mixed-design ANOVA testing the effects of Group, Condition, and their interaction on the dependent variable. Effect sizes are reported as generalized eta squared (ges). Significance levels: **p < .01. df: degrees of freedom; MSE: mean squared error.

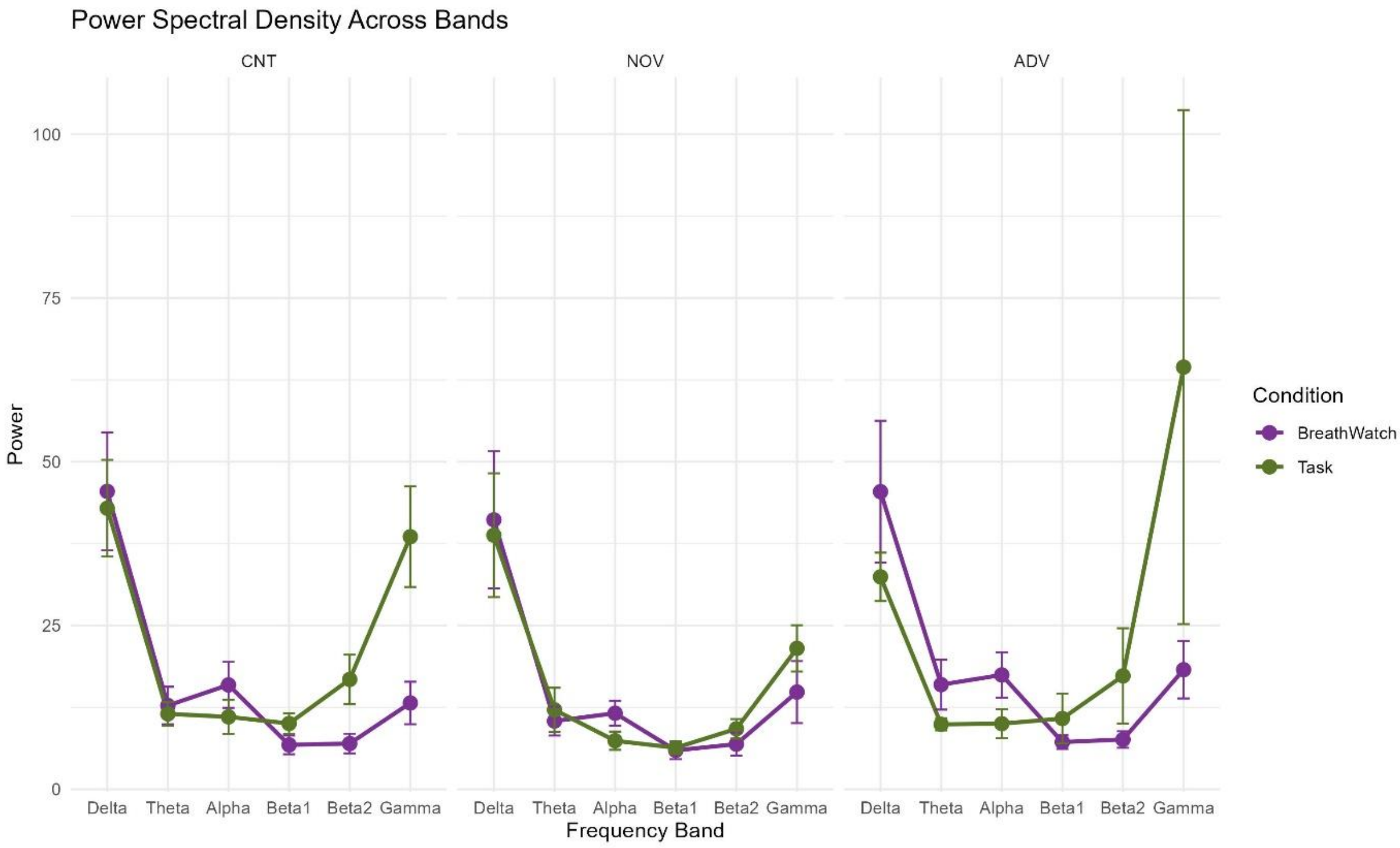

Supplementary Figure 6: Power spectral density across frequency bands for breath-watching and task conditions in controls (CNT), novices (NOV), and advanced meditators (ADV). Points represent group means, and error bars denote standard error of the mean. Colours indicate condition (breath-watching and task).

Supplementary Table 7: Linear mixed-effects model analysis of ACW with group, condition, and alpha power as a covariate

**Effect**	**df (num, den)**	**F**	**p-value**
Group	2, 94.79	0.70	0.497
Condition	1, 92.25	47.09	< 0.001***
Alpha (covariate)	1, 163.78	2.05	0.154
**Group × Condition**	2, 87.68	3.21	**0.045***

Type III sums of squares were used. Degrees of freedom were estimated using Satterthwaite's method. Significance codes: *** p < .001, * p < .05; non-significant effects are not marked.

Supplementary Table 8: Mixed ANOVA of power spectral density across group, condition, and frequency band

	**Effect**	**df (num, den)**	**MSE**	**F**	**Effect size (ges)**	**p-value**
1	Group	2, 72	5445.15	0.70	0.004	0.499
2	Condition	1, 72	4445.31	0.64	0.001	0.428
3	Group × Condition	2, 72	4445.31	0.09	< 0.001	0.915
4	Frequency Band	1.38, 99.38	8158.62	11.40	0.057	< 0.001***
5	Group × Band	2.76, 99.38	8158.62	0.52	0.005	0.657
6	Condition × Band	1.37, 98.73	6243.47	3.08	0.012	0.069^{+}
7	**Group × Condition × Band**	2.74, 98.73	6243.47	0.60	0.005	**0.602**

Effect sizes are reported as generalized eta squared (ges). Degrees of freedom were corrected using the Greenhouse–Geisser method where appropriate. Significance levels: *** p < .001, + p < .10. MSE: mean squared error.

Supplementary Table 9: Linear mixed-effects model analysis of ACW across group and condition with spectral power covariates

**Effect**	**df (num, den)**	**F**	**p-value**
Group	2, 95.08	1.41	0.250
Condition	1, 106.66	44.19	< 0.001***
Delta	1, 157.18	50.94	< 0.001***
Theta	1, 154.49	2.16	0.144
Alpha	1, 159.06	8.41	0.004**
Beta1	1, 139.05	6.30	0.013*
Beta2	1, 160.00	7.03	0.009**
Gamma	1, 154.46	24.38	< 0.001***

**Group × Condition**	2, 92.16	3.11	**0.049***

Type III sums of squares were used, and degrees of freedom were estimated using Satterthwaite's method. Significance levels: *** p < .001, ** p < .01, * p < .05; non-significant effects are not marked.

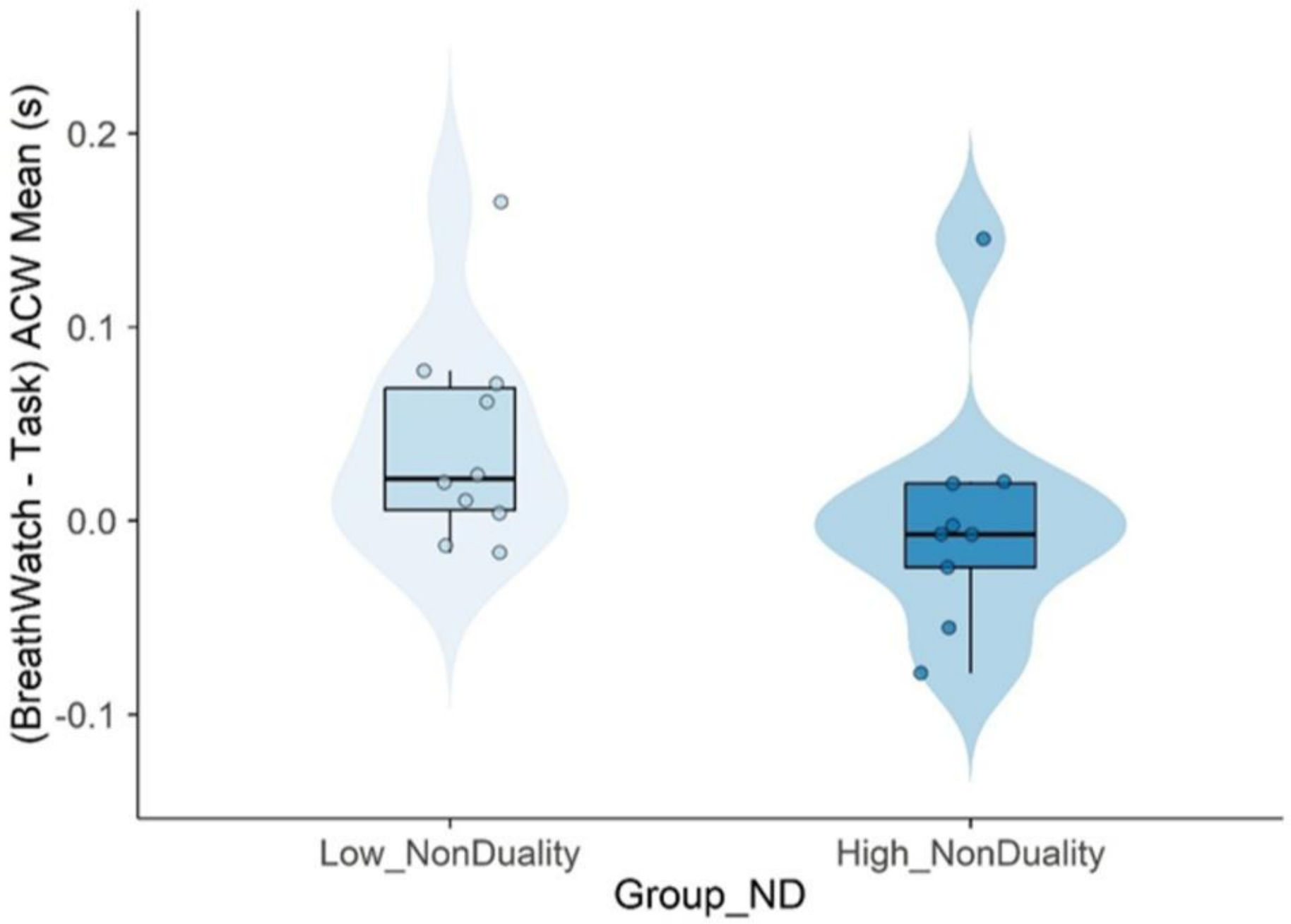

Supplementary Figure 7: Advanced meditators were divided into high and low non-duality groups based on a median split of their non-duality scores. Those with higher non-duality reported smaller internal–external (BreathWatch – Task) ACW differences (p = 0.09, rank-biserial effect size = –0.47, n = 19). The boxplot centre line indicates the median, the boxes represent the interquartile range (IQR), and the whiskers extend to 1.5 × IQR.

Supplementary Table 10: Different components of the MEDEQ predicting Internal-External ACW difference

Predictor	Coefficient	Std Error	t-value	P-value	R-squared
Relaxation	-0.00378	0.00288	-1.3147	0.1945	0.0328
Hindrances	-0.00378	0.00238	-1.5903	0.1179	0.0472
Concentration	-0.00308	0.00147	-2.1005	0.0407*	0.0796
Essential Qualities	-0.00200	0.00107	-1.8641	0.0681	0.0638

Predictor	Coefficient	Std Error	t-value	P-value	R-squared
Non-duality	-0.00259	0.00121	-2.1311	0.0379*	0.0818
Decentering	-0.01582	0.00866	-1.8283	0.0734	0.0615
Meditation Depth (Relax + Hind + Conc + Esse Qual)	-0.00095	0.00047	-2.0222	0.0484*	0.0742
Med Lifetime hours	-0.000002	0.000003	-0.9411	0.3511	0.0171
Age	0.00024	0.00138	0.1744	0.8622	0.0006

This table shows results of different independent linear regression analyses showing the variance in the internal–external ACW difference explained by (predictor): different clusters of the MEDEQ (hindrances, relaxation, concentration, essential qualities, and non-duality), decentering (Q5 and Q10 of MEDEQ), meditation lifetime hours, and age. Significance level is denoted as: * = $p < 0.05$.

Supplementary Table 11: Is non-duality better predicted by the internal-external ACW, internal ACW, or external ACW? Results of independent linear regression models.

Model	Predictor	Estimate (β)	Std. Error	Z-value	p-value	Std. β	R^2
1	Internal–External ACW difference → Non-duality	-31.62	14.84	-2.13	0.033*	-0.286	0.082
2	Internal ACW → Non-duality	-19.30	15.53	-1.24	0.214	-0.171	0.029
3	External ACW → Non-duality	42.58	27.40	1.55	0.120	0.213	0.045

Supplementary Table 12: Hierarchical regression model (Step 1: Internal ACW, Step 2: Internal ACW + ACW difference) predicting non-duality.

Model	Predictor	Estimate	Std Error	t-value	p-value
**Step 1**	Intercept	11.83	1.77	6.67	< .001
	Internal ACW	-19.30	15.53	-1.24	.220
	Model Summary				
	R^2	.029			
	Adjusted R^2	.010			
	$F(1, 51)$	1.55			.220
**Step 2**	Intercept	10.02	1.96	5.13	< .001
	Internal ACW	27.59	28.25	0.98	.333
	ACW difference	-54.53	27.76	-1.97	.055
	Model Summary				
	R^2	.099			
	Adjusted R^2	.063			
	ΔR^2	.070			
	$F(2, 50)$	2.75			.074

Supplementary Table 13: Hierarchical regression model (Step 1: ACW difference, Step 2: ACW difference + Internal ACW) predicting non-duality.

Model	Predictor	Estimate	Std. Error	t value	p-value
**Step 1**	(Intercept)	11.53	1.20	9.60	<.001
	ACW difference	-31.62	14.84	-2.13	.038*
	Model Summary				
	R^2	.082			
	Adjusted R^2	.064			
	$F(1, 51)$	4.54			.038*
**Step 2**	(Intercept)	10.02	1.96	5.13	<.001
	ACW difference	-54.53	27.76	-1.97	.055
	Internal ACW	27.59	28.25	0.98	.333
	Model Summary				
	R^2	.099			
	Adjusted R^2	.063			
	ΔR^2	.017			
	$F(2, 50)$	2.75			.074

Supplementary Table 14: Hierarchical regression model (Step 1: External ACW, Step 2: External ACW + ACW difference) predicting non-duality.

Model	Predictor	Estimate	Std. Error	t value	p-value
**Step 1**	(Intercept)	8.02	1.61	5.00	<.001
	External ACW	42.58	27.40	1.55	.126
	Model Summary				
	R^2	.045			

Model	Predictor	Estimate	Std. Error	t value	p-value
	Adjusted R^2	.027			
	F(1, 51)	2.42			.126
**Step 2**	(Intercept)	10.02	1.96	5.13	<.001
	External ACW	27.59	28.25	0.98	.334
	ACW difference	-26.94	15.60	-1.73	.090
	Model Summary				
	R^2	.099			
	Adjusted R^2	.063			
	ΔR^2	.054			
	F(2, 50)	2.75			.074

Supplementary Table 15: Hierarchical regression model (Step 1: ACW difference, Step 2: ACW difference + External ACW) predicting non-duality.

Model	Predictor	Estimate	Std. Error	t value	p-value
**Step 1**	(Intercept)	11.53	1.20	9.60	< .001
	ACW difference	-31.62	14.84	-2.13	.038
	Model Summary				
	R^2	.082			
	Adjusted R^2	.064			
	F(1, 51)	4.54			.038
**Step 2**	(Intercept)	10.02	1.96	5.13	< .001
	ACW difference	-26.94	15.60	-1.73	.090
	External ACW	27.59	28.25	0.98	.334
	Model Summary				
	R^2	.099			
	Adjusted R^2	.063			

Model	Predictor	Estimate	Std. Error	t value	p-value
	ΔR^2	.017			
	F(2, 50)	2.75			.074

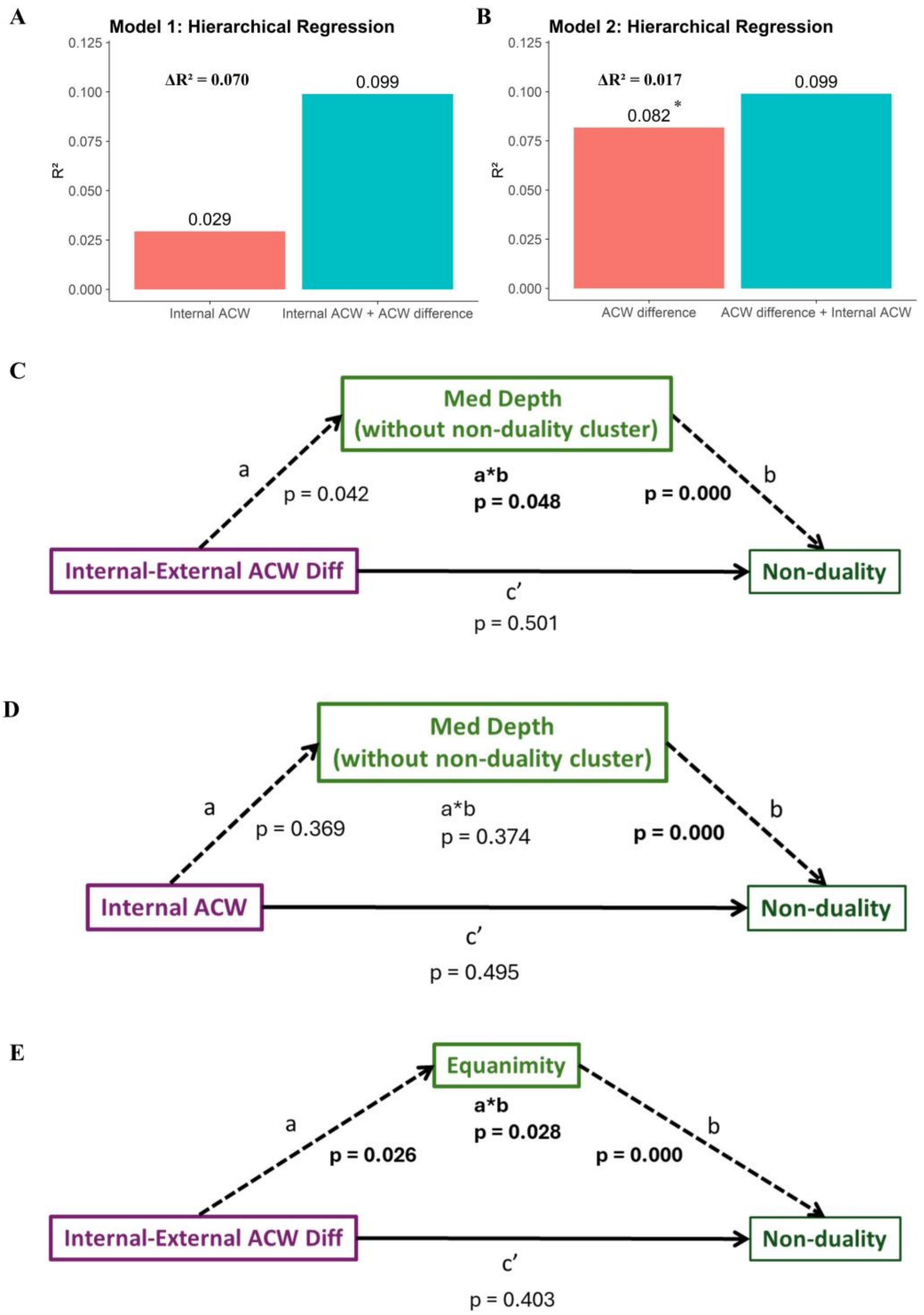

Supplementary Figure 8: Is non-duality better predicted by the internal-external ACW or by internal ACW? A In the first model, Internal ACW was entered in Step 1 to predict non-duality, but

this was not significant ($F(1,51) = 1.55$, $R^2 = 0.029$, $p = 0.220$). When Internal–External ACW difference was added in Step 2, the explained variance increased, reaching trend significance ($F(2,50) = 2.75$, $R^2 = 0.099$, $\Delta R^2 = 0.070$, $p = 0.074$). **B** In the second hierarchical regression model, the order was reversed: Internal–External ACW difference was entered first (Step 1), which yielded a significant result ($F(1, 51) = 4.54$, $R^2 = 0.082$, $p = 0.038$). However, when Internal ACW was added in Step 2, the overall model became non-significant ($F(2,50) = 2.75$, $R^2 = 0.099$, $\Delta R^2 = 0.017$, $p = 0.074$). **C** Mediation analysis (c': direct path, (a*b): indirect path) demonstrating that the relationship between internal–external ACW difference and non-duality is mediated by other meditation depth clusters. In this model, a*b is significant, indicating full mediation. **D** Internal ACW does not predict non-duality, even when mediation through other meditation depth clusters is included. **E** Equanimity significantly mediates the relationship between internal–external ACW difference and non-duality. In this model, a*b is significant, indicating full mediation.

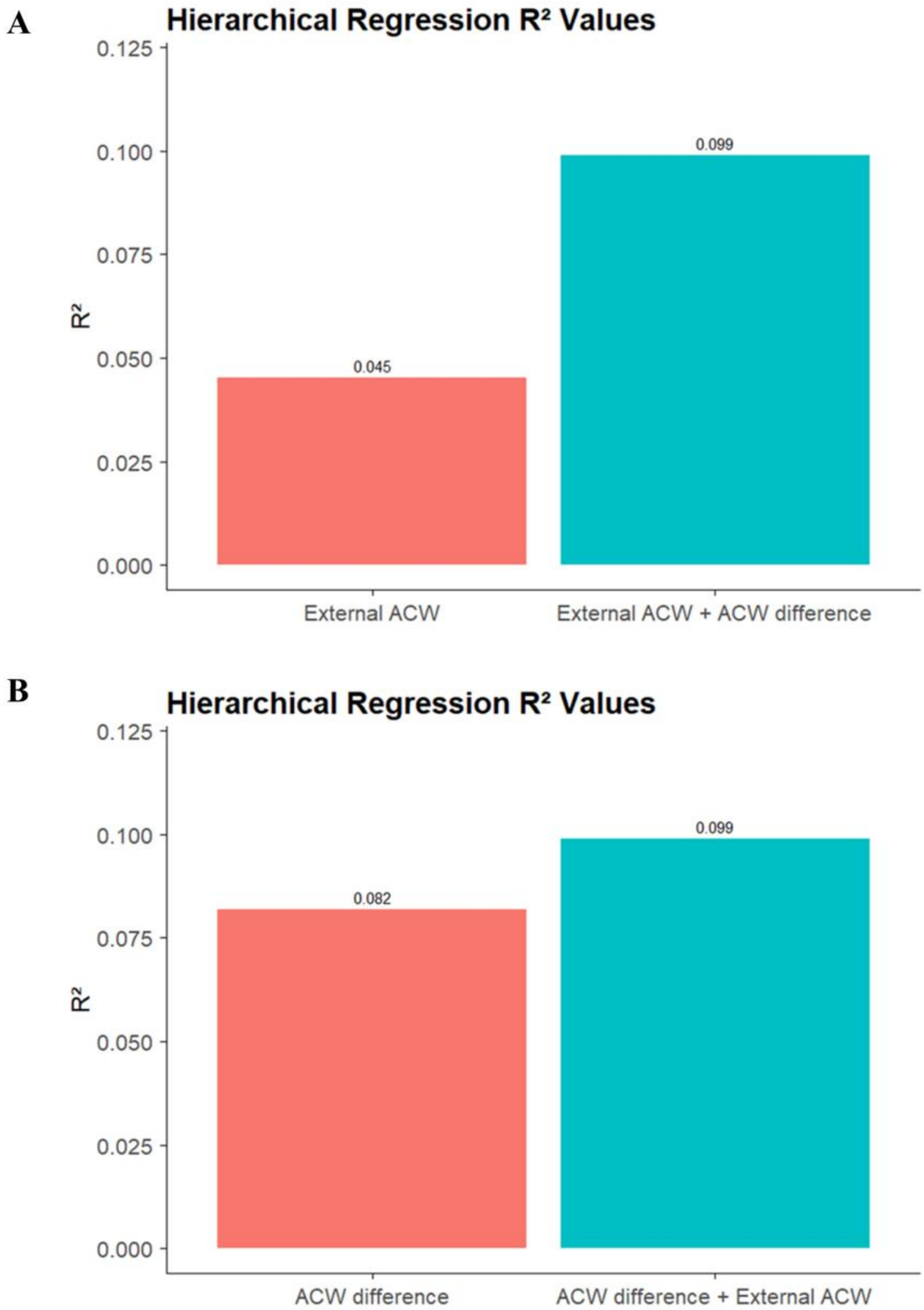

Supplementary Figure 9: Which Better Predicts Non-Duality: Internal–External ACW Difference or External ACW? A In the first model, External ACW was entered in Step 1 to predict non-duality, but this was not significant ($R^2 = 0.045$). When Internal–External ACW difference was added in Step 2, the explained variance increased ($R^2 = 0.099$). **B** In the second model, Internal-External ACW difference was added in Step 1 ($R^2 = 0.082$), while External ACW was added in Step 2 ($R^2 = 0.082$).

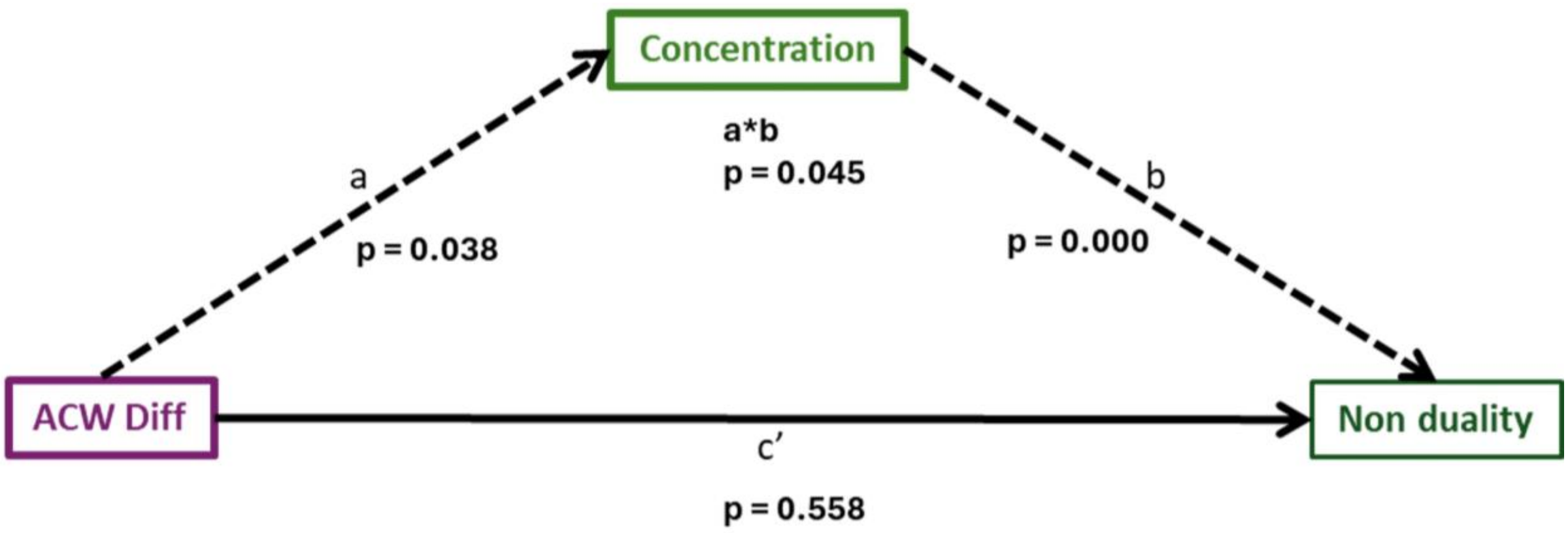

Supplementary Figure 10: Mediation analysis demonstrating that the relationship between internal–external ACW difference and non-duality is mediated by the concentration cluster. In this model, a*b is significant, indicating full mediation.

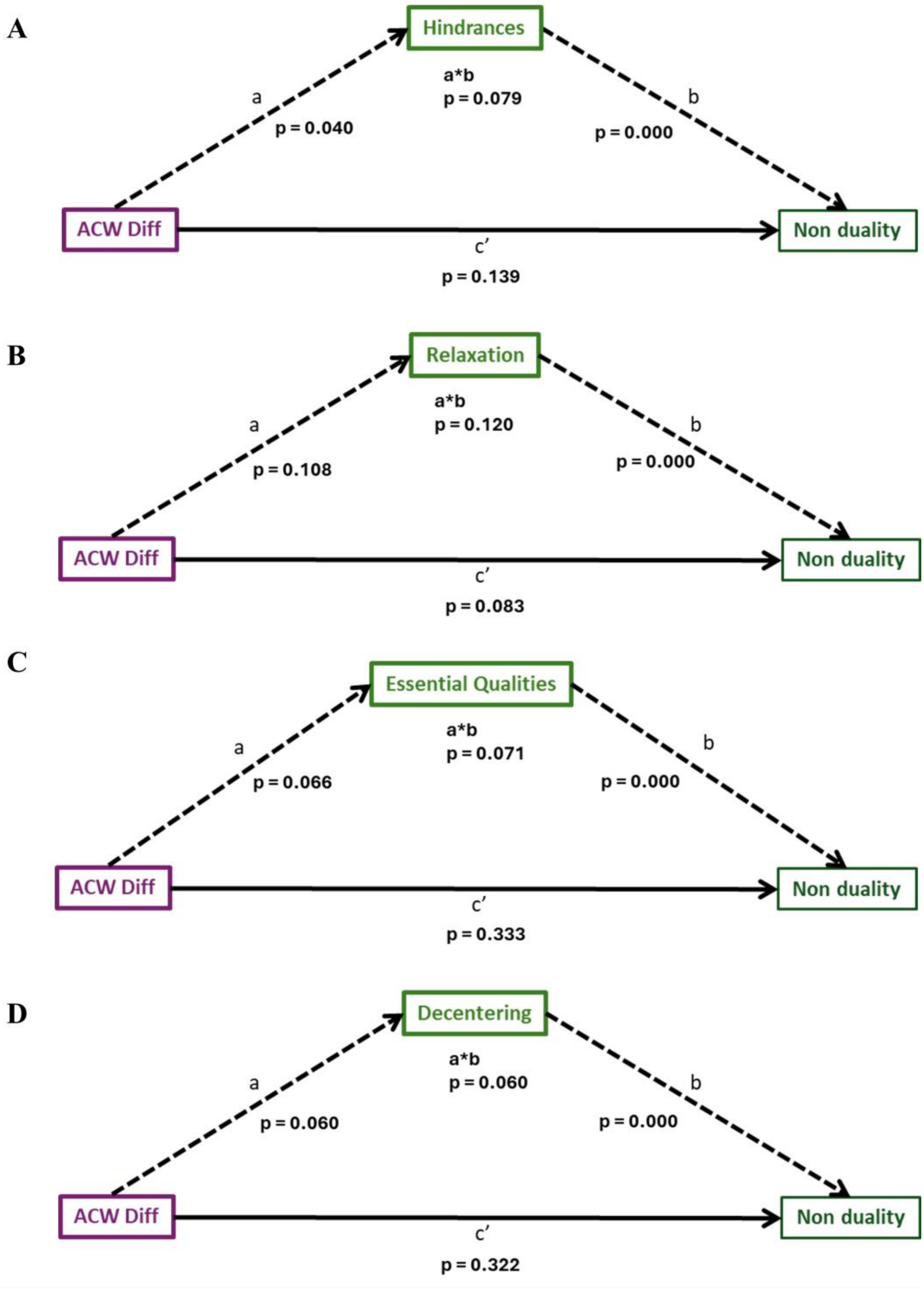

Supplementary Figure 11: Mediation analysis demonstrating that the relationship between internal–external ACW difference and non-duality is not mediated by hindrances (**A**), relaxation (**B**), essential qualities (**C**), and decentering (**D**).

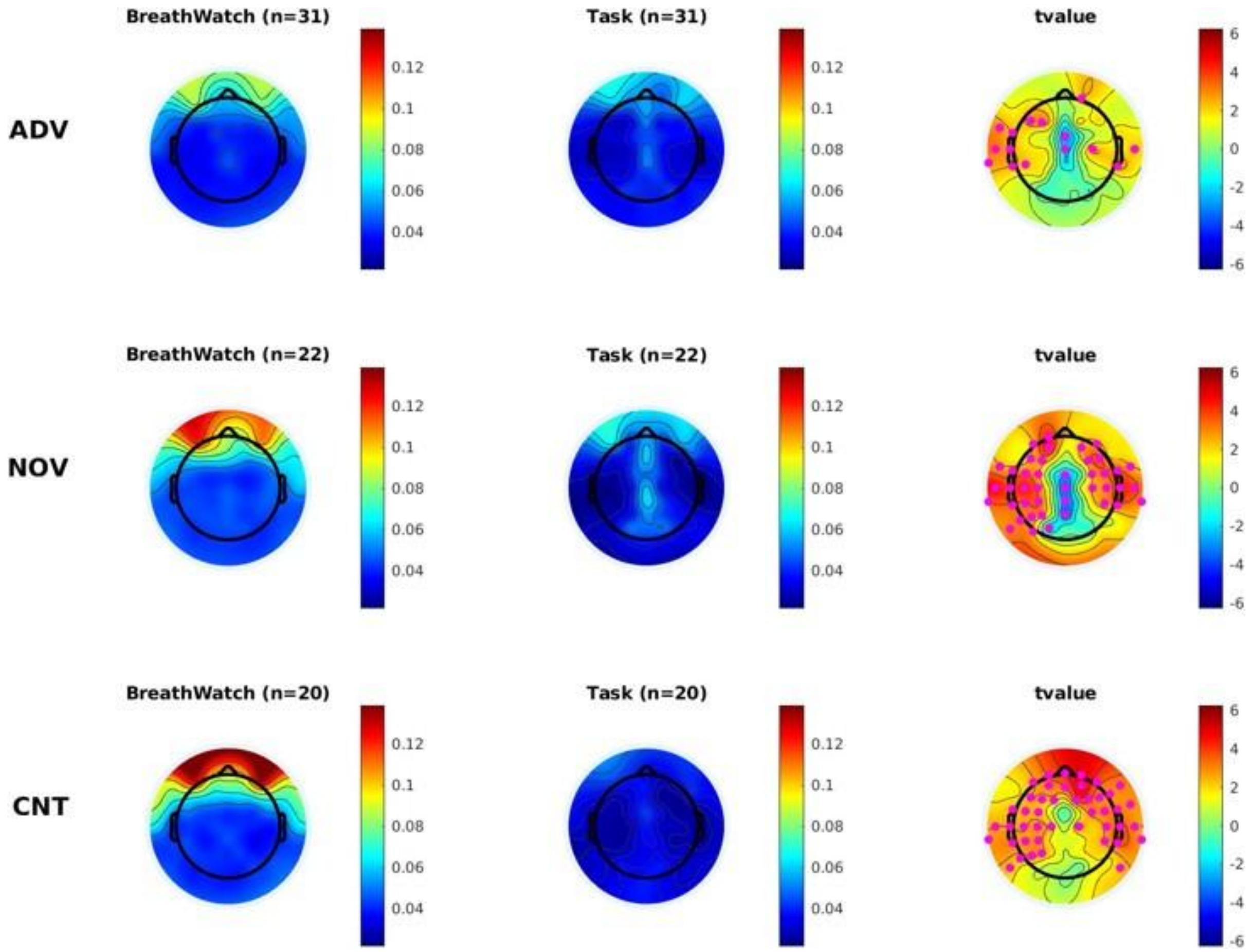

Supplementary Figure 12: Topographic maps of ACW during breath-watching and cognitive task conditions in advanced meditators (ADV), novices (NOV), and controls (CNT). ACW was computed for 10s epochs with 1s window and 50% overlap. The first and second columns show condition-specific maps, and the third column shows t-values for the contrast (breath-watching − task). Robust paired t-tests with threshold-free cluster enhancement (TFCE) were used. Statistically significant electrodes are indicated in pink.

Supplementary References

1. Malipeddi, S., Mehrotra, S., John, J. P. & Kutty, B. M. Practice and proficiency of Isha Yoga for better mental health outcomes: insights from a COVID-19 survey. *Frontiers in Public Health* **12**, (2024).
2. Malipeddi, S. *et al.* Relaxed Alertness in Novice and Advanced Meditators – A Neurophysiological and Psychological Study of Isha Yoga Practices. *Mindfulness* **15**, 2840–2859 (2024b).
3. Sadhguru, J. V. Inner engineering. (2016).
4. Chandran, V. *et al.* Large-scale genomic study reveals robust activation of the immune system following advanced Inner Engineering meditation retreat. *Proceedings of the National Academy of Sciences* **118**, e2110455118 (2021).
5. Sadhguru. Official Website of Sadhguru, Isha Foundation | India. https://isha.sadhguru.org/in/en/home.
6. Laukkonen, R. E. & Slagter, H. A. From many to (n)one: Meditation and the plasticity of the predictive mind. *Neuroscience & Biobehavioral Reviews* **128**, 199–217 (2021).
7. Dunne, J. D. Buddhist Styles of Mindfulness: A Heuristic Approach. in *Handbook of Mindfulness and Self-Regulation* (eds Ostafin, B. D., Robinson, M. D. & Meier, B. P.) 251–270 (Springer New York, New York, NY, 2015). doi:10.1007/978-1-4939-2263-5_18.
8. Dahl, C. J., Lutz, A. & Davidson, R. J. Reconstructing and deconstructing the self: cognitive mechanisms in meditation practice. *Trends in Cognitive Sciences* **19**, 515–523 (2015).
9. Lutz, A., Jha, A. P., Dunne, J. D. & Saron, C. D. Investigating the phenomenological matrix of mindfulness-related practices from a neurocognitive perspective. *Am Psychol* **70**, 632–658 (2015).

10. Hölzel, B. K. *et al.* How Does Mindfulness Meditation Work? Proposing Mechanisms of Action From a Conceptual and Neural Perspective. *Perspect Psychol Sci* **6**, 537–559 (2011).

11. Braboszcz, C., Cahn, B. R., Levy, J., Fernandez, M. & Delorme, A. Increased gamma brainwave amplitude compared to control in three different meditation traditions. *PLOS ONE* **12**, e0170647 (2017).

12. Chang, T. F. H. An Emerging Positive Intervention—Inner Engineering Online (IEO). *Journal of Occupational and Environmental Medicine* **63**, e657 (2021).

13. Sadhasivam, S. *et al.* Inner Engineering Practices and Advanced 4-day Isha Yoga Retreat Are Associated with Cannabimimetic Effects with Increased Endocannabinoids and Short-Term and Sustained Improvement in Mental Health: A Prospective Observational Study of Meditators. *Evidence-Based Complementary and Alternative Medicine* **2020**, e8438272 (2020).

14. Braboszcz, C. *et al.* Plasticity of visual attention in Isha yoga meditation practitioners before and after a 3-month retreat. *Frontiers in Psychology* **4**, (2013).

15. Saketh, M. *et al.* Temporal EEG Signatures of Meditation Experience: Peak Brainwave Changes at 7 Minutes During Isha Yoga Breath Watching. *Mindfulness* **17**, 762–778 (2026).

16. Peterson, C. T., Bauer, S. M., Chopra, D., Mills, P. J. & Maturi, R. K. Effects of Shambhavi Mahamudra Kriya, a multicomponent breath-based yogic practice (pranayama), on perceived stress and general well-being. *J Evid Based Complementary Altern Med* **22**, 788–797 (2017).

17. Selvaraj, N. *et al.* Heart Rate Dynamics during Shambhavi Mahamudra- A Practice of Isha Yoga. *Journal of Complementary and Integrative Medicine* **5**, (2008).

18. Sadhasivam, S. *et al.* Isha Yoga practices and participation in Samyama program are associated with reduced HbA1C and systemic inflammation, improved lipid profile, and

short-term and sustained improvement in mental health: A prospective observational study of meditators. *Front Psychol* **12**, 659667 (2021).

19. Sadhguru. Inner Engineering | Shambhavi Mahamudra Kriya | Offered by Sadhguru. https://isha.sadhguru.org/in/en/inner-engineering.
20. Sadhguru. Shoonya Intensive | Isha Yoga | Sadhguru. https://isha.sadhguru.org/in/en/yoga-meditation/yoga-program-advanced/shoonya-meditation.
21. Raman, M. *et al.* Isha yoga practices, vegan diet, and participation in Samyama meditation retreat: impact on the gut microbiome & metabolome - a non-randomized trial. *BMC Complement Med Ther* **23**, 107 (2023).
22. Sadhguru. Samyama. https://isha.sadhguru.org/in/en/yoga-meditation/yoga-program-advanced/samyama.
23. Carneiro, É. M., Moraes, G. V. & Terra, G. A. Effects of Isha Hatha Yoga on Core Stability and Standing Balance. *Adv Mind Body Med* **30**, 4–10 (2016).
24. Sadhguru. What Is Hatha Yoga? https://isha.sadhguru.org/in/en/yoga-meditation/yoga-program-for-beginners/hatha-yoga.
25. Malipeddi, S. *et al.* Time-to-onset and temporal dynamics of EEG during breath-watching meditation. 2025.02.11.637771 Preprint at https://doi.org/10.1101/2025.02.11.637771 (2025).
26. Iyengar, B. K. S. Light on the yoga sutras of Patanjali. *Philosophy East and West* **46**, 291 (1996).
27. Reddy, J. S. K. & Roy, S. Commentary: Patanjali and neuroscientific research on meditation. *Front Psychol* **9**, 248 (2018).
28. Josipovic, Z. Implicit–explicit gradient of nondual awareness or consciousness as such. *Neurosci Conscious* **2021**, niab031 (2021).

29. Josipovic, Z. & Miskovic, V. Nondual Awareness and Minimal Phenomenal Experience. *Front. Psychol.* **11**, (2020).
30. Sadhguru. Tools to Tap the Source of Creation. https://isha.sadhguru.org/linga-bhairavi/in/en/article/tools-tap-source-creation (2015).
31. Tang, Y.-Y., Hölzel, B. K. & Posner, M. I. The neuroscience of mindfulness meditation. *Nat Rev Neurosci* **16**, 213–225 (2015).
32. Josipovic, Z. Chapter 12 - Nondual awareness: Consciousness-as-such as non-representational reflexivity. in *Progress in Brain Research* (ed. Srinivasan, N.) vol. 244 273–298 (Elsevier, 2019).
33. Cooper, A. C., Ventura, B. & Northoff, G. Beyond the veil of duality—topographic reorganization model of meditation. *Neuroscience of Consciousness* **2022**, niac013 (2022).
34. Swami Nikhilananda. *The Upanishads: Vol. I-IV*. (Ramakrishna Vivekanada Center, 1987).
35. Radhakrishnan and Moore. A Sourcebook in Indian Philosophy | Princeton University Press. https://press.princeton.edu/books/paperback/9780691019581/a-sourcebook-in-indian-philosophy (1957).
36. Sadhguru. What is Consciousness? 6 Myths Busted and 4 States Explained. https://isha.sadhguru.org/en/wisdom/article/what-is-consciousness-states-myths (2021).
