# Supplementary material for: Non-duality in brain and experience of advanced meditators—key role for intrinsic neural timescales
Source: Commun Biol. 2026 Jun 12;9:1261. doi: 10.1038/s42003-026-10473-9 (PMC13616908; doi:10.1038/s42003-026-10473-9)
Supplement: Supplementary file 3 [file 42003_2026_10473_MOESM3_ESM.pdf]

Description of Additional Supplementary Files

File name: Supplementary data 1
Description: Source data supporting the figures in the paper.
