# Supplementary material for: Non-duality in brain and experience of advanced meditators—key role for intrinsic neural timescales
Source: Commun Biol. 2026 Jun 12;9:1261. doi: 10.1038/s42003-026-10473-9 (PMC13616908; doi:10.1038/s42003-026-10473-9)
Supplement: Supplementary file 4 [file 42003_2026_10473_MOESM5_ESM.pdf]

nature portfolio

Corresponding author(s): Saketh Malipeddi, Georg Northoff

Last updated by author(s): May 20, 2026

Reporting Summary

Nature Portfolio wishes to improve the reproducibility of the work that we publish. This form provides structure for consistency and transparency in reporting. For further information on Nature Portfolio policies, see our Editorial Policies and the Editorial Policy Checklist.

Statistics

For all statistical analyses, confirm that the following items are present in the figure legend, table legend, main text, or Methods section.

n/a	Confirmed	
☐	☒	The exact sample size (*n*) for each experimental group/condition, given as a discrete number and unit of measurement
☐	☒	A statement on whether measurements were taken from distinct samples or whether the same sample was measured repeatedly
☐	☒	The statistical test(s) used AND whether they are one- or two-sided<br>*Only common tests should be described solely by name; describe more complex techniques in the Methods section.*
☒	☐	A description of all covariates tested
☐	☒	A description of any assumptions or corrections, such as tests of normality and adjustment for multiple comparisons
☐	☒	A full description of the statistical parameters including central tendency (e.g. means) or other basic estimates (e.g. regression coefficient) AND variation (e.g. standard deviation) or associated estimates of uncertainty (e.g. confidence intervals)
☐	☒	For null hypothesis testing, the test statistic (e.g. *F*, *t*, *r*) with confidence intervals, effect sizes, degrees of freedom and *P* value noted<br>*Give P values as exact values whenever suitable.*
☒	☐	For Bayesian analysis, information on the choice of priors and Markov chain Monte Carlo settings
☒	☐	For hierarchical and complex designs, identification of the appropriate level for tests and full reporting of outcomes
☐	☒	Estimates of effect sizes (e.g. Cohen's *d*, Pearson's *r*), indicating how they were calculated

Our web collection on statistics for biologists contains articles on many of the points above.

Software and code

Policy information about availability of computer code

Data collection	We used Net Station 5.4 software (EGI, Philips Neuro, USA) for EEG data acquisition and E-prime 2.0 software (Psychology Softwar Tools, Inc., Sharpsburg, PA, USA) for paradigm presentation.
Data analysis	EEG data analysis was done using EEGLAB v2021.0 open source toolbox. An autocorrelation function (ACF) was calculated in MATLAB (version 2023b) using an open-source code (https://www.georgnorthoff.com/code). For statistical analysis, we used RStudio Version 1.4.1106, employing several open source R packages including ggplot, ggsci, ggstatsplot, dlpyr, effectsize, performance, and car for necessary analyses.

For manuscripts utilizing custom algorithms or software that are central to the research but not yet described in published literature, software must be made available to editors and reviewers. We strongly encourage code deposition in a community repository (e.g. GitHub). See the Nature Portfolio guidelines for submitting code & software for further information.

Data

Policy information about availability of data

All manuscripts must include a data availability statement. This statement should provide the following information, where applicable:

- Accession codes, unique identifiers, or web links for publicly available datasets
- A description of any restrictions on data availability
- For clinical datasets or third party data, please ensure that the statement adheres to our policy

The human EEG data are not publicly available due to ethical requirements and privacy concerns. The numerical data that can be used to generate figures are within Supplementary Information.

Research involving human participants, their data, or biological material

Policy information about studies with human participants or human data. See also policy information about sex, gender (identity/presentation), and sexual orientation and race, ethnicity and racism.

Reporting on sex and gender	- Findings apply to both genders.<br>- Gender-matching wad done.<br>- Gender was determined by self-reporting.<br>- Consent was obtained to share deidentified individual-level data.<br>- Overall numbers: Advanced meditators (M/F) - 24/18; Novice meditators (M/F) - 19/14; Controls (M/F) - 12/16<br>- No gender based analysis was performed as the primary objective was investigating neural signatures of non-duality at a group level.
Reporting on race, ethnicity, or other socially relevant groupings	- None
Population characteristics	- Mean age (SD) for advanced meditators - 35.57 (6.81); novice meditators - 31.66 (7.64); and controls - 31.14 (6.38)<br>- Advanced meditators had 24 males and 18 females. Novices had 19 males and 14 females. Controls had 12 males and 16 females.<br>- All participants were healthy.
Recruitment	Meditators were recruited from the meditation community in Bangalore, India, through email and WhatsApp communication. Control participants were recruited from the local university community where the study was conducted. While self-report questionnaires may introduce potential biases, these can be mitigated by the inclusion of objective measures, such as EEG, which we used in the study.
Ethics oversight	National Institute of Mental Health and Neurosciences (NIMHANS), Bangalore, India.

Note that full information on the approval of the study protocol must also be provided in the manuscript.

Field-specific reporting

Please select the one below that is the best fit for your research. If you are not sure, read the appropriate sections before making your selection.

☒ Life sciences ☐ Behavioural & social sciences ☐ Ecological, evolutionary & environmental sciences

For a reference copy of the document with all sections, see nature.com/documents/nr-reporting-summary-flat.pdf

Life sciences study design

All studies must disclose on these points even when the disclosure is negative.

Sample size	The sample size was estimated using Gpower software (alpha = 0.05, power = 0.80, effect size = 0.3) and aligns with standards commonly found in the meditation literature.
Data exclusions	Data were excluded only when the EEG data quality was poor for analyses. Bad channels and segments were automatically rejected using the artifact subspace reconstruction (ASR) plugin with a five standard deviation threshold.
Replication	As this was a cross-sectional study, replication was not conducted. However, we replicated our main findings in an another dataset of advanced meditators.
Randomization	Not applicable as this was a cross-sectional study.
Blinding	Not applicable as this was a cross-sectional study.

Reporting for specific materials, systems and methods

We require information from authors about some types of materials, experimental systems and methods used in many studies. Here, indicate whether each material, system or method listed is relevant to your study. If you are not sure if a list item applies to your research, read the appropriate section before selecting a response.

Materials & experimental systems

n/a	Involved in the study	
☒	☐	Antibodies
☒	☐	Eukaryotic cell lines
☒	☐	Palaeontology and archaeology
☒	☐	Animals and other organisms
☒	☐	Clinical data
☒	☐	Dual use research of concern
☒	☐	Plants

Methods

n/a	Involved in the study	
☒	☐	ChIP-seq
☒	☐	Flow cytometry
☒	☐	MRI-based neuroimaging

Plants

Seed stocks	NA
Novel plant genotypes	NA
Authentication	NA
